# Effects of a farm-specific fecal microbial transplant (FMT) product on clinical outcomes and fecal microbiome composition in preweaned dairy calves

**DOI:** 10.1371/journal.pone.0276638

**Published:** 2022-10-21

**Authors:** Giovana S. Slanzon, Benjamin J. Ridenhour, Lindsay M. Parrish, Sophie C. Trombetta, Dale A. Moore, William M. Sischo, Craig S. McConnel

**Affiliations:** 1 Department of Veterinary Clinical Sciences, College of Veterinary Medicine, Washington State University, Pullman, Washington, United States of America; 2 Department of Mathematics and Statistical Science, College of Science, University of Idaho, Moscow, Idaho, United States of America; University of Illinois, UNITED STATES

## Abstract

Gastrointestinal disease (GI) is the most common illness in pre-weaned dairy calves. Therefore, effective strategies to manipulate the microbiome of dairy calves under commercial dairy operations are of great importance to improve animal health and reduce antimicrobial usage. The objective of this study was to develop a farm-specific FMT product and to investigate its effects on clinical outcomes and fecal microbial composition of dairy calves. The FMT product was derived from feces from healthy donors (5–24 days of age) raised in the same calf ranch facility as the FMT recipients. Healthy and diarrheic calves were randomly enrolled to a control (n = 115) or FMT (n = 112) treatment group (~36 g of processed fecal matter once daily for 3 days). Fecal samples were collected at enrollment and again 9 days later after the first FMT dose. Although the FMT product was rich in organisms typically known for their beneficial probiotic properties, the FMT therapy did not prevent or ameliorate GI disease in dairy calves. In fact, calves that received FMT were less likely to recover from GI disease, and more likely to die due to GI disease complications. Fecal microbial community analysis revealed an increase in the alpha-diversity in FMT calves; however, no major differences across treatment groups were observed in the beta-diversity analysis. Calves that received FMT had higher relative abundance of an uncultured organism of the genus *Lactobacillus* and *Lactobacillus reuteri* on day 10. Moreover, FMT calves had lower relative abundance of *Clostridium nexile* and *Bacteroides vulgatus* on day 10. Our results indicate the need to have an established protocol when developing FMT products, based on rigorous inclusion and exclusion criteria for the selection of FMT donors free of potential pathogens, no history of disease or antibiotic treatment.

## Introduction

A fecal microbiota transplant (FMT) is the administration of stool from a healthy donor into a recipient’s intestinal tract aiming to change the microbial composition and gain therapeutic benefit [1]. The gastrointestinal (GI) tract holds a complex and dynamic community of microorganisms, which influences disease and homeostasis of the host [2]. Beneficial microorganisms play an important role in metabolizing energy, maintaining a functional intestinal barrier, defending the host against pathogens, and regulating the innate and adaptive immunological processes [3–6]. An imbalance of the microbial composition of the gut, known as dysbiosis, may disturb essential functions and contribute to the process of promoting disease leading to a pathogenic progression toward several disorders [7].

The microbial composition of the GI tract of calves can change over time [8], and is potentially impacted by diet [9], management practices [10], disease state [11, 12], stressful procedures [13], and genetic factors [14]. Despite variations in the taxonomic composition of individuals, it is commonly recognized that a stable and diverse microbial community is associated with a healthy gut [15] and microbial dysbiosis is linked to a lower microbial diversity [16]. The same pattern has been observed in neonatal dairy calves [11]. Nonetheless, defining the optimal microbiota that supports animal health and performance under commercial conditions is complex, since even healthy individuals can differ remarkably in their microbiome [17].

FMT has been suggested as an effective treatment for GI and non-GI diseases, potentially stabilizing a dysbiotic microbiota even though healthy donors’ stool can be complex and variable [18]. In humans, FMT has been used to treat human *Clostridium difficile* infections with success [19, 20], and there are indications that FMT may also be a therapeutic alternative for other problems such as inflammatory bowel disease and other functional GI disorders [1]. More generally, FMT is being studied as an alternative to antibiotic therapy with the intent of changing the recipient’s microbial composition and confer a health benefit [20]. FMT in veterinary medicine primarily has been applied to oral transfaunation of mature cattle with ruminated ingesta or rumen contents rather than feces [21]. Aside from that application, Rosa et al. [22] assessed the effectiveness of FMT from an adult donor in neonatal dairy calves and suggested that neonatal calves may benefit from FMT in terms of influencing growth and development and possibly alleviating stress caused by weaning procedures. Moreover, the use of FMT in beef calves recently has been reported to ameliorate diarrhea and possibly improve growth performance [23]. Microbial manipulation using FMT has been reported in other fields of animal production as well, primarily with an intent to enhance animals’ growth performance when administrated in early in life [24–26]. In fact, FMT has been highlighted as a promising alternative technology to antibiotics for use in animal health and production [27].

Finding alternatives to antibiotics to prevent or treat GI disease in early-weaned farm animals is crucial for improving animal health and reducing antimicrobial-resistant bacterial phenotypes. GI disease as evidenced by diarrhea is the most common illness in preweaned dairy calves and leads to high rates of mortality and morbidity [28]. Although infections with specific pathogens such as rotavirus, coronavirus, *Cryptosporidium*, *Salmonella*, or *Escherichia coli* may underlie calf diarrhea, the promotion of a healthy microbiota may play a key role in both prevention and treatment. The use of probiotics has emerged as a preventive and alternative therapy to antibiotics and has the potential to reduce the incidence of diarrhea and enhance growth rates in young calves [29, 30]. However, the efficacy of probiotic products is inconsistent across studies [31]. The composition of a calf’s intestinal microbiota contains upwards of 150 genera, which may explain why the performance of limited-species commercial probiotic products vary between animals and farms [32–34] Consequently, the administration of a probiotic inoculum formed from a diverse mixture of microbes may prove superior [35]. FMT utilizing the entire fecal flora may be the most effective probiotic and ultimate therapeutic bacterial mixture [36]. Ultimately, manipulation of the GI microbial community to correct gut dysbiosis can shed light on the role of microbiota in disease, helping to identify specific microorganisms or metabolites responsible for pathophysiologic outcomes [37]. Therefore, we hypothesized that FMT could be used to prevent and treat GI disease in dairy calves by increasing microbial diversity, repopulating the gut biome with beneficial bacteria strains, and altering the environment of the microbial community.

The individualized nature of microbiome compositions is another factor that can contribute to inconsistent results of gut manipulation strategies [38]. Host characteristics are responsible for directly impacting microbial colonization and favor microbes that are already adapted and possess the traits to successfully thrive in the specific ecological conditions of the host [38]. Consequently, introduced microbial strains are more likely to engraft in a new environment if related species are already present, suggesting a role for designing individualized microbial-based products [39]. The concept of individualized medicine incorporates individual genetic and environmental differences to develop novel therapeutic strategies [40, 41]. Therefore, our aim was to developed a farm-specific product that could be produced in a practical manner. The FMT product developed in this study was derived from feces from healthy donor calves (5–24 days of age) raised in the same calf ranch facility and under the same management practices as the FMT recipients. To test the effects of a farm-specific FMT on calves’ health and on their fecal microbiome composition, healthy and diarrheic calves were randomly enrolled to a control or FMT treatment group (~36 g of processed fecal matter administered orally once daily for 3 days), and fecal samples were collected and analyzed at enrollment and again 9 days later.

## Materials and methods

### Ethics statement

The research protocol was reviewed and approved by the Institutional Animal Care and Use Committee of Washington State University (ASAF#6414).

### Study design, calf enrollment

This study was conducted on a commercial calf ranch in the western U.S. between July 14 and August 6, 2019. Consent was written as part of the IACUC protocol (ASAF #6414). The ranch housed approximately 25,000 Holstein, Jersey, and crossbred calves from multiple dairies through 200 days of age. Colostrum was fed to the calves at the dairy of birth, transited <2 hours to the ranch, and arrived at <1 day of age. Five hundred and thirty-seven calves of different breeds (Holstein, Jersey, Jersey-cross [Jersey x Holstein], beef-cross [Jersey x Angus]) arrived at the ranch from July 14–16, 2019. No steers or beef-cross calves were eligible to be enrolled in the study, leaving 385 Holstein, Jersey, or Jersey-cross heifers available for study. Of those, 227 calves from 4–12 days of age were randomly enrolled and assigned to a control or FMT treatment group. Calves assigned to the control group (n = 115) were raised according to farm protocols. In the same manner, FMT calves (n = 112) were raised according to farm protocols, but additionally, FMT was administered orally once daily (~36 g of processed fecal matter) at the time of the morning milk feeding for 3 days in total commencing on the day of enrollment. Fecal samples (~25–250 g) were collected at enrollment (4–12 days of age) and again 9 days later (13–21 days of age). For the purpose of analyses, subgroups 1–9 were defined based on the age at enrollment. Subgroup 1 was sampled at 4 and 13 days of age, subgroup 2 was sampled at 5 and 14 days of age, up to subgroup 9 which was sampled at 12 and 21 days of age. Following this study, calves continued to be housed in the calf ranch and were sold later for production purposes or moved back to the dairy of origin.

### On farm animal care

On-farm personnel were responsible for all primary care of the calves including feeding, cleaning, watering and bedding maintenance. The calves were housed in adjacent individual hutches with no direct contact between calves. Upon arrival, calves were disbudded by on-farm personnel using a caustic paste (Dr. Naylor Dehorning Paste, H.W. Naylor Co. Inc., Morris NY, USA) and sprayed for fly control (Ultra-Boss Pour-on Insecticide, Intervet Inc., Merck Animal Health, Omaha NE, USA). An enteric subcutaneous clostridial vaccine (Ultrabac CD, Zoetis Inc., Kalamazoo MI, USA) and a vitamin complex with selenium and vitamin E (MU-SE, Intervet Inc., Merck Animal Health, Omaha NE, USA) also were administered on arrival. An intranasal viral respiratory vaccine (Vista Once SQ, Intervet Inc., Merck Animal Health, Omaha NE, USA) was administered at 2 days of age and a booster was administered at 20 days of age.

Calves were fed 2 liters of a custom milk blend in a bottle twice daily from 1–15 days of age and 3 liters of the milk blend thereafter until 52 days of age. The milk blend consisted of pasteurized waste milk together with milk replacer and was targeted for an optimal composition of 13% solids, 22–24% fat, and 28% protein. Under the oversight of a licensed veterinarian, milk medicated with neomycin and oxytetracycline (22.2 mg/kg body weight/day, Neo-Oxy 100/100 MR, PharmGate, Omaha NE, USA), was fed to all the calves between 5–12 days of age throughout the study period to combat ongoing GI disease problems. A customized electrolyte also was offered after the afternoon feeding during this same risk period from 5–12 days of age. Fresh water was available between milk feedings. A grain mix consisting of pellets, molasses, and whole corn was offered from 3 days of age and steadily increased to approximately 2.25 kg by day 30 with free choice thereafter.

### FMT inoculum preparation and administration

Consistently clinically healthy calves of different breeds (Holstein, Jersey, Jersey-cross and beef-cross) within 5–24 days of age and with fecal scores ≤2 out of 4 (1 = well-formed; 2 = semi-formed; 3 = loose; 4 = watery) [42] were used as fecal donors to create FMT inoculum. A total of 358 frozen fecal samples collected from 73 calves on the same calf ranch between May 20 and June 19, 2019, were combined to prepare a singular FMT solution [43]. Approximately 5 samples were collected from each donor calf at different days between 5–24 days of age. All fecal samples tested negative for *Salmonella* and were processed under aerobic conditions. Approximately 130 g of mixed samples (range 125.5–134.8 g) were combined in a commercial blender with 650 mL of 0.85% saline. Fecal material was sieved sequentially through 2 meshes (size 35 and 60; Thermo Fisher Scientific, Waltham, Massachusetts, USA) and the slurry was centrifuged (15 min at 6,000 rpm). Supernatant was removed and the gelatinous organic material above the pellet was vacuumed off and discarded. The slurry was re-suspended in a 1:1.25 saline and 10% glycerol solution. Three samples (1 g) of the final FMT slurry were collected for sequencing and analysis of the V3-V4 region of the 16S rRNA following the same protocol used for analyzing the fecal samples from calves. In addition, the FMT product samples were tested by PCR for coronavirus, rotavirus, and *Cryptosporidium* spp. Approximately 6 g of fecal matter solution was allocated into enteric-coated individual size 00 capsules (Capsuline, Dania Beach FL, USA) which were placed within size 0 gelatin capsules (Capsuline, Dania Beach FL, USA) and frozen at -80°C. Capsules were transported to the ranch on dry ice and stored on dry ice that was renewed daily inside a -20°C freezer until administration. During the morning feeding for 3 consecutive days, 6 capsules were administered using a plastic calf bolus gun. Capsules were kept in a cooler with dry ice until their placement within the bolus gun, and a small dab of molasses was added to the tip of the bolus gun to facilitate administration. The total of 36 g of processed fecal material was based on body weight extrapolation from previous FMT studies performed on humans.

### *Salmonella* culture

FMT fecal donor samples were thoroughly mixed and massaged prior to taking two sterile swab samples. Swabs were then inoculated into 9 mL of Rappaport Vassiliadis R10 broth and 9 mL of activated tetrathionate enrichment broth (TET). The swabs were incubated for 18–24 hours at 42°C, then plated onto XLT-4 and incubated for 48 hours at 37°C.

### Bacterial viability in FMT capsules

FMT slurry stored at -80°C was checked for bacterial viability weekly over the course of 3 weeks. Aerobic total bacterial counts were performed via a dilution series of FMT slurry in BHI broth (Brain Heart Infusion broth) and vortexed briefly. One-tenth milliliter from each ten-fold dilution (10^−1^–10^−7^) was spread-plated onto Columbia blood agar plates. Blood agar plates were incubated at 37°C for 18–24 hours and colonies were counted. Anaerobic counts for *Bifidobacterium* were performed using a dilution series of FMT slurry in Wilken Chalgren Anaerobe broth and vortexed briefly. One-tenth from each ten-fold dilution (10^−1^–10^−7^) was spread-plated to transgalactosylated oligosaccharides (TOS) propionated agar media supplemented with 0.05 ug/mL mupirocin. TOS plates were incubated at 37°C under anaerobic conditions (80% N_2_, 10% CO_2_, 10% H_2_) using a 7-liter AnaerobicPack System (Mitsubishi Gas Chemical Company) for 72 hours and colonies were counted. Suspect *Bifidobacterium* was further characterized by identifying the heat shock protein molecular chaperone groEL. This target was used for quantitative polymerase chain reaction (PCR) and to confirm positive bacterial cultures through traditional PCR and gel analysis [44].

### Data collection

#### Clinical assessment score and classification of health status

Two on-farm veterinarians oversaw general calf health management and treatment protocols for the ranch. Diagnoses and treatments were based on input from calf health managers’ clinical assessments. For the purposes of this study, a PhD candidate (GSS) conducted twice daily blinded clinical evaluations based on a standardized calf health-scoring system [45], prior to and at the time of each a.m. and p.m. milk feeding. A WSU veterinarian was responsible for administering the FMT capsules and was not involved in the clinical assessments. Assessments began on the day of arrival through the completion of the follow-up period to 21 days of age. Primary points of variation included demeanor (bright/alert/responsive vs depressed/dull), milk intake (good appetite; did not finish the milk or did not consume any of what was offered; orogastric intubation), hydration status (mm of ocular recession), and fecal consistency scores [42]. Due to inconsistencies in fecal appearance within the calf hutches and an inability to observe each calf defecate at the time of all clinical evaluations, fecal consistency scores only were recorded at the time of fecal sampling. Calves with fecal scores of 3 or 4 were diagnosed with diarrhea. Calves with systemic GI disease were treated medically by farm personnel based on farm protocols incorporating the following alone or in combination: IV fluid therapy (lactated Ringer’s solution), anti-inflammatories (flunixin meglumine or dexamethasone), or variable IM or SQ antibiotics (ceftiofur crystalline free acid, florfenicol, or oxytetracycline). Calves with fecal scores <3 at the time of sampling were classified as consistently healthy if they neither demonstrated abnormal clinical parameters or behavioral scores during a period of 21 days after arrival, nor received any treatment for GI disease, respiratory disease, or other health problems during the sampling period.

#### Fecal samples

Fecal samples were collected after the morning feeding in sterile sampling bags (Thermo Fisher Scientific, USA) and immediately placed in a cooler with ice packs until they could be frozen (–20°C) within five hours after sampling. The samples were transferred to Washington State University on dry ice and stored for approximately 30 days in a –20°C freezer until further processing within the Field Disease Investigation Unit laboratory.

#### Fecal dry matter

Fecal samples were assessed for total dry matter by weighing out 1 g of raw sample and drying the sample in an incubator at 37°C for 72 hours to remove moisture. Percent dry matter was calculated based on the difference between dry weight and wet weight.

### Amplification and sequencing of bacterial 16S rRNA gene

At the time of processing, fecal samples were thawed, mixed, and placed (1 g) into fecal DNA/RNA shield fecal collection tubes (Zymo Research, Irvine, CA). DNA extraction and amplification of the V3–V4 region (primers 341F-806R) of the 16S rRNA gene was performed by Zymo Research (Irvine, CA).

### Statistical analysis

Fastq files generated by Illumina MiSeq (2x300bp) amplicon reads were processed using the package dada2 [46] in R program 4.0.0 (R Project for Statistical Computing). Unique sequences were classified to species levels by SILVA 123 ribosomal RNA database [47] using SPINGO [48]. If sequences were identified as ambiguous by SPINGO, Ribosomal Database Project 11, University of Illinois in Urbana, IL [49], was used for taxonomy identification, and if sequences were still undefined, the next approach adopted was Basic Local Alignment Search Tool (BLAST) [50] available from the National Center for Biotechnology Information [51].

The final amplicon sequence variant (ASV) table was filtered via mutual information-based microbiome analysis that accounted for information loss [52]. Alpha-diversity was analyzed using the diversity function from the vegan R package based on Shannon index [53]. A linear mixed effects model was fitted using the lmer function of lme4 package with the objective to identify differences in diversity between treatments groups. The primary response variable of interest was the Shannon index and the explanatory variables included treatment group, clinical outcomes (healthy or diarrheic), breed, and sampling subgroup. In addition, the calf ID was considered as a random effect in the model. A Venn diagram was calculated using the venn function of the eulerr package in R [54]. To explore and visualize dissimilarities between groups, Beta-diversity was analyzed using the ordinate function in R’s phyloseq package [55]. We created a Principal Coordinates Analysis (PCoA  =  Multidimensional scaling, MDS) using the normalized number of reads in each sample based on the total number of reads per sample and the modified Gower distance (altGower) [56]. Differences in community composition were tested statistically with permutational analysis of variance (PERMANOVA) using the adonis function of the vegan package [53]. In order to identify differences across treatment groups the function pairwise.adonis of the pairwiseAdonis R package was used. The relative abundance heatmap was calculated based on the number of sequence reads normalized by the number of total reads per sample using the amp_heatmap function of the ampvis2 R package [57].

A generalized linear model was fit using the glm function of stats package [58] based on the log difference of read counts for each animal between the first (day 0) and second sampling day (day 10) for organisms of interest. The log difference between the total read counts of samples collected at day 0 and day 10 for each animal was used as an offset term. This analysis had the objective to identify the relationship between specific microbial organisms and FMT treatment. The response variable was the read counts log differences between the two sampling times for a specific organism for each animal. The primary explanatory variable of interest was treatment group (FMT and control). Other variables included the following: 1) clinical outcomes on the day of enrollment (day 0 healthy or diarrheic), to control for differences in FMT engraftment based on clinical status at the time of product administration; 2) subgroup, to control for age difference of calves in each sampling group, 3) breed, and 4) an interaction between treatment and the animal health state at enrollment. To analyze the impacts of FMT treatment on GI disease incidence we used a binomial family to fit a generalized linear regression model [59]. The response variable was the health status at the second sampling time (day 10). The explanatory variables were the health status of the calf at the day of enrollment and treatment group (FMT and control). The predict function in R’s stats package was used to predict the chances of a calf having diarrhea at day 10 across treatment groups based on the generalized model results. R code and its output can be found in S1 Appendix.

### Sequence availability

The fecal microbiome sequences have been deposited in the NCBI database under BioProject accession number PRJNA752434 under the Sequence Read Archive (SRA) accession IDs of SAMN20595420- SAMN20595483 and under the BioProject accession number PRJNA792140 under the SRA accession IDs of SAMN24386562 –SAMN24386741.

## Results

### FMT composition

**Fig 1:**
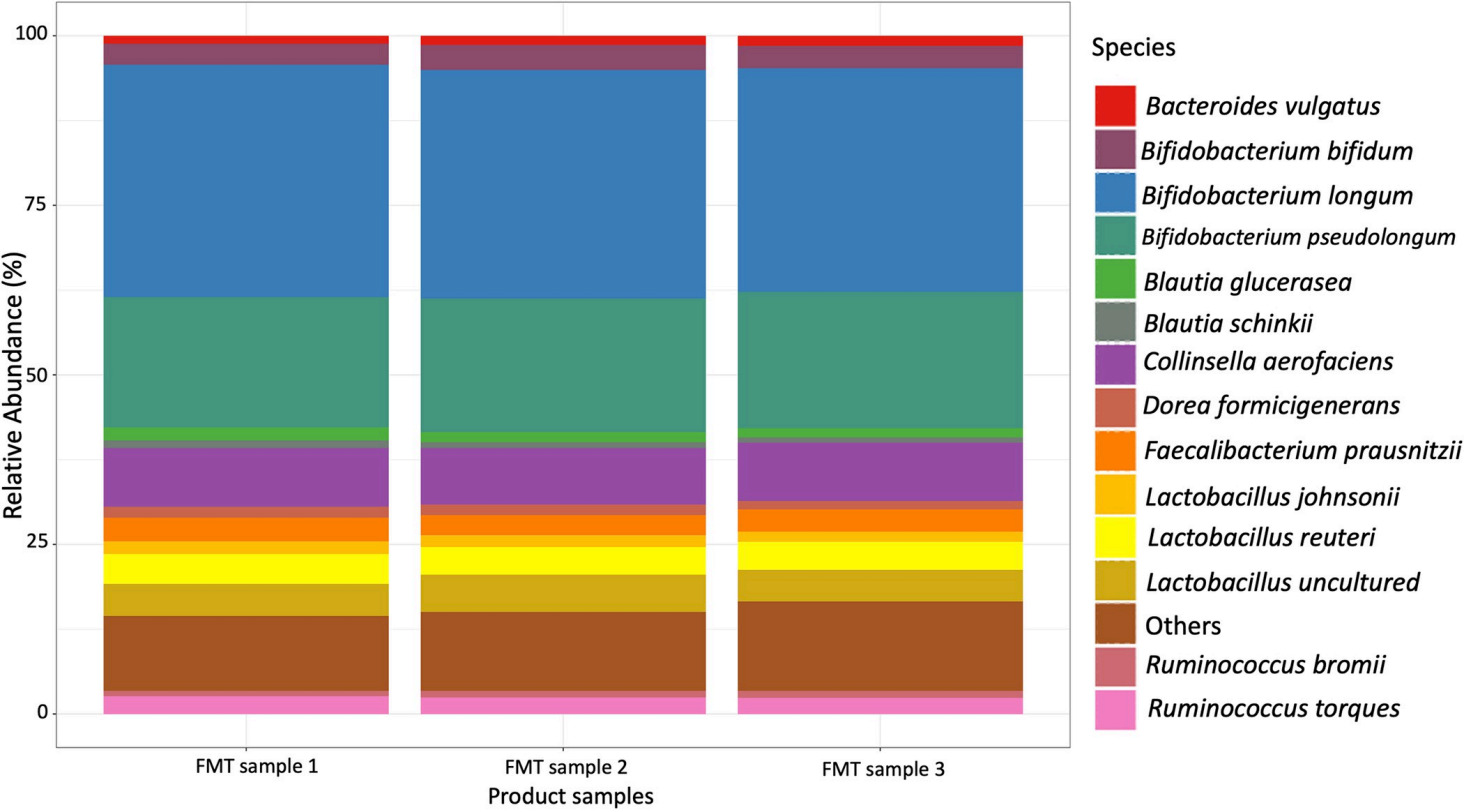
Relative abundance of three FMT product samples at species level.

Three samples (1 g) of the final FMT product were collected and the V3-V4 region of the 16S rRNA gene was sequenced. The average microbial composition of the FMT product samples at the phylum level was 57% Actinobacteria, 38% Firmicutes, 3.5% Bacteroidetes, and 1% Proteobacteria (S1 Appendix). The FMT product had an enrichment of organisms of the genera *Bifidobacterium*, *Blautia*, *Collinsella*, *Fecalibacterium*, and *Lactobacillus* (Fig 1). Furthermore, the FMT product was positive for coronavirus, rotavirus, and *Cryptosporidium* spp. when analyzed by PCR.

The FMT capsule contents were tested to assess bacterial viability at 1-, 2-, and 3-weeks post manufacturing to represent the length of time the capsules were stored prior to administration. The anaerobic results were based on *Bifidobacterium* viability, whereas the aerobic test detected facultative anaerobes that were counted but not differentiated. The bacterial viability test showed modest reductions over time (Table 1).

**Table 1:** Bacteria viability of FMT capsule content over the course of 3 weeks of storage.

	Aerobic	Anerobic
**Week 1**	2.33x10^9^	1.57x10^10^
**Week 2**	1.27x10^9^	1.15x10^10^
**Week 3 on farm**	1.03x10^9^	6.81x10^9^

### Calves enrolled in the study, FMT administration, and GI disease incidence

Of the 385 eligible calves that arrived on the calf ranch during the enrollment period, 227 were enrolled in the study. Of those, 112 were randomly allocated to the FMT treatment group and 115 to the control group (Table 2). Numerically, systemic GI disease incidence was higher in the FMT group compared with the control group (29 vs. 7 calves respectively). Of the 29 FMT calves treated for GI disease, 10 were Holstein, 16 Jersey, and 3 Jersey-cross calves. Of the 7 calves treated for GI disease in the control group 3 were Holstein and 4 were Jersey calves. The overall number of calves treated for respiratory problems (n = 9), GI disease in tandem with respiratory symptoms (n = 4), and other reasons (joint problems, septic; n = 7) was less than for those treated for GI disease (n = 102). Moreover, calves were more likely to die if they received the FMT product (Chi-square p<0.001). A total of 12 calves died due to GI disease in the FMT group (Holstein n = 3, Jersey n = 8, and Jersey-cross n = 1) ranging between 1–13 days after the first dose of FMT administration. Only one Holstein calf died because of GI disease complications in the control group. Further details regarding calves that died during the study can be found within S1 Table. Additionally, Table 2 displays the breed distribution, total serum protein, treatment and mortality levels for calves that arrived on the ranch during the same period that were eligible but not enrolled in the study.

**Table 2:** Number of calves enrolled by breed and treatment group, TSP measurements, GI disease treatment, and mortality levels.

	FMT	Control	Calves not enrolled, but eligible to be enrolled
**Number of enrolled calves**	112	115	158
**Holstein calves enrolled**	48 (43%)	51 (44%)	117 (74%)
**Jersey calves enrolled**	46 (41%)	47 (41%)	13 (8%)
**Jersey-cross calves enrolled**	18 (16%)	17 (15%)	28 (18%)
**Median TSP (g/dL) and IQR**	6.5 ±0.8	6.3 ±0.8	6.1 ±0.8
**Number of calves treated for systemic GI disease**	29 (26%)	7 (6%)	66 (42%)
**Number of calves treated for respiratory disease**	3 (3%)	2 (2%)	4 (3%)
**Number of calves treated for other problems**	3 (3%)	3 (3%)	1 (1%)
**Number of calves that died due to systemic GI disease**	12 (11%)	1 (1%)	4 (3%)
**Number of calves that died due to systemic GI and respiratory disease**	3 (3%)	0	1 (1%)
**Number of calves that died due to respiratory disease**	2 (2%)	0	0
**Number of calves that died due to other reasons**	2 (2%)	0	0
**Total number of calves that died**	19 (17%)	1 (1%)	5 (3%)

TSP = total serum protein.

IQR = interquartile range.

### Fecal microbiota assessment

A subset of enrolled calves that survived the 21-day study period and were not treated with additional antibiotics or anti-inflammatories were selected for fecal microbiome analysis to assess the impacts of FMT on their fecal microbial composition. Table 3 demonstrates the breed and disease progression for calves selected for microbiome analysis. Overall, 242 fecal samples collected at enrollment (average age 8 days; range 4–12 d) and 9 days later (average age 17 days; range 13–21 d) were analyzed from 121 calves. Of those, 59 calves received FMT and 62 did not. A total of 170 fecal samples were collected from healthy calves at the time of sampling, and 72 were from calves with diarrhea at the time of sampling. Diarrheic samples analyzed in this study were from uncomplicated cases of GI disease, i.e., calves did not have any other systemic clinical signs of GI disease besides loose feces (fecal score 3 or 4). Calves with systemic clinical signs of GI disease required antibiotic therapy and those samples were removed from the analysis. The sample distribution per age, breed, treatment and health state can be found in S2 Table. The average fecal dry weight (median and interquartile range, IQR) of samples from healthy calves was 27.8% (28%, IQR 9.1%) and samples from diarrheic calves averaged 14.3% (13.1%, IQR 9.9%). In addition, the average fecal dry weight of the 3 FMT product samples was 32%.

**Table 3:** Number of calves sampled by treatment, breed, and disease progression.

Treatment group	Disease progression	Holstein	Jersey	Jersey-Cross
**Control**	Healthy-Healthy	9	11	6
	Healthy-Diarrhea	6	3	1
	Diarrhea-Healthy	6	13	6
	Diarrhea-Diarrhea	0	1	0
**FMT**	Healthy-Healthy	12	12	4
	Healthy-Diarrhea	6	7	1
	Diarrhea-Healthy	5	5	3
	Diarrhea-Diarrhea	1	1	2

Results from the logistic regression model indicated that control calves diagnosed with diarrhea at enrollment were less likely to have diarrhea at day 10 (p = 0.03). In addition, a healthy calf receiving FMT had a 33.3% chance of having diarrhea at day 10, compared with a 27.7% chance of a healthy calf developing diarrhea when enrolled in the control group. FMT calves diagnosed with diarrhea at enrollment had a 23.5% chance of having diarrhea at day 10. Control calves diagnosed with diarrhea at enrollment had only a 3.8% chance of having diarrhea at day 10.

A total of 1,918 ASVs were observed and 373 species were identified after taxonomy assignment across the 242 fecal samples. We first determined an appropriate threshold for mutual information scores in our microbial network using an expected power-law distribution; the best-fit power-law distribution (R^2^ = 0.85) occurred when mutual information scores (network edges) below 0.2 were dropped. After dropping these edges from the network, we determined the number of ASVs to retain for analysis by sequentially dropping ASVs from lowest abundance to highest abundance, and checking afterward if the information loss in the system was significant [52]. This process indicated that we could remove 81% of the sequencing data without significant information loss (p > 0.05 following Benjamini-Hochberg correction). These methods resulted in 81 “significant” ASVs which were included in subsequent analyses; the remaining 292 “insignificant” ASVs were grouped into an “other” category.

**Fig 2:**
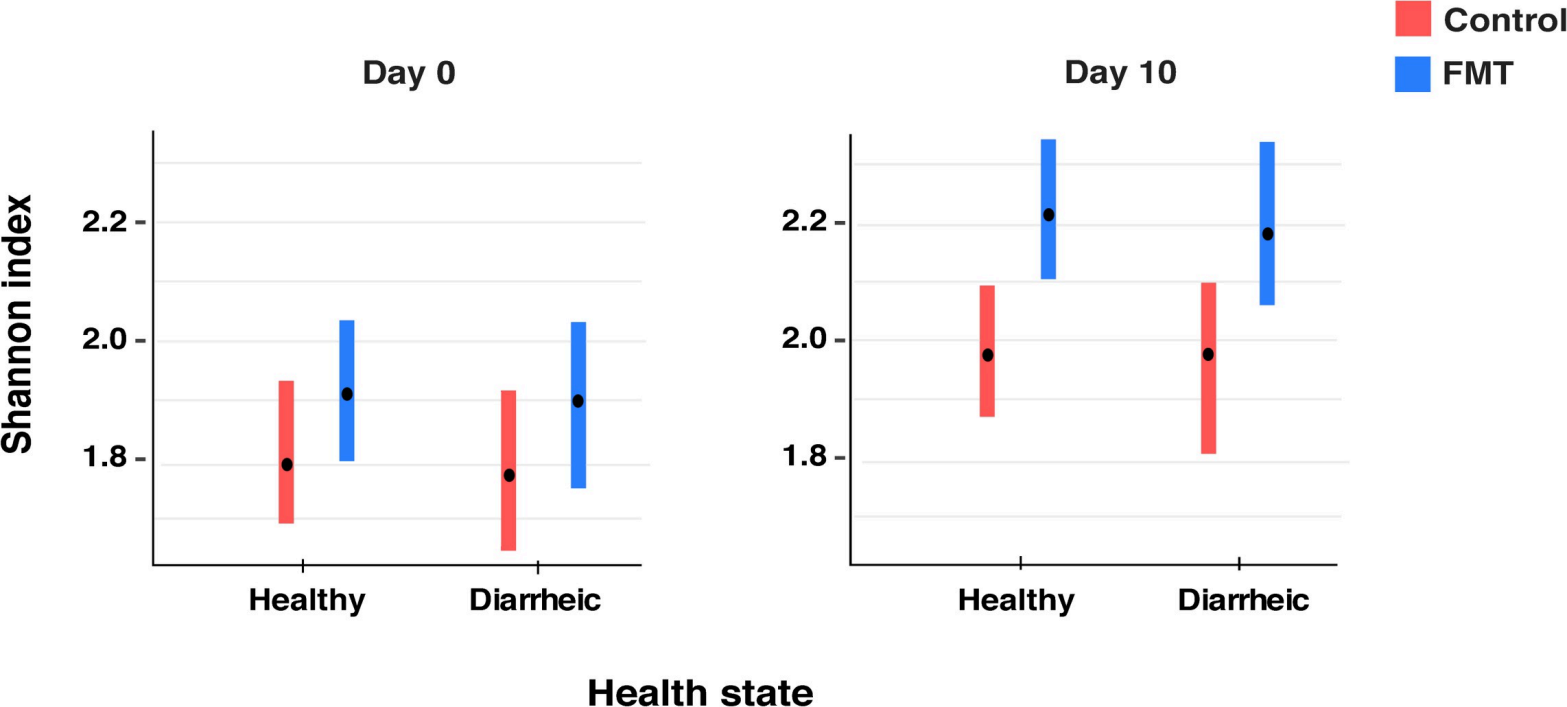
Alpha-diversity analysis using Shannon index. Samples were grouped by treatment and health status. Day 0 reflects the day of enrollment when calves were first sampled and FMT calves received their first dose of FMT. Day 10 reflects the second sampling time, 9 days after enrollment of both groups. The bars indicate the 95% confidence intervals.

Bacterial diversity was estimated using the Shannon index across treatment groups for both the first and second sampling time as well as for different health states (Fig 2). The results of the linear mixed effects model showed that FMT calves had a higher diversity based on Shannon index compared with calves that did not receive FMT (p = 0.005). Moreover, samples collected from control calves on day 10 had a higher diversity compared with samples collected at enrollment regardless of disease state (p = 0.01), indicating age related changes. We did not observe differences in the Shannon index between healthy and diarrheic calves. Moreover, we did not observe any differences across breeds or sampling groups (S1 Appendix). The Shannon index results for the three FMT product samples were 2.4, 2.5 and 2.5, respectively.

**Fig 3:**
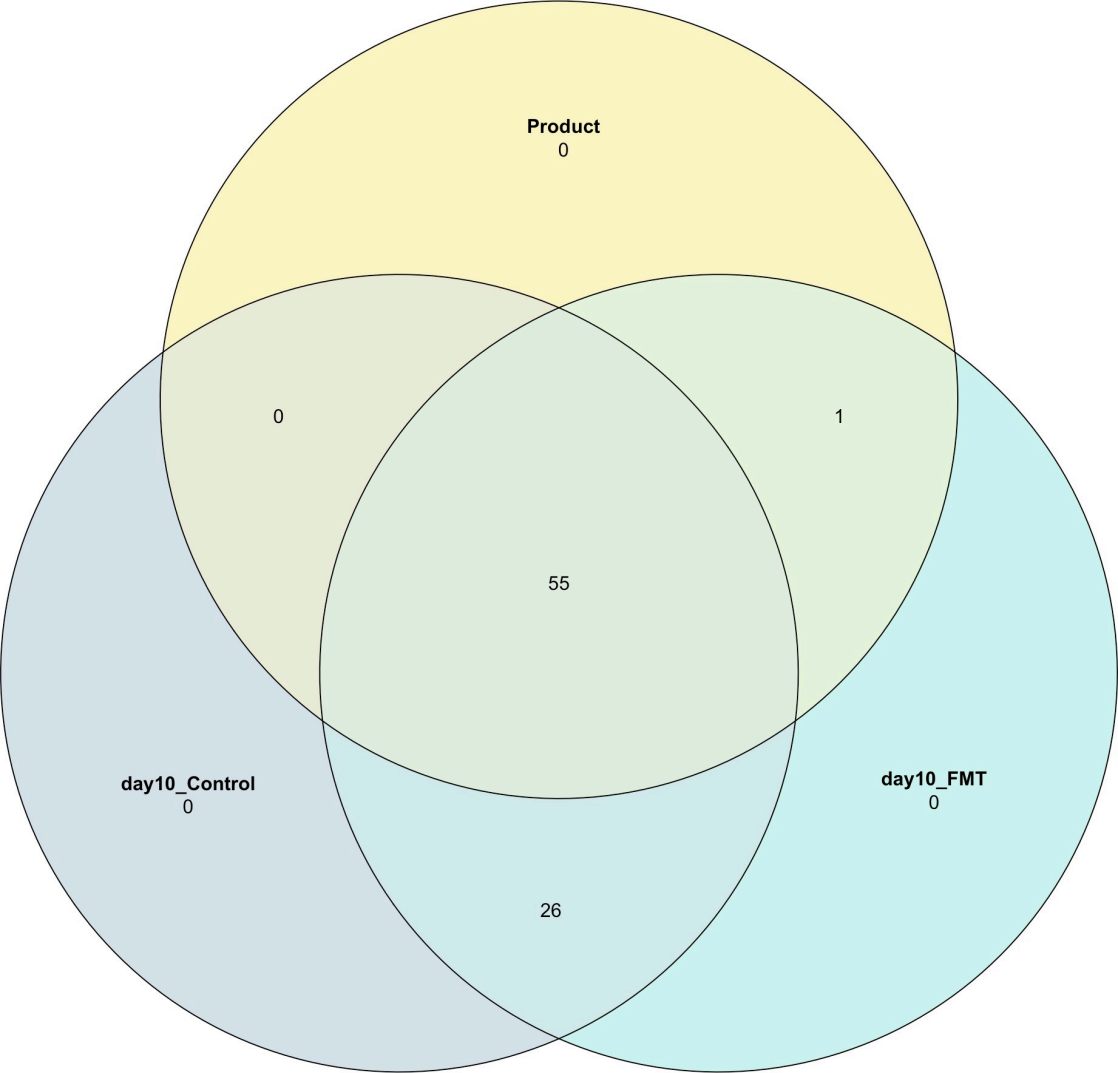
Venn diagram to assess species that were shared between calves that did or did not receive FMT. The different colors represent the different samples: FMT product, day 10 samples from calves that received FMT previously (day10_FMT), and samples from calves that did not receive FMT (day10_Control).

A Venn diagram was calculated to assess species shared between the FMT product and day 10 samples from calves that did or did not receive the FMT product (Fig 3). A total of 55 species were shared between the FMT product and the fecal samples from calves that did or did not receive FMT. However, 26 species were only shared between the fecal samples from calves of both groups and not with the product. Within the 81 ASVs that were kept in our dataset and the combined category of ASVs (“other”), only *Oribacterium sinus* was shared exclusively between the product and calves that received the FMT.

**Fig 4:**
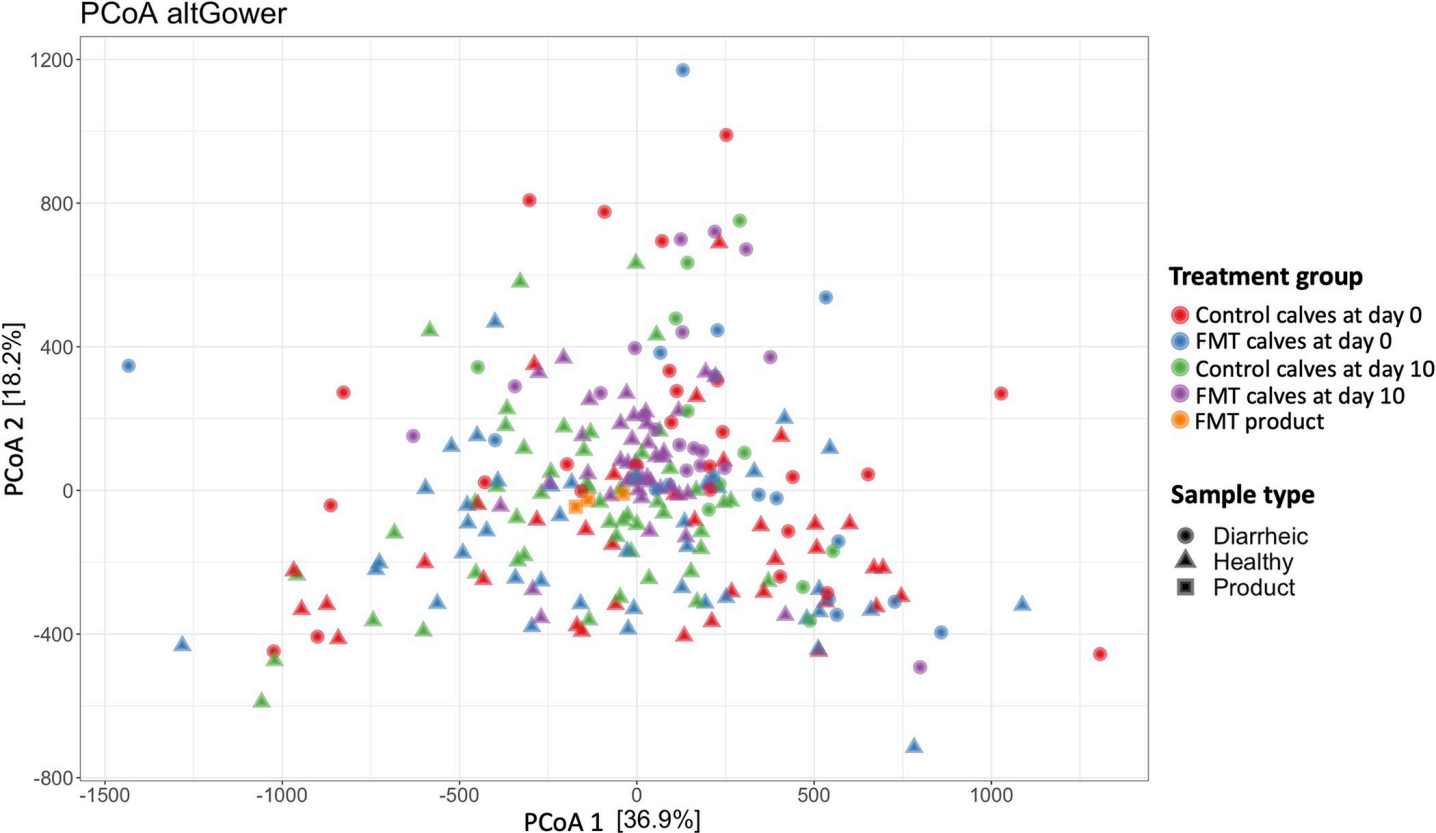
Principal coordinates analysis (PCoA) based on Gower distances grouped by treatment group and health status. Proportion of variance explained by each principal coordinate axis is denoted in the corresponding axis label. Samples were grouped by treatment groups. The different shapes indicate the calf health status at the time of sampling.

Principal coordinate analysis (PCoA) based on Gower distances was performed to explore differences in the fecal microbial composition before and after the FMT administration, as well as between calves that did or did not receive FMT. Surprisingly, the results of the beta diversity analysis did not reveal a clear separation between FMT and control calves (Fig 4). PERMANOVA results indicated a difference between samples from healthy and diarrheic calves (p-value = 0.001, from 999 permutations of the analysis of variance; PERMANOVA), but no difference in the fecal bacterial community’s organization was observed between treatment groups. However, pairwise PERMANOVA showed a tendency for samples collected at day 10 to differ across treatment groups (control vs. FMT; p-value = 0.06).

**Fig 5:**
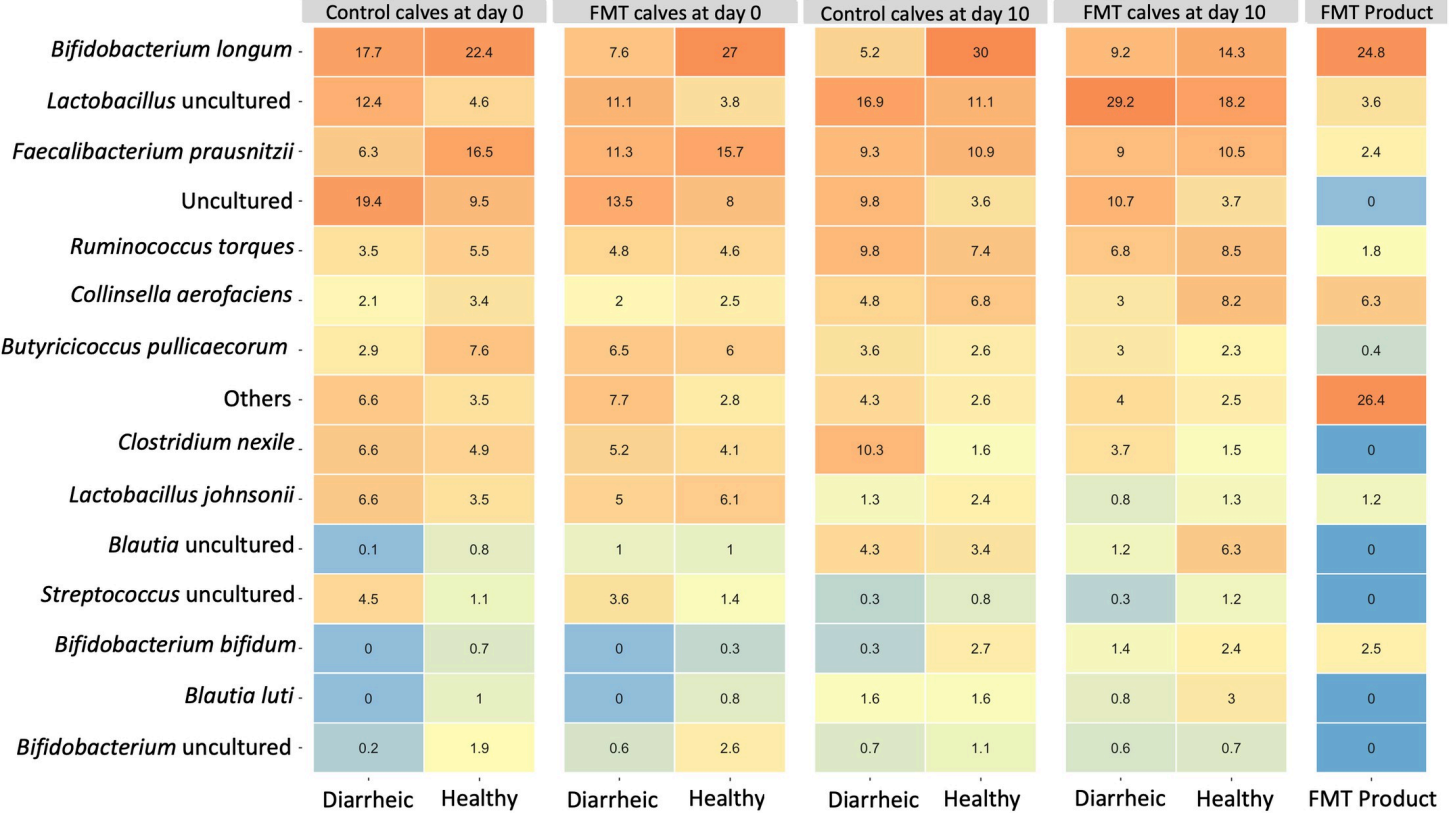
Relative abundance heatmap. The relative abundance of each species across groups is indicated by the values in the tiles. The color gradient indicates different levels of relative abundance.

A heatmap with the percentage of the empirical means of the relative abundance at the species level was calculated for the FMT product and all calves across different groups during the first and second sampling period (Fig 5). Results of the generalized linear models at the species level showed that FMT calves had a higher relative abundance of uncultured organisms of the genus *Lactobacillus* (p = 0.0005) and *Lactobacillus reuteri* (p<0.001) on day 10, as compared to control calves during the same period. Moreover, FMT calves had lower relative abundance of *Clostridium nexile* (p<0.001) and *Bacteroides vulgatus* (p = 0.05) on day 10 as compared with control calves. Calves sampled at 4 and 13 days of age (subgroup 1) had an enrichment of *Bifidobacterium longum* compared with calves sampled in the other subgroups (see S1 Appendix for the p-values for each subgroup). Control calves enrolled with diarrhea had an enrichment of *Bifidobacterium longum* on day 10 (p = 0.01) as compared with control calves enrolled as healthy. Jersey calves had higher relative abundance of *Bifidobacterium bifidum* (p = 0.03) as compared with Holstein calves. In addition, FMT calves with diarrhea at enrollment had higher relative abundance of *Butyricicoccus pullicaecorum* (p = 0.03) and *Blautia luti* (p = 0.05), and lower relative abundance of an uncultured organism of the genus *Clostridium* (p = 0.01) on day 10 as compared with control calves enrolled as healthy.

## Discussion

This study evaluated the capacity of FMT product derived from healthy donor calves to prevent or treat GI disease and alter the fecal microbial composition in preweaned dairy calves. Although the FMT product was rich in organisms of the genus *Bifidobacterium*, *Lactobacillus*, and *Fecalibacterium* (Fig 1, Table 1) typically known for their beneficial probiotic properties [11, 60], the FMT therapy did not prevent or ameliorate GI disease in dairy calves. Calves that received FMT were less likely to recover from GI disease, and more likely to die due to GI disease complications (Table 2, S1 Table). One limitation of our product was that we did not exclude infectious pathogens aside from *Salmonella*. Our FMT product was positive for coronavirus, rotavirus, and *Cryptosporidium* spp. when analyzed by PCR. Previous studies that reported positive effects of FMT therapy in calves and piglets targeting diarrhea did an extensive screening for common pathogens before manufacturing the product [23, 61, 62]. In this study, we were aiming to develop a farm-specific product that could be produced in a practical manner. Because pathogens were present in fecal samples of healthy calves on this specific calf ranch, we did not restrict our product to fecal samples from donors free of pathogens. Therefore, we might have delivered an infectious dose of viral or protozoal pathogens during a high-risk period of disease, which might have contributed to the higher incidence of GI disease and mortality levels as well as the low recovery rates in calves that received FMT. However, the higher number of treatments targeting systemic GI disease observed in the group of calves that were eligible to be enrolled but were not part of the study remains unexplained.

Shannon index was used to estimate alpha-diversity across groups (Fig 2). Increased diversity of gut microbiota has been reported to be a feature of healthy (non-diarrheic) calves during early life [11, 63]. Interestingly, we did not observe any differences in diversity in the fecal microbiome between samples from healthy calves and calves with uncomplicated GI disease. However, calves that received FMT had a higher diversity at day 10 based on Shannon index compared with calves that did not receive FMT (p = 0.0005). Similarly, a recent study reported that dairy calves that received 25 g of FMT mixed in the milk replacer from 8–12 days of age showed an increase in diversity [64]. Within the 81 ASVs that were kept in our dataset, we identified only one specific organism (*Oribacterium sinus*) that was shared exclusively between the FMT product and calves that received FMT (Fig 3). This observation suggests that the product did not contribute to the richness of the calf fecal microbiome. Instead, this implies that the product influenced the abundance of species already present within the fecal microbiome. Given this scenario, the FMT might also have contributed to an increase in the evenness of species that are involved in GI disease and influenced the increased GI disease incidence in the FMT group. Although the mechanisms of FMT therapy are not fully understood, normalizing the microbial diversity and community structure has been described as an important feature when targeting GI disorders [65]. In our study, the increased fecal microbial diversity observed in FMT calves was not conductive of better gastrointestinal health outcomes. In fact, the FMT product seemed to have disrupted the gut rather than contributed to a restoration of a healthy microbial community ecology. Our findings highlight the importance of performing a rigorous donor screening to administer FMT safely.

Although calves that received FMT had a higher alpha-diversity, no substantial differences in the fecal bacterial community’s structure were observed between treatment groups (p = 0.06 pairwise PERMANOVA; Fig 4). We did not use a specific method to assess the extent and duration of bacterial engraftment; however, a recent study observed the highest proportion of ASVs shared between FMT donor and FMT recipient beef-calves 16 days after two oral FMT treatments (40 mL; 0.0004 g/mL feces) [23]. In our study, no major differences were observed between the FMT product samples and fecal samples from calves in the control and FMT groups. It has been suggested that the gut microbiota of a patient after FMT administration is distinct from the donor/product and the patient before the FMT, and that specific bacteria are more likely to engraft based on abundance and phylogeny of bacteria in the donor and patient before FMT [66]. Although we did not observe differences in the community structure of calves before and after FMT treatment, calves in the FMT group that were enrolled with diarrhea had an enrichment of *Butyricicoccus pullicaecorum* and *Blautia luti*, and lower relative abundance of an uncultured organism of the genus *Clostridium* on day 10 following their first dose of FMT. This suggested that the health status of the patient at the time of FMT administration might influence fecal microbial outcomes.

Members of the genus *Lactobacillus* are known to contribute to gut health and exert antibacterial activities against pathogenic bacteria that commonly induce diarrhea in cattle [67]. Oral administration of one daily dose for 10 consecutive days of either *Lactobacillus reuteri* or *Lactobacillus johnsonii* has been shown to decrease diarrhea incidence in dairy calves [68]. Interestingly, FMT calves had a higher relative abundance of an uncultured organism of the genus *Lactobacillus* and *Lactobacillus reuteri* on day 10 (Fig 5). A higher relative abundance of *Lactobacillus* organisms had been observed previously in calves with GI disease on the same ranch [43].This pattern within the microbial community suggested that the FMT might have disrupted the microbiome, altering the fecal microbial composition and gut ecology in such a way as to be conducive for disease. Overall, FMT administration failed to prevent or treat GI disease in our study and was associated with an increase in the number of calves that died. Previous studies that successfully administered FMT targeting GI disease in young animals used fecal samples from older animals (21–50 days of age) or adult donors [23, 61, 62, 64]. Given that during maturation the GI tract undergoes several morphological and functional adaptations early in life [69], we chose to use fecal samples from donor calves roughly the same age as the FMT recipients. Our intent was to increase the likelihood that the microbes would thrive in an age-adapted environment. That said, the fecal microbiome of calves during the first weeks of life has been reported to be diverse and rapidly changing [8, 70]. The instability of the microbial community characteristic at this young age might have contributed to the failure of the FMT therapy in our study.

Contrary to our assumption, the FMT treatment was clinically inferior to the control treatment in terms of GI disease development and recovery. The recommendations when developing a FMT product are to collect samples from individuals with no history of disease or history of antibiotic treatment [71, 72]. Because fecal samples from donor calves were collected at the same ranch facility where this study was conducted, donor calves sampled during 5–12 days of age were receiving milk medicated with neomycin and oxytetracycline. Moreover, calves in this present study were also receiving medicated milk with neomycin and oxytetracycline during the same age period. The combination of enteral antibiotic treatment (neomycin and amoxicillin-clavulanate) with rectal FMT has been reported to be detrimental to neonatal piglets [73]. Moreover, antibiotic use within the first 8 weeks after FMT transplantation may disrupt microbial engraftment and limit FMT effectiveness in patients with *Clostridium difficile* infection [74]. Further investigations into how route of administration, dosage, duration of treatment, and antibiotic exposures influence the efficacy of FMT therapy in dairy calves are needed to ensure patient safety and the development of safer microbiota-based treatments. Taken together, our results indicate the need to have an established protocol when developing FMT products, based on rigorous inclusion and exclusion criteria for the selection of FMT donors free of potential pathogens. This is especially true when administering FMT to young animals with an unstable fecal microbial community [8] and naive immune system [75].

## Supporting information

S1 Appendix R Markdown file (pdf) with R code and its output. (PDF)

S1 Table Details about calves that died during the study period. List of calves that died by treatment group, breed, on-farm diagnosis and days post FMT therapy.
(XLSX)

S2 Table Samples distribution by age, breed, health states, and treatment group. (XLSX)
