# Supplementary material for: Effects of a farm-specific fecal microbial transplant (FMT) product on clinical outcomes and fecal microbiome composition in preweaned dairy calves
Source: PLoS One. 2022 Oct 21;17(10):e0276638. doi: 10.1371/journal.pone.0276638 (PMC9586405; doi:10.1371/journal.pone.0276638)

dada2

Giovana Slanzon

12/10/2021

```
library("dada2")

## Loading required package: Rcpp

## Warning: package 'Rcpp' was built under R version 4.0.2

## Warning: multiple methods tables found for 'which'

library("ggplot2")

## Warning: package 'ggplot2' was built under R version 4.0.2

fqs <- dir() #list all of the files in the working directory, and save as fqs

fqs <- fqs[grep(".fastq.gz$",fqs)] #remove any file from fqs that isn't a "*.fastq.gz"
file


fqs.for <- fqs[grep("_R1",fqs)] #forward group of fastq files, grep looks for the patt
ern "_R1"

fqs.rev <- fqs[grep("_R2",fqs)] #reverse group of fastq files, grep looks for the patt
ern "_R2"


sample.names <- sapply(strsplit(fqs.for, "_"), `[`, 2)

#Let's output some plots from sample 1's reads

plotQualityProfile(fqs.for[[1]]) + ggtitle("Quality Scores for Forward Reads, FMT Part
II")

## Warning: `guides(<scale> = FALSE)` is deprecated. Please use `guides(<scale> =

## "none")` instead.
```

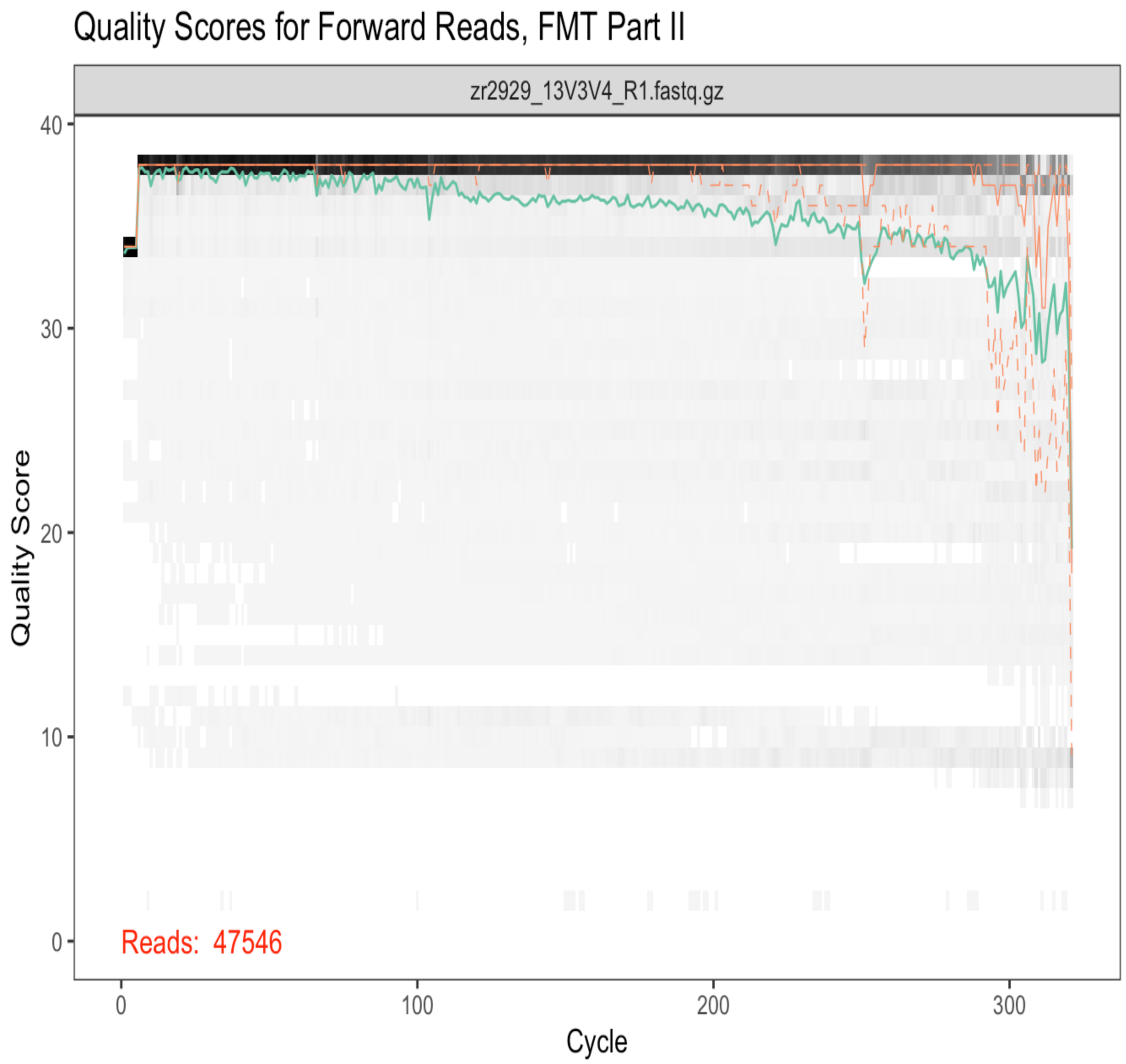


```
plotQualityProfile(fqs.rev[[1]]) + ggtitle("Quality Scores for Reverse Reads, FMT Part
II")

## Warning: `guides(<scale> = FALSE)` is deprecated. Please use `guides(<scale> =
## "none")` instead.
```

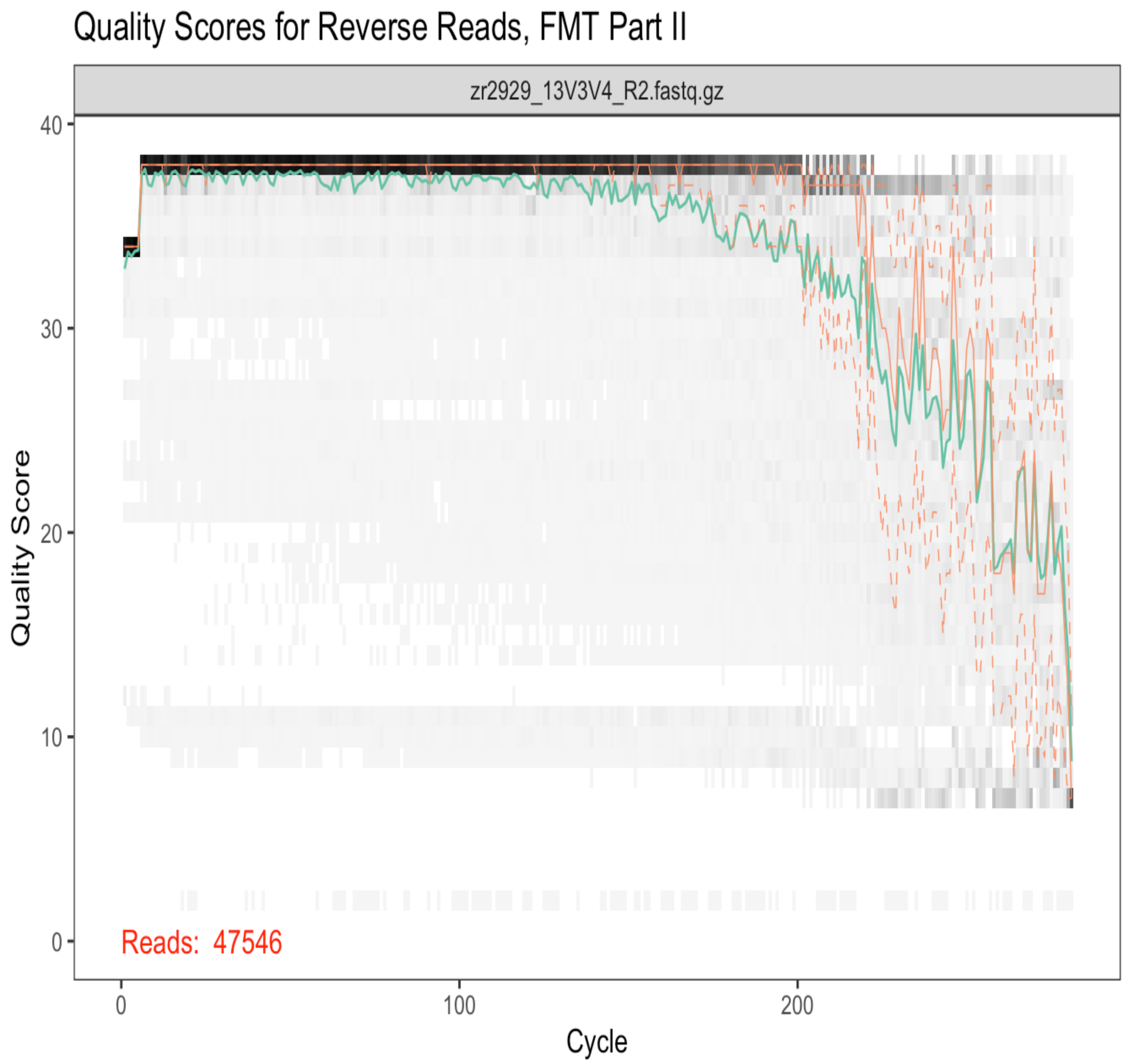


```
plotQualityProfile(fqs.for[[2]]) + ggtitle("Quality Scores for Forward Reads, FMT Part
II")

## Warning: `guides(<scale> = FALSE)` is deprecated. Please use `guides(<scale> =
## "none")` instead.
```

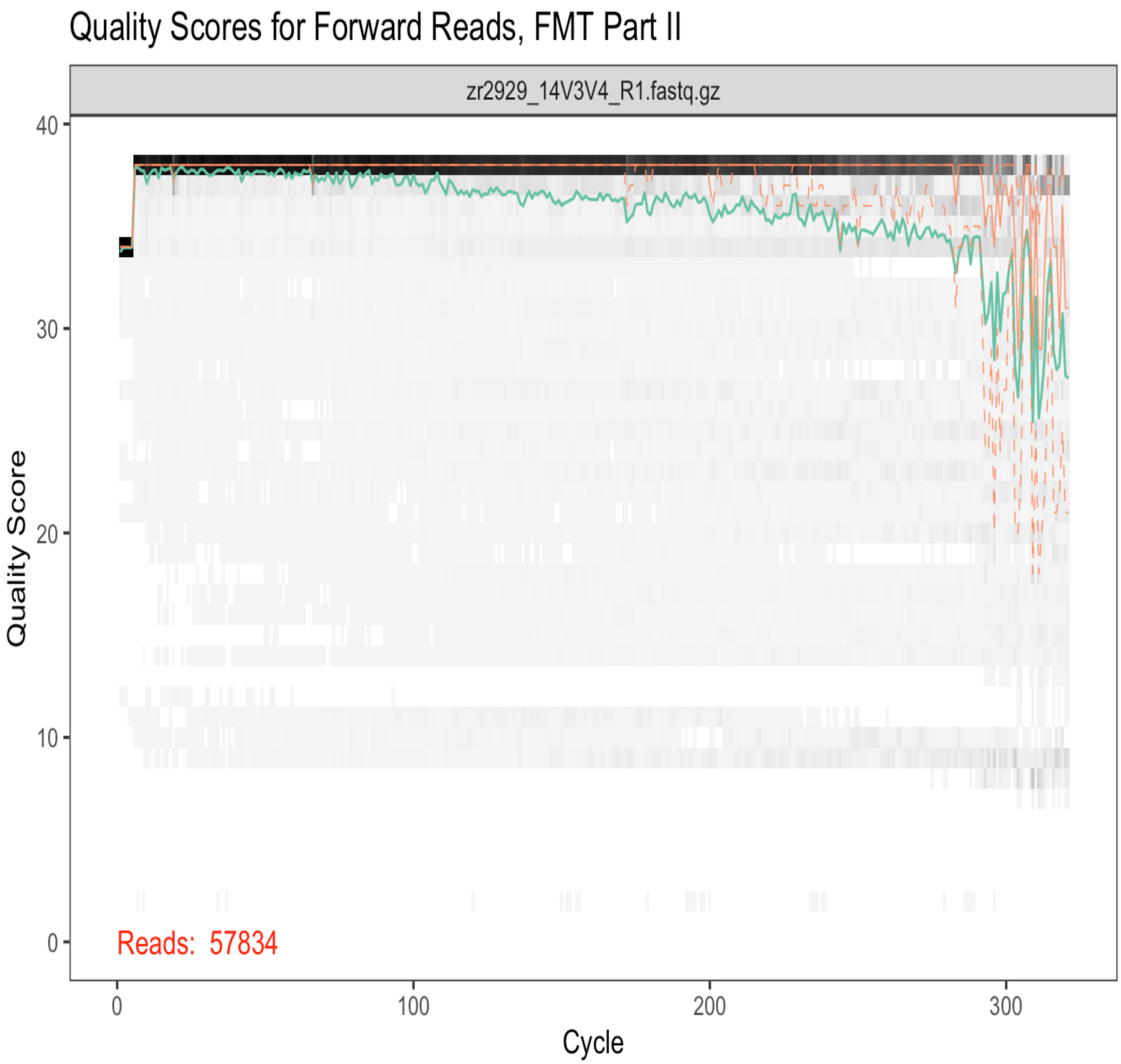


```
plotQualityProfile(fqs.rev[[2]]) + ggtitle("Quality Scores for Reverse Reads, FMT Part
II")

## Warning: `guides(<scale> = FALSE)` is deprecated. Please use `guides(<scale> =
## "none")` instead.
```

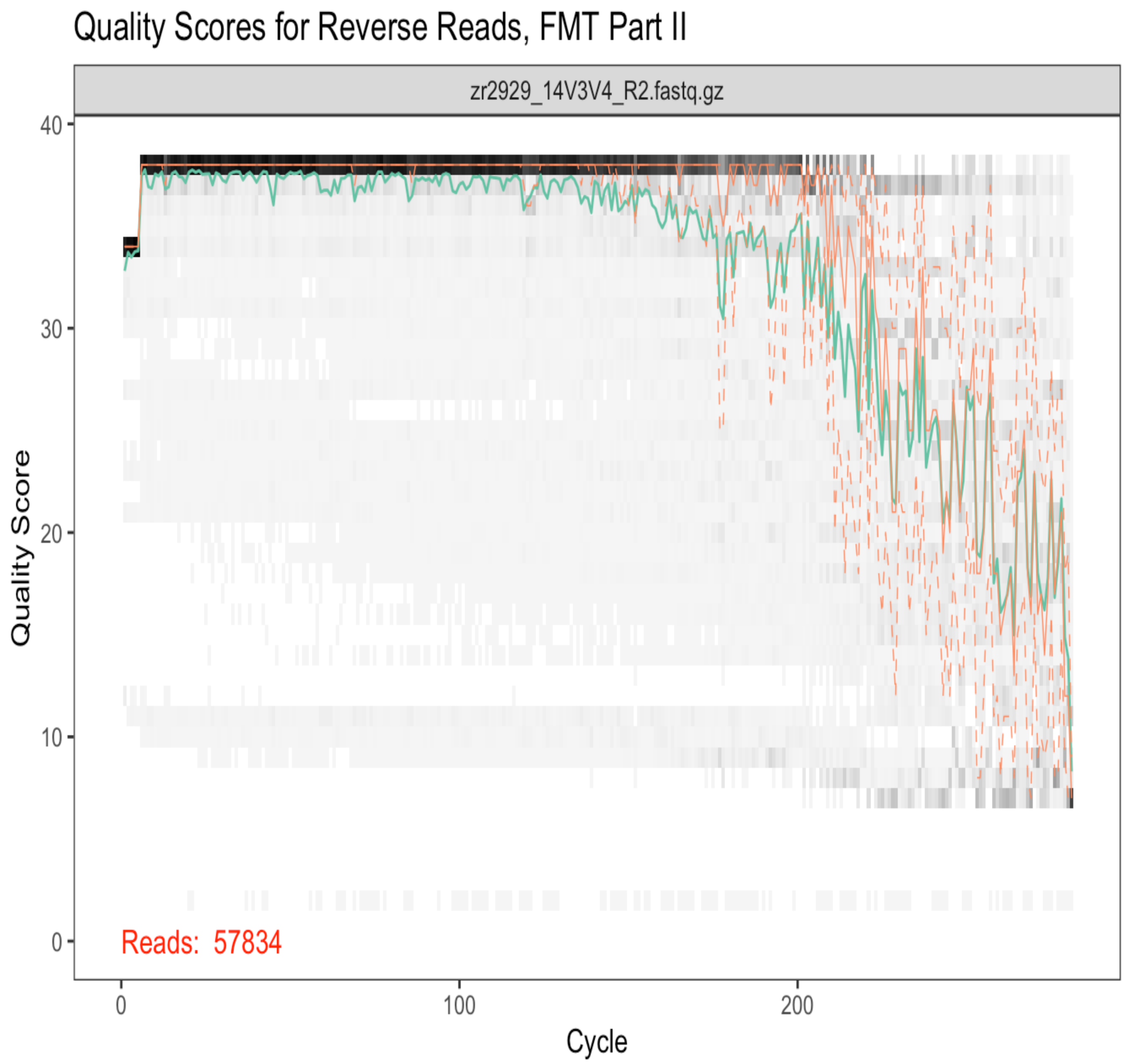


```
plotQualityProfile(fqs.for[[3]]) + ggtitle("Quality Scores for Forward Reads, FMT Part
II")

## Warning: `guides(<scale> = FALSE)` is deprecated. Please use `guides(<scale> =
## "none")` instead.
```

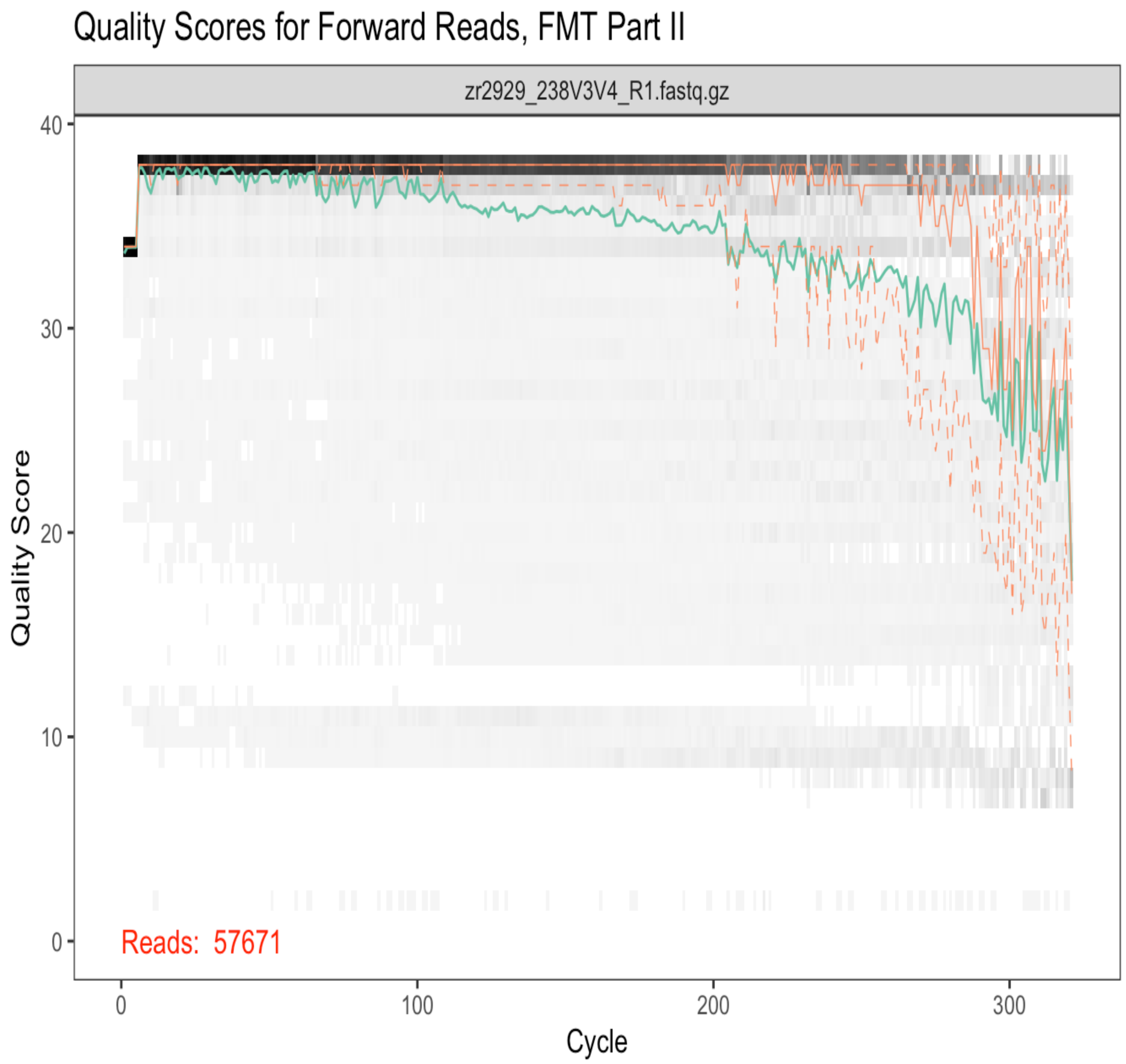


```
plotQualityProfile(fqs.rev[[3]]) + ggtitle("Quality Scores for Reverse Reads, FMT Part
II")

## Warning: `guides(<scale> = FALSE)` is deprecated. Please use `guides(<scale> =
## "none")` instead.
```

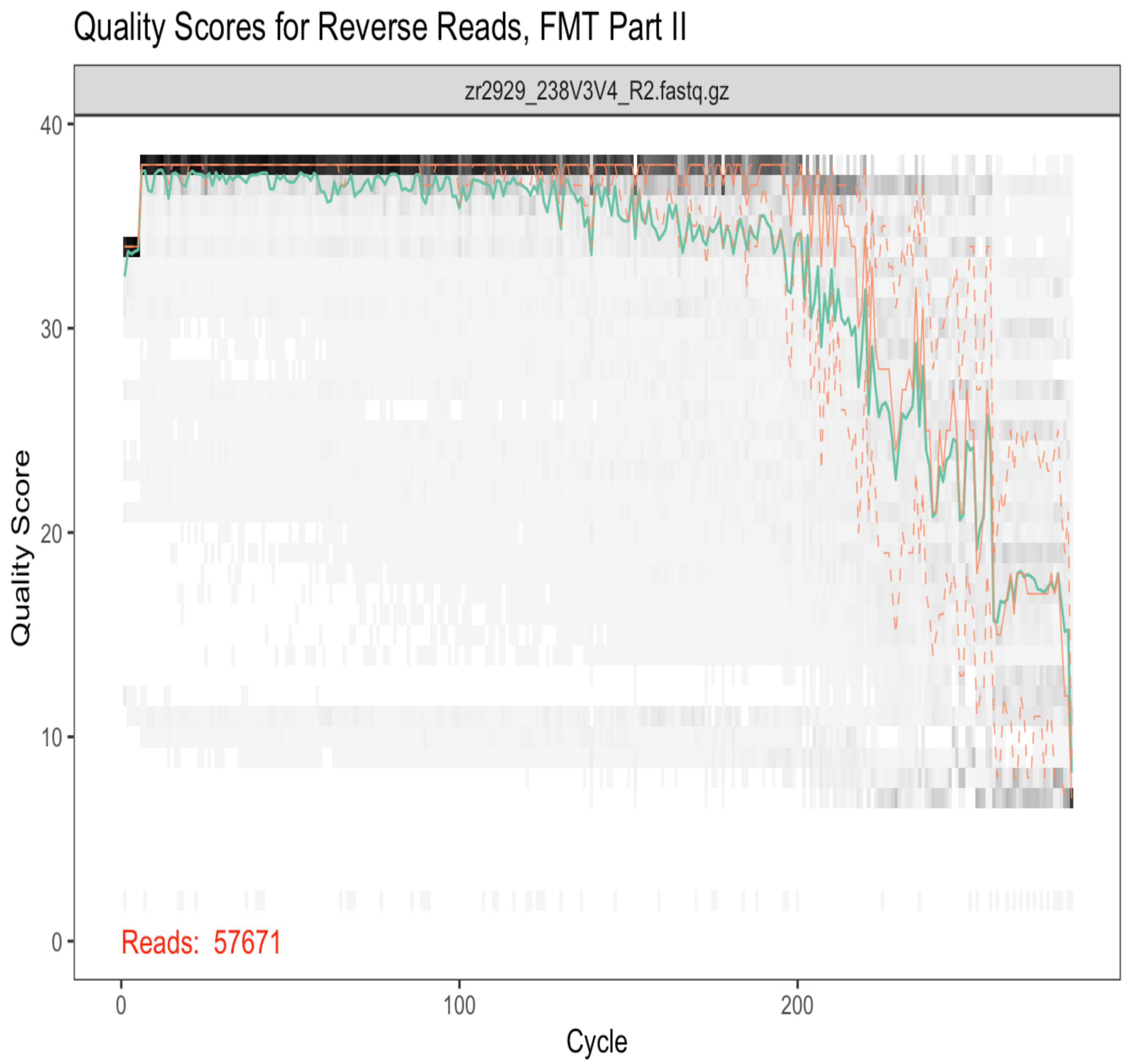


```
# Make directory and filenames for the filtered fastqs
filt_path <- file.path(".", "filtered")
if(!file_test("-d", filt_path)) dir.create(filt_path)
filtFs <- file.path(filt_path, paste0(sample.names, "_F_filt.fastq.gz"))
filtRs <- file.path(filt_path, paste0(sample.names, "_R_filt.fastq.gz"))


# Filter and trim
## trimLeft = c(10,10) says drop the first 10 base pairs from both the F and R reverse
reads
```

```
## truncLen = c(300,225) says to trim the F at 300bp in length and the R at 225bp in l
ength
## maxN = 0, no "N" nucleotides allowed in the sequence
## max EE = maximum expected errors over the entire read, set to 2
## truncQ = 2, drop an reads where a nucleotide a Q score of less than 2 (ie. 20)


out <- filterAndTrim(fqs.for, filtFs, fqs.rev,filtRs,
                  trimLeft=c(16, 24), truncLen=c(320,180),
                  maxN=0, maxEE=2, truncQ=2, matchIDs = T,
                  compress=TRUE, verbose=TRUE, multithread = 10)
   #dereplicate the data
derepFs <- derepFastq(filtFs, verbose=TRUE)
## Dereplicating sequence entries in Fastq file: ./filtered/13V3V4_F_filt.fastq.gz
## Encountered 8831 unique sequences from 39830 total sequences read.
## Dereplicating sequence entries in Fastq file: ./filtered/14V3V4_F_filt.fastq.gz
## Encountered 7461 unique sequences from 48601 total sequences read.
## Dereplicating sequence entries in Fastq file: ./filtered/238V3V4_F_filt.fastq.gz
## Encountered 13313 unique sequences from 38897 total sequences read.
## Dereplicating sequence entries in Fastq file: ./filtered/239V3V4_F_filt.fastq.gz
## Encountered 6384 unique sequences from 19134 total sequences read.
## Dereplicating sequence entries in Fastq file: ./filtered/240V3V4_F_filt.fastq.gz
## Encountered 12208 unique sequences from 41533 total sequences read.
## Dereplicating sequence entries in Fastq file: ./filtered/241V3V4_F_filt.fastq.gz
## Encountered 9745 unique sequences from 26986 total sequences read.
## Dereplicating sequence entries in Fastq file: ./filtered/242V3V4_F_filt.fastq.gz
## Encountered 12243 unique sequences from 42421 total sequences read.
## Dereplicating sequence entries in Fastq file: ./filtered/243V3V4_F_filt.fastq.gz
## Encountered 14626 unique sequences from 42944 total sequences read.
## Dereplicating sequence entries in Fastq file: ./filtered/244V3V4_F_filt.fastq.gz
## Encountered 12884 unique sequences from 38448 total sequences read.
## Dereplicating sequence entries in Fastq file: ./filtered/245V3V4_F_filt.fastq.gz
## Encountered 12644 unique sequences from 37369 total sequences read.
## Dereplicating sequence entries in Fastq file: ./filtered/246V3V4_F_filt.fastq.gz
## Encountered 8858 unique sequences from 25227 total sequences read.
## Dereplicating sequence entries in Fastq file: ./filtered/247V3V4_F_filt.fastq.gz
## Encountered 10794 unique sequences from 29406 total sequences read.
## Dereplicating sequence entries in Fastq file: ./filtered/248V3V4_F_filt.fastq.gz
```

```
## Encountered 13521 unique sequences from 52825 total sequences read.
## Dereplicating sequence entries in Fastq file: ./filtered/249V3V4_F_filt.fastq.gz
## Encountered 9747 unique sequences from 27785 total sequences read.
## Dereplicating sequence entries in Fastq file: ./filtered/250V3V4_F_filt.fastq.gz
## Encountered 4642 unique sequences from 19015 total sequences read.
## Dereplicating sequence entries in Fastq file: ./filtered/251V3V4_F_filt.fastq.gz
## Encountered 11087 unique sequences from 33834 total sequences read.
## Dereplicating sequence entries in Fastq file: ./filtered/252V3V4_F_filt.fastq.gz
## Encountered 8895 unique sequences from 30665 total sequences read.
## Dereplicating sequence entries in Fastq file: ./filtered/253V3V4_F_filt.fastq.gz
## Encountered 10347 unique sequences from 24005 total sequences read.
## Dereplicating sequence entries in Fastq file: ./filtered/254V3V4_F_filt.fastq.gz
## Encountered 10127 unique sequences from 36360 total sequences read.
## Dereplicating sequence entries in Fastq file: ./filtered/255V3V4_F_filt.fastq.gz
## Encountered 8135 unique sequences from 22285 total sequences read.
## Dereplicating sequence entries in Fastq file: ./filtered/256V3V4_F_filt.fastq.gz
## Encountered 12663 unique sequences from 44924 total sequences read.
## Dereplicating sequence entries in Fastq file: ./filtered/257V3V4_F_filt.fastq.gz
## Encountered 10456 unique sequences from 24498 total sequences read.
## Dereplicating sequence entries in Fastq file: ./filtered/258V3V4_F_filt.fastq.gz
## Encountered 7554 unique sequences from 30627 total sequences read.
## Dereplicating sequence entries in Fastq file: ./filtered/259V3V4_F_filt.fastq.gz
## Encountered 8080 unique sequences from 32850 total sequences read.
## Dereplicating sequence entries in Fastq file: ./filtered/260V3V4_F_filt.fastq.gz
## Encountered 8686 unique sequences from 25493 total sequences read.
## Dereplicating sequence entries in Fastq file: ./filtered/261V3V4_F_filt.fastq.gz
## Encountered 11966 unique sequences from 31778 total sequences read.
## Dereplicating sequence entries in Fastq file: ./filtered/262V3V4_F_filt.fastq.gz
## Encountered 8960 unique sequences from 28407 total sequences read.
## Dereplicating sequence entries in Fastq file: ./filtered/263V3V4_F_filt.fastq.gz
## Encountered 12422 unique sequences from 34592 total sequences read.
## Dereplicating sequence entries in Fastq file: ./filtered/264V3V4_F_filt.fastq.gz
## Encountered 13216 unique sequences from 39991 total sequences read.
## Dereplicating sequence entries in Fastq file: ./filtered/265V3V4_F_filt.fastq.gz
## Encountered 10126 unique sequences from 30056 total sequences read.
## Dereplicating sequence entries in Fastq file: ./filtered/266V3V4_F_filt.fastq.gz
## Encountered 14765 unique sequences from 52654 total sequences read.
```

```
## Dereplicating sequence entries in Fastq file: ./filtered/267V3V4_F_filt.fastq.gz
## Encountered 14942 unique sequences from 46711 total sequences read.
## Dereplicating sequence entries in Fastq file: ./filtered/268V3V4_F_filt.fastq.gz
## Encountered 12476 unique sequences from 42456 total sequences read.
## Dereplicating sequence entries in Fastq file: ./filtered/269V3V4_F_filt.fastq.gz
## Encountered 9194 unique sequences from 26251 total sequences read.
## Dereplicating sequence entries in Fastq file: ./filtered/26V3V4_F_filt.fastq.gz
## Encountered 7992 unique sequences from 36946 total sequences read.
## Dereplicating sequence entries in Fastq file: ./filtered/270V3V4_F_filt.fastq.gz
## Encountered 7449 unique sequences from 25347 total sequences read.
## Dereplicating sequence entries in Fastq file: ./filtered/271V3V4_F_filt.fastq.gz
## Encountered 8429 unique sequences from 24259 total sequences read.
## Dereplicating sequence entries in Fastq file: ./filtered/272V3V4_F_filt.fastq.gz
## Encountered 11384 unique sequences from 33445 total sequences read.
## Dereplicating sequence entries in Fastq file: ./filtered/273V3V4_F_filt.fastq.gz
## Encountered 10639 unique sequences from 30881 total sequences read.
## Dereplicating sequence entries in Fastq file: ./filtered/274V3V4_F_filt.fastq.gz
## Encountered 11708 unique sequences from 38081 total sequences read.
## Dereplicating sequence entries in Fastq file: ./filtered/275V3V4_F_filt.fastq.gz
## Encountered 11501 unique sequences from 30929 total sequences read.
## Dereplicating sequence entries in Fastq file: ./filtered/276V3V4_F_filt.fastq.gz
## Encountered 8356 unique sequences from 33507 total sequences read.
## Dereplicating sequence entries in Fastq file: ./filtered/277V3V4_F_filt.fastq.gz
## Encountered 10018 unique sequences from 26338 total sequences read.
## Dereplicating sequence entries in Fastq file: ./filtered/278V3V4_F_filt.fastq.gz
## Encountered 9458 unique sequences from 32634 total sequences read.
## Dereplicating sequence entries in Fastq file: ./filtered/279V3V4_F_filt.fastq.gz
## Encountered 6313 unique sequences from 22969 total sequences read.
## Dereplicating sequence entries in Fastq file: ./filtered/280V3V4_F_filt.fastq.gz
## Encountered 11059 unique sequences from 40286 total sequences read.
## Dereplicating sequence entries in Fastq file: ./filtered/281V3V4_F_filt.fastq.gz
## Encountered 8160 unique sequences from 18095 total sequences read.
## Dereplicating sequence entries in Fastq file: ./filtered/282V3V4_F_filt.fastq.gz
## Encountered 11673 unique sequences from 36096 total sequences read.
## Dereplicating sequence entries in Fastq file: ./filtered/283V3V4_F_filt.fastq.gz
## Encountered 13023 unique sequences from 42277 total sequences read.
## Dereplicating sequence entries in Fastq file: ./filtered/284V3V4_F_filt.fastq.gz
```

```
## Encountered 12432 unique sequences from 35734 total sequences read.
## Dereplicating sequence entries in Fastq file: ./filtered/285V3V4_F_filt.fastq.gz
## Encountered 9751 unique sequences from 24235 total sequences read.
## Dereplicating sequence entries in Fastq file: ./filtered/286V3V4_F_filt.fastq.gz
## Encountered 9784 unique sequences from 27178 total sequences read.
## Dereplicating sequence entries in Fastq file: ./filtered/287V3V4_F_filt.fastq.gz
## Encountered 8908 unique sequences from 28172 total sequences read.
## Dereplicating sequence entries in Fastq file: ./filtered/288V3V4_F_filt.fastq.gz
## Encountered 12020 unique sequences from 46704 total sequences read.
## Dereplicating sequence entries in Fastq file: ./filtered/289V3V4_F_filt.fastq.gz
## Encountered 9843 unique sequences from 26921 total sequences read.
## Dereplicating sequence entries in Fastq file: ./filtered/290V3V4_F_filt.fastq.gz
## Encountered 12269 unique sequences from 49196 total sequences read.
## Dereplicating sequence entries in Fastq file: ./filtered/291V3V4_F_filt.fastq.gz
## Encountered 10934 unique sequences from 39459 total sequences read.
## Dereplicating sequence entries in Fastq file: ./filtered/292V3V4_F_filt.fastq.gz
## Encountered 10314 unique sequences from 39888 total sequences read.
## Dereplicating sequence entries in Fastq file: ./filtered/293V3V4_F_filt.fastq.gz
## Encountered 7274 unique sequences from 20013 total sequences read.
## Dereplicating sequence entries in Fastq file: ./filtered/294V3V4_F_filt.fastq.gz
## Encountered 6804 unique sequences from 22293 total sequences read.
## Dereplicating sequence entries in Fastq file: ./filtered/295V3V4_F_filt.fastq.gz
## Encountered 7650 unique sequences from 22913 total sequences read.
## Dereplicating sequence entries in Fastq file: ./filtered/296V3V4_F_filt.fastq.gz
## Encountered 9279 unique sequences from 35872 total sequences read.
## Dereplicating sequence entries in Fastq file: ./filtered/297V3V4_F_filt.fastq.gz
## Encountered 8429 unique sequences from 21174 total sequences read.
## Dereplicating sequence entries in Fastq file: ./filtered/298V3V4_F_filt.fastq.gz
## Encountered 9177 unique sequences from 31913 total sequences read.
## Dereplicating sequence entries in Fastq file: ./filtered/299V3V4_F_filt.fastq.gz
## Encountered 7153 unique sequences from 14555 total sequences read.
## Dereplicating sequence entries in Fastq file: ./filtered/300V3V4_F_filt.fastq.gz
## Encountered 10696 unique sequences from 36219 total sequences read.
## Dereplicating sequence entries in Fastq file: ./filtered/301V3V4_F_filt.fastq.gz
## Encountered 12257 unique sequences from 42984 total sequences read.
## Dereplicating sequence entries in Fastq file: ./filtered/302V3V4_F_filt.fastq.gz
## Encountered 11227 unique sequences from 31240 total sequences read.
```

```
## Dereplicating sequence entries in Fastq file: ./filtered/303V3V4_F_filt.fastq.gz
## Encountered 13660 unique sequences from 38607 total sequences read.
## Dereplicating sequence entries in Fastq file: ./filtered/304V3V4_F_filt.fastq.gz
## Encountered 12536 unique sequences from 37551 total sequences read.
## Dereplicating sequence entries in Fastq file: ./filtered/305V3V4_F_filt.fastq.gz
## Encountered 13584 unique sequences from 37523 total sequences read.
## Dereplicating sequence entries in Fastq file: ./filtered/306V3V4_F_filt.fastq.gz
## Encountered 15731 unique sequences from 48739 total sequences read.
## Dereplicating sequence entries in Fastq file: ./filtered/307V3V4_F_filt.fastq.gz
## Encountered 8655 unique sequences from 20752 total sequences read.
## Dereplicating sequence entries in Fastq file: ./filtered/308V3V4_F_filt.fastq.gz
## Encountered 9892 unique sequences from 28937 total sequences read.
## Dereplicating sequence entries in Fastq file: ./filtered/309V3V4_F_filt.fastq.gz
## Encountered 11529 unique sequences from 32948 total sequences read.
## Dereplicating sequence entries in Fastq file: ./filtered/30V3V4_F_filt.fastq.gz
## Encountered 8447 unique sequences from 39109 total sequences read.
## Dereplicating sequence entries in Fastq file: ./filtered/310V3V4_F_filt.fastq.gz
## Encountered 10123 unique sequences from 30140 total sequences read.
## Dereplicating sequence entries in Fastq file: ./filtered/311V3V4_F_filt.fastq.gz
## Encountered 11171 unique sequences from 34343 total sequences read.
## Dereplicating sequence entries in Fastq file: ./filtered/312V3V4_F_filt.fastq.gz
## Encountered 12269 unique sequences from 45349 total sequences read.
## Dereplicating sequence entries in Fastq file: ./filtered/313V3V4_F_filt.fastq.gz
## Encountered 11811 unique sequences from 35930 total sequences read.
## Dereplicating sequence entries in Fastq file: ./filtered/314V3V4_F_filt.fastq.gz
## Encountered 15747 unique sequences from 48076 total sequences read.
## Dereplicating sequence entries in Fastq file: ./filtered/315V3V4_F_filt.fastq.gz
## Encountered 7287 unique sequences from 19510 total sequences read.
## Dereplicating sequence entries in Fastq file: ./filtered/316V3V4_F_filt.fastq.gz
## Encountered 10168 unique sequences from 34555 total sequences read.
## Dereplicating sequence entries in Fastq file: ./filtered/317V3V4_F_filt.fastq.gz
## Encountered 10460 unique sequences from 28896 total sequences read.
## Dereplicating sequence entries in Fastq file: ./filtered/318V3V4_F_filt.fastq.gz
## Encountered 5867 unique sequences from 22669 total sequences read.
## Dereplicating sequence entries in Fastq file: ./filtered/319V3V4_F_filt.fastq.gz
## Encountered 9749 unique sequences from 26236 total sequences read.
## Dereplicating sequence entries in Fastq file: ./filtered/320V3V4_F_filt.fastq.gz
```

```
## Encountered 13309 unique sequences from 40834 total sequences read.
## Dereplicating sequence entries in Fastq file: ./filtered/321V3V4_F_filt.fastq.gz
## Encountered 11147 unique sequences from 31389 total sequences read.
## Dereplicating sequence entries in Fastq file: ./filtered/322V3V4_F_filt.fastq.gz
## Encountered 12388 unique sequences from 41066 total sequences read.
## Dereplicating sequence entries in Fastq file: ./filtered/323V3V4_F_filt.fastq.gz
## Encountered 10116 unique sequences from 25288 total sequences read.
## Dereplicating sequence entries in Fastq file: ./filtered/324V3V4_F_filt.fastq.gz
## Encountered 9357 unique sequences from 29183 total sequences read.
## Dereplicating sequence entries in Fastq file: ./filtered/325V3V4_F_filt.fastq.gz
## Encountered 13055 unique sequences from 38179 total sequences read.
## Dereplicating sequence entries in Fastq file: ./filtered/326V3V4_F_filt.fastq.gz
## Encountered 9853 unique sequences from 29020 total sequences read.
## Dereplicating sequence entries in Fastq file: ./filtered/327V3V4_F_filt.fastq.gz
## Encountered 11641 unique sequences from 34516 total sequences read.
## Dereplicating sequence entries in Fastq file: ./filtered/328V3V4_F_filt.fastq.gz
## Encountered 7010 unique sequences from 31660 total sequences read.
## Dereplicating sequence entries in Fastq file: ./filtered/329V3V4_F_filt.fastq.gz
## Encountered 12138 unique sequences from 32792 total sequences read.
## Dereplicating sequence entries in Fastq file: ./filtered/330V3V4_F_filt.fastq.gz
## Encountered 11359 unique sequences from 37583 total sequences read.
## Dereplicating sequence entries in Fastq file: ./filtered/331V3V4_F_filt.fastq.gz
## Encountered 9392 unique sequences from 23616 total sequences read.
## Dereplicating sequence entries in Fastq file: ./filtered/332V3V4_F_filt.fastq.gz
## Encountered 10230 unique sequences from 37669 total sequences read.
## Dereplicating sequence entries in Fastq file: ./filtered/333V3V4_F_filt.fastq.gz
## Encountered 9820 unique sequences from 37731 total sequences read.
## Dereplicating sequence entries in Fastq file: ./filtered/334V3V4_F_filt.fastq.gz
## Encountered 9545 unique sequences from 28456 total sequences read.
## Dereplicating sequence entries in Fastq file: ./filtered/335V3V4_F_filt.fastq.gz
## Encountered 11709 unique sequences from 35913 total sequences read.
## Dereplicating sequence entries in Fastq file: ./filtered/336V3V4_F_filt.fastq.gz
## Encountered 10306 unique sequences from 36861 total sequences read.
## Dereplicating sequence entries in Fastq file: ./filtered/337V3V4_F_filt.fastq.gz
## Encountered 11051 unique sequences from 35569 total sequences read.
## Dereplicating sequence entries in Fastq file: ./filtered/338V3V4_F_filt.fastq.gz
## Encountered 12463 unique sequences from 41625 total sequences read.
```

```
## Dereplicating sequence entries in Fastq file: ./filtered/339V3V4_F_filt.fastq.gz
## Encountered 10594 unique sequences from 27782 total sequences read.
## Dereplicating sequence entries in Fastq file: ./filtered/340V3V4_F_filt.fastq.gz
## Encountered 11534 unique sequences from 39916 total sequences read.
## Dereplicating sequence entries in Fastq file: ./filtered/341V3V4_F_filt.fastq.gz
## Encountered 12745 unique sequences from 40216 total sequences read.
## Dereplicating sequence entries in Fastq file: ./filtered/342V3V4_F_filt.fastq.gz
## Encountered 5772 unique sequences from 15582 total sequences read.
## Dereplicating sequence entries in Fastq file: ./filtered/343V3V4_F_filt.fastq.gz
## Encountered 10897 unique sequences from 32544 total sequences read.
## Dereplicating sequence entries in Fastq file: ./filtered/344V3V4_F_filt.fastq.gz
## Encountered 13680 unique sequences from 45852 total sequences read.
## Dereplicating sequence entries in Fastq file: ./filtered/345V3V4_F_filt.fastq.gz
## Encountered 10378 unique sequences from 34702 total sequences read.
## Dereplicating sequence entries in Fastq file: ./filtered/346V3V4_F_filt.fastq.gz
## Encountered 11603 unique sequences from 36982 total sequences read.
## Dereplicating sequence entries in Fastq file: ./filtered/347V3V4_F_filt.fastq.gz
## Encountered 8231 unique sequences from 19274 total sequences read.
## Dereplicating sequence entries in Fastq file: ./filtered/348V3V4_F_filt.fastq.gz
## Encountered 10175 unique sequences from 34428 total sequences read.
## Dereplicating sequence entries in Fastq file: ./filtered/349V3V4_F_filt.fastq.gz
## Encountered 9736 unique sequences from 30679 total sequences read.
## Dereplicating sequence entries in Fastq file: ./filtered/350V3V4_F_filt.fastq.gz
## Encountered 8560 unique sequences from 25346 total sequences read.
## Dereplicating sequence entries in Fastq file: ./filtered/351V3V4_F_filt.fastq.gz
## Encountered 9952 unique sequences from 24485 total sequences read.
## Dereplicating sequence entries in Fastq file: ./filtered/352V3V4_F_filt.fastq.gz
## Encountered 9926 unique sequences from 30542 total sequences read.
## Dereplicating sequence entries in Fastq file: ./filtered/353V3V4_F_filt.fastq.gz
## Encountered 12363 unique sequences from 32027 total sequences read.
## Dereplicating sequence entries in Fastq file: ./filtered/354V3V4_F_filt.fastq.gz
## Encountered 10463 unique sequences from 32743 total sequences read.
## Dereplicating sequence entries in Fastq file: ./filtered/355V3V4_F_filt.fastq.gz
## Encountered 7696 unique sequences from 18743 total sequences read.
## Dereplicating sequence entries in Fastq file: ./filtered/356V3V4_F_filt.fastq.gz
## Encountered 10246 unique sequences from 33571 total sequences read.
## Dereplicating sequence entries in Fastq file: ./filtered/357V3V4_F_filt.fastq.gz
```

```
## Encountered 10970 unique sequences from 34251 total sequences read.
## Dereplicating sequence entries in Fastq file: ./filtered/358V3V4_F_filt.fastq.gz
## Encountered 8892 unique sequences from 28764 total sequences read.
## Dereplicating sequence entries in Fastq file: ./filtered/359V3V4_F_filt.fastq.gz
## Encountered 7858 unique sequences from 31957 total sequences read.
## Dereplicating sequence entries in Fastq file: ./filtered/360V3V4_F_filt.fastq.gz
## Encountered 10055 unique sequences from 41312 total sequences read.
## Dereplicating sequence entries in Fastq file: ./filtered/361V3V4_F_filt.fastq.gz
## Encountered 11459 unique sequences from 38598 total sequences read.
## Dereplicating sequence entries in Fastq file: ./filtered/362V3V4_F_filt.fastq.gz
## Encountered 10455 unique sequences from 35770 total sequences read.
## Dereplicating sequence entries in Fastq file: ./filtered/363V3V4_F_filt.fastq.gz
## Encountered 8698 unique sequences from 23062 total sequences read.
## Dereplicating sequence entries in Fastq file: ./filtered/364V3V4_F_filt.fastq.gz
## Encountered 4963 unique sequences from 14104 total sequences read.
## Dereplicating sequence entries in Fastq file: ./filtered/365V3V4_F_filt.fastq.gz
## Encountered 10794 unique sequences from 29000 total sequences read.
## Dereplicating sequence entries in Fastq file: ./filtered/366V3V4_F_filt.fastq.gz
## Encountered 8705 unique sequences from 27430 total sequences read.
## Dereplicating sequence entries in Fastq file: ./filtered/367V3V4_F_filt.fastq.gz
## Encountered 8126 unique sequences from 29157 total sequences read.
## Dereplicating sequence entries in Fastq file: ./filtered/368V3V4_F_filt.fastq.gz
## Encountered 10195 unique sequences from 29834 total sequences read.
## Dereplicating sequence entries in Fastq file: ./filtered/369V3V4_F_filt.fastq.gz
## Encountered 9955 unique sequences from 32668 total sequences read.
## Dereplicating sequence entries in Fastq file: ./filtered/370V3V4_F_filt.fastq.gz
## Encountered 9676 unique sequences from 33038 total sequences read.
## Dereplicating sequence entries in Fastq file: ./filtered/371V3V4_F_filt.fastq.gz
## Encountered 8909 unique sequences from 22906 total sequences read.
## Dereplicating sequence entries in Fastq file: ./filtered/372V3V4_F_filt.fastq.gz
## Encountered 10196 unique sequences from 33636 total sequences read.
## Dereplicating sequence entries in Fastq file: ./filtered/373V3V4_F_filt.fastq.gz
## Encountered 9748 unique sequences from 30634 total sequences read.
## Dereplicating sequence entries in Fastq file: ./filtered/374V3V4_F_filt.fastq.gz
## Encountered 6814 unique sequences from 19774 total sequences read.
## Dereplicating sequence entries in Fastq file: ./filtered/375V3V4_F_filt.fastq.gz
## Encountered 8372 unique sequences from 23800 total sequences read.
```

```
## Dereplicating sequence entries in Fastq file: ./filtered/376V3V4_F_filt.fastq.gz
## Encountered 10742 unique sequences from 36884 total sequences read.
## Dereplicating sequence entries in Fastq file: ./filtered/377V3V4_F_filt.fastq.gz
## Encountered 12839 unique sequences from 37157 total sequences read.
## Dereplicating sequence entries in Fastq file: ./filtered/378V3V4_F_filt.fastq.gz
## Encountered 11026 unique sequences from 38178 total sequences read.
## Dereplicating sequence entries in Fastq file: ./filtered/379V3V4_F_filt.fastq.gz
## Encountered 11259 unique sequences from 25106 total sequences read.
## Dereplicating sequence entries in Fastq file: ./filtered/380V3V4_F_filt.fastq.gz
## Encountered 10301 unique sequences from 34116 total sequences read.
## Dereplicating sequence entries in Fastq file: ./filtered/381V3V4_F_filt.fastq.gz
## Encountered 9853 unique sequences from 30707 total sequences read.
## Dereplicating sequence entries in Fastq file: ./filtered/382V3V4_F_filt.fastq.gz
## Encountered 8405 unique sequences from 27524 total sequences read.
## Dereplicating sequence entries in Fastq file: ./filtered/383V3V4_F_filt.fastq.gz
## Encountered 8789 unique sequences from 32527 total sequences read.
## Dereplicating sequence entries in Fastq file: ./filtered/384V3V4_F_filt.fastq.gz
## Encountered 10903 unique sequences from 40299 total sequences read.
## Dereplicating sequence entries in Fastq file: ./filtered/385V3V4_F_filt.fastq.gz
## Encountered 11807 unique sequences from 40246 total sequences read.
## Dereplicating sequence entries in Fastq file: ./filtered/386V3V4_F_filt.fastq.gz
## Encountered 10683 unique sequences from 35589 total sequences read.
## Dereplicating sequence entries in Fastq file: ./filtered/387V3V4_F_filt.fastq.gz
## Encountered 6018 unique sequences from 16541 total sequences read.
## Dereplicating sequence entries in Fastq file: ./filtered/388V3V4_F_filt.fastq.gz
## Encountered 6323 unique sequences from 17488 total sequences read.
## Dereplicating sequence entries in Fastq file: ./filtered/389V3V4_F_filt.fastq.gz
## Encountered 9408 unique sequences from 22427 total sequences read.
## Dereplicating sequence entries in Fastq file: ./filtered/38V3V4_F_filt.fastq.gz
## Encountered 11006 unique sequences from 51960 total sequences read.
## Dereplicating sequence entries in Fastq file: ./filtered/390V3V4_F_filt.fastq.gz
## Encountered 5540 unique sequences from 15260 total sequences read.
## Dereplicating sequence entries in Fastq file: ./filtered/391V3V4_F_filt.fastq.gz
## Encountered 7878 unique sequences from 21125 total sequences read.
## Dereplicating sequence entries in Fastq file: ./filtered/392V3V4_F_filt.fastq.gz
## Encountered 5705 unique sequences from 24778 total sequences read.
## Dereplicating sequence entries in Fastq file: ./filtered/393V3V4_F_filt.fastq.gz
```

```
## Encountered 8625 unique sequences from 23560 total sequences read.
## Dereplicating sequence entries in Fastq file: ./filtered/394V3V4_F_filt.fastq.gz
## Encountered 10716 unique sequences from 33363 total sequences read.
## Dereplicating sequence entries in Fastq file: ./filtered/395V3V4_F_filt.fastq.gz
## Encountered 8751 unique sequences from 23249 total sequences read.
## Dereplicating sequence entries in Fastq file: ./filtered/396V3V4_F_filt.fastq.gz
## Encountered 13144 unique sequences from 37426 total sequences read.
## Dereplicating sequence entries in Fastq file: ./filtered/397V3V4_F_filt.fastq.gz
## Encountered 10526 unique sequences from 25788 total sequences read.
## Dereplicating sequence entries in Fastq file: ./filtered/398V3V4_F_filt.fastq.gz
## Encountered 10765 unique sequences from 37756 total sequences read.
## Dereplicating sequence entries in Fastq file: ./filtered/399V3V4_F_filt.fastq.gz
## Encountered 11383 unique sequences from 36360 total sequences read.
## Dereplicating sequence entries in Fastq file: ./filtered/39V3V4_F_filt.fastq.gz
## Encountered 9423 unique sequences from 47704 total sequences read.
## Dereplicating sequence entries in Fastq file: ./filtered/400V3V4_F_filt.fastq.gz
## Encountered 8979 unique sequences from 34959 total sequences read.
## Dereplicating sequence entries in Fastq file: ./filtered/401V3V4_F_filt.fastq.gz
## Encountered 7502 unique sequences from 20870 total sequences read.
## Dereplicating sequence entries in Fastq file: ./filtered/402V3V4_F_filt.fastq.gz
## Encountered 8014 unique sequences from 25325 total sequences read.
## Dereplicating sequence entries in Fastq file: ./filtered/403V3V4_F_filt.fastq.gz
## Encountered 7812 unique sequences from 22182 total sequences read.
## Dereplicating sequence entries in Fastq file: ./filtered/404V3V4_F_filt.fastq.gz
## Encountered 11002 unique sequences from 36937 total sequences read.
## Dereplicating sequence entries in Fastq file: ./filtered/405V3V4_F_filt.fastq.gz
## Encountered 7557 unique sequences from 23136 total sequences read.
## Dereplicating sequence entries in Fastq file: ./filtered/406V3V4_F_filt.fastq.gz
## Encountered 10384 unique sequences from 41498 total sequences read.
## Dereplicating sequence entries in Fastq file: ./filtered/407V3V4_F_filt.fastq.gz
## Encountered 9535 unique sequences from 32112 total sequences read.
## Dereplicating sequence entries in Fastq file: ./filtered/408V3V4_F_filt.fastq.gz
## Encountered 10628 unique sequences from 34875 total sequences read.
## Dereplicating sequence entries in Fastq file: ./filtered/409V3V4_F_filt.fastq.gz
## Encountered 8907 unique sequences from 24764 total sequences read.
## Dereplicating sequence entries in Fastq file: ./filtered/410V3V4_F_filt.fastq.gz
## Encountered 10048 unique sequences from 31786 total sequences read.
```

```
## Dereplicating sequence entries in Fastq file: ./filtered/411V3V4_F_filt.fastq.gz
## Encountered 12190 unique sequences from 31304 total sequences read.
## Dereplicating sequence entries in Fastq file: ./filtered/412V3V4_F_filt.fastq.gz
## Encountered 12699 unique sequences from 37457 total sequences read.
## Dereplicating sequence entries in Fastq file: ./filtered/413V3V4_F_filt.fastq.gz
## Encountered 10660 unique sequences from 26551 total sequences read.
## Dereplicating sequence entries in Fastq file: ./filtered/414V3V4_F_filt.fastq.gz
## Encountered 12543 unique sequences from 48887 total sequences read.
## Dereplicating sequence entries in Fastq file: ./filtered/415V3V4_F_filt.fastq.gz
## Encountered 10735 unique sequences from 34973 total sequences read.
## Dereplicating sequence entries in Fastq file: ./filtered/416V3V4_F_filt.fastq.gz
## Encountered 9003 unique sequences from 31882 total sequences read.
## Dereplicating sequence entries in Fastq file: ./filtered/417V3V4_F_filt.fastq.gz
## Encountered 10912 unique sequences from 24933 total sequences read.
## Dereplicating sequence entries in Fastq file: ./filtered/418V3V4_F_filt.fastq.gz
## Encountered 8315 unique sequences from 27617 total sequences read.
## Dereplicating sequence entries in Fastq file: ./filtered/419V3V4_F_filt.fastq.gz
## Encountered 11962 unique sequences from 31810 total sequences read.
## Dereplicating sequence entries in Fastq file: ./filtered/420V3V4_F_filt.fastq.gz
## Encountered 9614 unique sequences from 30935 total sequences read.
## Dereplicating sequence entries in Fastq file: ./filtered/421V3V4_F_filt.fastq.gz
## Encountered 7173 unique sequences from 16588 total sequences read.
## Dereplicating sequence entries in Fastq file: ./filtered/422V3V4_F_filt.fastq.gz
## Encountered 9285 unique sequences from 34570 total sequences read.
## Dereplicating sequence entries in Fastq file: ./filtered/423V3V4_F_filt.fastq.gz
## Encountered 12730 unique sequences from 35072 total sequences read.
## Dereplicating sequence entries in Fastq file: ./filtered/424V3V4_F_filt.fastq.gz
## Encountered 11347 unique sequences from 39136 total sequences read.
## Dereplicating sequence entries in Fastq file: ./filtered/425V3V4_F_filt.fastq.gz
## Encountered 8255 unique sequences from 21924 total sequences read.
## Dereplicating sequence entries in Fastq file: ./filtered/426V3V4_F_filt.fastq.gz
## Encountered 8925 unique sequences from 28276 total sequences read.
## Dereplicating sequence entries in Fastq file: ./filtered/427V3V4_F_filt.fastq.gz
## Encountered 8928 unique sequences from 22542 total sequences read.
## Dereplicating sequence entries in Fastq file: ./filtered/428V3V4_F_filt.fastq.gz
## Encountered 9784 unique sequences from 34406 total sequences read.
## Dereplicating sequence entries in Fastq file: ./filtered/429V3V4_F_filt.fastq.gz
```

```
## Encountered 10348 unique sequences from 26941 total sequences read.
## Dereplicating sequence entries in Fastq file: ./filtered/430V3V4_F_filt.fastq.gz
## Encountered 10286 unique sequences from 37958 total sequences read.
## Dereplicating sequence entries in Fastq file: ./filtered/431V3V4_F_filt.fastq.gz
## Encountered 12210 unique sequences from 38060 total sequences read.
## Dereplicating sequence entries in Fastq file: ./filtered/432V3V4_F_filt.fastq.gz
## Encountered 11624 unique sequences from 38185 total sequences read.
## Dereplicating sequence entries in Fastq file: ./filtered/433V3V4_F_filt.fastq.gz
## Encountered 10050 unique sequences from 27024 total sequences read.
## Dereplicating sequence entries in Fastq file: ./filtered/434V3V4_F_filt.fastq.gz
## Encountered 7155 unique sequences from 26740 total sequences read.
## Dereplicating sequence entries in Fastq file: ./filtered/435V3V4_F_filt.fastq.gz
## Encountered 10463 unique sequences from 36840 total sequences read.
## Dereplicating sequence entries in Fastq file: ./filtered/436V3V4_F_filt.fastq.gz
## Encountered 10862 unique sequences from 45165 total sequences read.
## Dereplicating sequence entries in Fastq file: ./filtered/437V3V4_F_filt.fastq.gz
## Encountered 10654 unique sequences from 29443 total sequences read.
## Dereplicating sequence entries in Fastq file: ./filtered/438V3V4_F_filt.fastq.gz
## Encountered 13661 unique sequences from 48706 total sequences read.
## Dereplicating sequence entries in Fastq file: ./filtered/439V3V4_F_filt.fastq.gz
## Encountered 12633 unique sequences from 41013 total sequences read.
## Dereplicating sequence entries in Fastq file: ./filtered/43V3V4_F_filt.fastq.gz
## Encountered 9526 unique sequences from 40885 total sequences read.
## Dereplicating sequence entries in Fastq file: ./filtered/440V3V4_F_filt.fastq.gz
## Encountered 13153 unique sequences from 47852 total sequences read.
## Dereplicating sequence entries in Fastq file: ./filtered/441V3V4_F_filt.fastq.gz
## Encountered 7989 unique sequences from 25072 total sequences read.
## Dereplicating sequence entries in Fastq file: ./filtered/442V3V4_F_filt.fastq.gz
## Encountered 7204 unique sequences from 22795 total sequences read.
## Dereplicating sequence entries in Fastq file: ./filtered/443V3V4_F_filt.fastq.gz
## Encountered 8772 unique sequences from 26905 total sequences read.
## Dereplicating sequence entries in Fastq file: ./filtered/444V3V4_F_filt.fastq.gz
## Encountered 7315 unique sequences from 29185 total sequences read.
## Dereplicating sequence entries in Fastq file: ./filtered/445V3V4_F_filt.fastq.gz
## Encountered 9244 unique sequences from 24921 total sequences read.
## Dereplicating sequence entries in Fastq file: ./filtered/446V3V4_F_filt.fastq.gz
## Encountered 8550 unique sequences from 30117 total sequences read.
```

```
## Dereplicating sequence entries in Fastq file: ./filtered/447V3V4_F_filt.fastq.gz
## Encountered 11019 unique sequences from 33702 total sequences read.
## Dereplicating sequence entries in Fastq file: ./filtered/448V3V4_F_filt.fastq.gz
## Encountered 4226 unique sequences from 14605 total sequences read.
## Dereplicating sequence entries in Fastq file: ./filtered/449V3V4_F_filt.fastq.gz
## Encountered 9548 unique sequences from 24622 total sequences read.
## Dereplicating sequence entries in Fastq file: ./filtered/450V3V4_F_filt.fastq.gz
## Encountered 7849 unique sequences from 26269 total sequences read.
## Dereplicating sequence entries in Fastq file: ./filtered/451V3V4_F_filt.fastq.gz
## Encountered 10464 unique sequences from 28276 total sequences read.
## Dereplicating sequence entries in Fastq file: ./filtered/452V3V4_F_filt.fastq.gz
## Encountered 9401 unique sequences from 37602 total sequences read.
## Dereplicating sequence entries in Fastq file: ./filtered/453V3V4_F_filt.fastq.gz
## Encountered 12119 unique sequences from 30757 total sequences read.
## Dereplicating sequence entries in Fastq file: ./filtered/454V3V4_F_filt.fastq.gz
## Encountered 9653 unique sequences from 32059 total sequences read.
## Dereplicating sequence entries in Fastq file: ./filtered/455V3V4_F_filt.fastq.gz
## Encountered 14008 unique sequences from 35182 total sequences read.
## Dereplicating sequence entries in Fastq file: ./filtered/456V3V4_F_filt.fastq.gz
## Encountered 12604 unique sequences from 41065 total sequences read.
## Dereplicating sequence entries in Fastq file: ./filtered/457V3V4_F_filt.fastq.gz
## Encountered 11002 unique sequences from 25853 total sequences read.
## Dereplicating sequence entries in Fastq file: ./filtered/458V3V4_F_filt.fastq.gz
## Encountered 8998 unique sequences from 26804 total sequences read.
## Dereplicating sequence entries in Fastq file: ./filtered/459V3V4_F_filt.fastq.gz
## Encountered 10087 unique sequences from 24325 total sequences read.
## Dereplicating sequence entries in Fastq file: ./filtered/460V3V4_F_filt.fastq.gz
## Encountered 13137 unique sequences from 40939 total sequences read.
## Dereplicating sequence entries in Fastq file: ./filtered/461V3V4_F_filt.fastq.gz
## Encountered 10661 unique sequences from 28684 total sequences read.
## Dereplicating sequence entries in Fastq file: ./filtered/462V3V4_F_filt.fastq.gz
## Encountered 9484 unique sequences from 36168 total sequences read.
## Dereplicating sequence entries in Fastq file: ./filtered/463V3V4_F_filt.fastq.gz
## Encountered 11597 unique sequences from 37807 total sequences read.
## Dereplicating sequence entries in Fastq file: ./filtered/46V3V4_F_filt.fastq.gz
## Encountered 13344 unique sequences from 44426 total sequences read.
## Dereplicating sequence entries in Fastq file: ./filtered/49V3V4_F_filt.fastq.gz
```

```
## Encountered 8974 unique sequences from 30604 total sequences read.
## Dereplicating sequence entries in Fastq file: ./filtered/50V3V4_F_filt.fastq.gz
## Encountered 8278 unique sequences from 40847 total sequences read.
## Dereplicating sequence entries in Fastq file: ./filtered/61V3V4_F_filt.fastq.gz
## Encountered 9901 unique sequences from 37184 total sequences read.
## Dereplicating sequence entries in Fastq file: ./filtered/64V3V4_F_filt.fastq.gz
## Encountered 7567 unique sequences from 39922 total sequences read.
## Dereplicating sequence entries in Fastq file: ./filtered/72V3V4_F_filt.fastq.gz
## Encountered 9378 unique sequences from 33786 total sequences read.
## Dereplicating sequence entries in Fastq file: ./filtered/73V3V4_F_filt.fastq.gz
## Encountered 8803 unique sequences from 33683 total sequences read.
## Dereplicating sequence entries in Fastq file: ./filtered/77V3V4_F_filt.fastq.gz
## Encountered 8247 unique sequences from 26849 total sequences read.
## Dereplicating sequence entries in Fastq file: ./filtered/9V3V4_F_filt.fastq.gz
## Encountered 9175 unique sequences from 33857 total sequences read.
derepRs <- derepFastq(filtRs, verbose=TRUE)
## Dereplicating sequence entries in Fastq file: ./filtered/13V3V4_R_filt.fastq.gz
## Encountered 3382 unique sequences from 39830 total sequences read.
## Dereplicating sequence entries in Fastq file: ./filtered/14V3V4_R_filt.fastq.gz
## Encountered 2685 unique sequences from 48601 total sequences read.
## Dereplicating sequence entries in Fastq file: ./filtered/238V3V4_R_filt.fastq.gz
## Encountered 4667 unique sequences from 38897 total sequences read.
## Dereplicating sequence entries in Fastq file: ./filtered/239V3V4_R_filt.fastq.gz
## Encountered 1979 unique sequences from 19134 total sequences read.
## Dereplicating sequence entries in Fastq file: ./filtered/240V3V4_R_filt.fastq.gz
## Encountered 3507 unique sequences from 41533 total sequences read.
## Dereplicating sequence entries in Fastq file: ./filtered/241V3V4_R_filt.fastq.gz
## Encountered 2209 unique sequences from 26986 total sequences read.
## Dereplicating sequence entries in Fastq file: ./filtered/242V3V4_R_filt.fastq.gz
## Encountered 3913 unique sequences from 42421 total sequences read.
## Dereplicating sequence entries in Fastq file: ./filtered/243V3V4_R_filt.fastq.gz
## Encountered 5123 unique sequences from 42944 total sequences read.
## Dereplicating sequence entries in Fastq file: ./filtered/244V3V4_R_filt.fastq.gz
## Encountered 3964 unique sequences from 38448 total sequences read.
## Dereplicating sequence entries in Fastq file: ./filtered/245V3V4_R_filt.fastq.gz
## Encountered 3855 unique sequences from 37369 total sequences read.
## Dereplicating sequence entries in Fastq file: ./filtered/246V3V4_R_filt.fastq.gz
```

```
## Encountered 2585 unique sequences from 25227 total sequences read.
## Dereplicating sequence entries in Fastq file: ./filtered/247V3V4_R_filt.fastq.gz
## Encountered 3360 unique sequences from 29406 total sequences read.
## Dereplicating sequence entries in Fastq file: ./filtered/248V3V4_R_filt.fastq.gz
## Encountered 4443 unique sequences from 52825 total sequences read.
## Dereplicating sequence entries in Fastq file: ./filtered/249V3V4_R_filt.fastq.gz
## Encountered 2899 unique sequences from 27785 total sequences read.
## Dereplicating sequence entries in Fastq file: ./filtered/250V3V4_R_filt.fastq.gz
## Encountered 1661 unique sequences from 19015 total sequences read.
## Dereplicating sequence entries in Fastq file: ./filtered/251V3V4_R_filt.fastq.gz
## Encountered 3615 unique sequences from 33834 total sequences read.
## Dereplicating sequence entries in Fastq file: ./filtered/252V3V4_R_filt.fastq.gz
## Encountered 2964 unique sequences from 30665 total sequences read.
## Dereplicating sequence entries in Fastq file: ./filtered/253V3V4_R_filt.fastq.gz
## Encountered 3540 unique sequences from 24005 total sequences read.
## Dereplicating sequence entries in Fastq file: ./filtered/254V3V4_R_filt.fastq.gz
## Encountered 3402 unique sequences from 36360 total sequences read.
## Dereplicating sequence entries in Fastq file: ./filtered/255V3V4_R_filt.fastq.gz
## Encountered 1960 unique sequences from 22285 total sequences read.
## Dereplicating sequence entries in Fastq file: ./filtered/256V3V4_R_filt.fastq.gz
## Encountered 5074 unique sequences from 44924 total sequences read.
## Dereplicating sequence entries in Fastq file: ./filtered/257V3V4_R_filt.fastq.gz
## Encountered 3250 unique sequences from 24498 total sequences read.
## Dereplicating sequence entries in Fastq file: ./filtered/258V3V4_R_filt.fastq.gz
## Encountered 2636 unique sequences from 30627 total sequences read.
## Dereplicating sequence entries in Fastq file: ./filtered/259V3V4_R_filt.fastq.gz
## Encountered 1877 unique sequences from 32850 total sequences read.
## Dereplicating sequence entries in Fastq file: ./filtered/260V3V4_R_filt.fastq.gz
## Encountered 2433 unique sequences from 25493 total sequences read.
## Dereplicating sequence entries in Fastq file: ./filtered/261V3V4_R_filt.fastq.gz
## Encountered 3821 unique sequences from 31778 total sequences read.
## Dereplicating sequence entries in Fastq file: ./filtered/262V3V4_R_filt.fastq.gz
## Encountered 3027 unique sequences from 28407 total sequences read.
## Dereplicating sequence entries in Fastq file: ./filtered/263V3V4_R_filt.fastq.gz
## Encountered 3650 unique sequences from 34592 total sequences read.
## Dereplicating sequence entries in Fastq file: ./filtered/264V3V4_R_filt.fastq.gz
## Encountered 4101 unique sequences from 39991 total sequences read.
```

```
## Dereplicating sequence entries in Fastq file: ./filtered/265V3V4_R_filt.fastq.gz
## Encountered 2846 unique sequences from 30056 total sequences read.
## Dereplicating sequence entries in Fastq file: ./filtered/266V3V4_R_filt.fastq.gz
## Encountered 5350 unique sequences from 52654 total sequences read.
## Dereplicating sequence entries in Fastq file: ./filtered/267V3V4_R_filt.fastq.gz
## Encountered 5012 unique sequences from 46711 total sequences read.
## Dereplicating sequence entries in Fastq file: ./filtered/268V3V4_R_filt.fastq.gz
## Encountered 4458 unique sequences from 42456 total sequences read.
## Dereplicating sequence entries in Fastq file: ./filtered/269V3V4_R_filt.fastq.gz
## Encountered 2868 unique sequences from 26251 total sequences read.
## Dereplicating sequence entries in Fastq file: ./filtered/26V3V4_R_filt.fastq.gz
## Encountered 3483 unique sequences from 36946 total sequences read.
## Dereplicating sequence entries in Fastq file: ./filtered/270V3V4_R_filt.fastq.gz
## Encountered 2473 unique sequences from 25347 total sequences read.
## Dereplicating sequence entries in Fastq file: ./filtered/271V3V4_R_filt.fastq.gz
## Encountered 1981 unique sequences from 24259 total sequences read.
## Dereplicating sequence entries in Fastq file: ./filtered/272V3V4_R_filt.fastq.gz
## Encountered 3498 unique sequences from 33445 total sequences read.
## Dereplicating sequence entries in Fastq file: ./filtered/273V3V4_R_filt.fastq.gz
## Encountered 3198 unique sequences from 30881 total sequences read.
## Dereplicating sequence entries in Fastq file: ./filtered/274V3V4_R_filt.fastq.gz
## Encountered 4002 unique sequences from 38081 total sequences read.
## Dereplicating sequence entries in Fastq file: ./filtered/275V3V4_R_filt.fastq.gz
## Encountered 3881 unique sequences from 30929 total sequences read.
## Dereplicating sequence entries in Fastq file: ./filtered/276V3V4_R_filt.fastq.gz
## Encountered 2275 unique sequences from 33507 total sequences read.
## Dereplicating sequence entries in Fastq file: ./filtered/277V3V4_R_filt.fastq.gz
## Encountered 3260 unique sequences from 26338 total sequences read.
## Dereplicating sequence entries in Fastq file: ./filtered/278V3V4_R_filt.fastq.gz
## Encountered 3120 unique sequences from 32634 total sequences read.
## Dereplicating sequence entries in Fastq file: ./filtered/279V3V4_R_filt.fastq.gz
## Encountered 1300 unique sequences from 22969 total sequences read.
## Dereplicating sequence entries in Fastq file: ./filtered/280V3V4_R_filt.fastq.gz
## Encountered 3902 unique sequences from 40286 total sequences read.
## Dereplicating sequence entries in Fastq file: ./filtered/281V3V4_R_filt.fastq.gz
## Encountered 2022 unique sequences from 18095 total sequences read.
## Dereplicating sequence entries in Fastq file: ./filtered/282V3V4_R_filt.fastq.gz
```

```
## Encountered 3769 unique sequences from 36096 total sequences read.
## Dereplicating sequence entries in Fastq file: ./filtered/283V3V4_R_filt.fastq.gz
## Encountered 4870 unique sequences from 42277 total sequences read.
## Dereplicating sequence entries in Fastq file: ./filtered/284V3V4_R_filt.fastq.gz
## Encountered 4108 unique sequences from 35734 total sequences read.
## Dereplicating sequence entries in Fastq file: ./filtered/285V3V4_R_filt.fastq.gz
## Encountered 2598 unique sequences from 24235 total sequences read.
## Dereplicating sequence entries in Fastq file: ./filtered/286V3V4_R_filt.fastq.gz
## Encountered 3050 unique sequences from 27178 total sequences read.
## Dereplicating sequence entries in Fastq file: ./filtered/287V3V4_R_filt.fastq.gz
## Encountered 2597 unique sequences from 28172 total sequences read.
## Dereplicating sequence entries in Fastq file: ./filtered/288V3V4_R_filt.fastq.gz
## Encountered 4105 unique sequences from 46704 total sequences read.
## Dereplicating sequence entries in Fastq file: ./filtered/289V3V4_R_filt.fastq.gz
## Encountered 2779 unique sequences from 26921 total sequences read.
## Dereplicating sequence entries in Fastq file: ./filtered/290V3V4_R_filt.fastq.gz
## Encountered 3755 unique sequences from 49196 total sequences read.
## Dereplicating sequence entries in Fastq file: ./filtered/291V3V4_R_filt.fastq.gz
## Encountered 3315 unique sequences from 39459 total sequences read.
## Dereplicating sequence entries in Fastq file: ./filtered/292V3V4_R_filt.fastq.gz
## Encountered 2653 unique sequences from 39888 total sequences read.
## Dereplicating sequence entries in Fastq file: ./filtered/293V3V4_R_filt.fastq.gz
## Encountered 2494 unique sequences from 20013 total sequences read.
## Dereplicating sequence entries in Fastq file: ./filtered/294V3V4_R_filt.fastq.gz
## Encountered 2382 unique sequences from 22293 total sequences read.
## Dereplicating sequence entries in Fastq file: ./filtered/295V3V4_R_filt.fastq.gz
## Encountered 2680 unique sequences from 22913 total sequences read.
## Dereplicating sequence entries in Fastq file: ./filtered/296V3V4_R_filt.fastq.gz
## Encountered 3196 unique sequences from 35872 total sequences read.
## Dereplicating sequence entries in Fastq file: ./filtered/297V3V4_R_filt.fastq.gz
## Encountered 2697 unique sequences from 21174 total sequences read.
## Dereplicating sequence entries in Fastq file: ./filtered/298V3V4_R_filt.fastq.gz
## Encountered 2944 unique sequences from 31913 total sequences read.
## Dereplicating sequence entries in Fastq file: ./filtered/299V3V4_R_filt.fastq.gz
## Encountered 1785 unique sequences from 14555 total sequences read.
## Dereplicating sequence entries in Fastq file: ./filtered/300V3V4_R_filt.fastq.gz
## Encountered 3473 unique sequences from 36219 total sequences read.
```

```
## Dereplicating sequence entries in Fastq file: ./filtered/301V3V4_R_filt.fastq.gz
## Encountered 3904 unique sequences from 42984 total sequences read.
## Dereplicating sequence entries in Fastq file: ./filtered/302V3V4_R_filt.fastq.gz
## Encountered 3516 unique sequences from 31240 total sequences read.
## Dereplicating sequence entries in Fastq file: ./filtered/303V3V4_R_filt.fastq.gz
## Encountered 4691 unique sequences from 38607 total sequences read.
## Dereplicating sequence entries in Fastq file: ./filtered/304V3V4_R_filt.fastq.gz
## Encountered 4116 unique sequences from 37551 total sequences read.
## Dereplicating sequence entries in Fastq file: ./filtered/305V3V4_R_filt.fastq.gz
## Encountered 4832 unique sequences from 37523 total sequences read.
## Dereplicating sequence entries in Fastq file: ./filtered/306V3V4_R_filt.fastq.gz
## Encountered 5226 unique sequences from 48739 total sequences read.
## Dereplicating sequence entries in Fastq file: ./filtered/307V3V4_R_filt.fastq.gz
## Encountered 2876 unique sequences from 20752 total sequences read.
## Dereplicating sequence entries in Fastq file: ./filtered/308V3V4_R_filt.fastq.gz
## Encountered 2435 unique sequences from 28937 total sequences read.
## Dereplicating sequence entries in Fastq file: ./filtered/309V3V4_R_filt.fastq.gz
## Encountered 4003 unique sequences from 32948 total sequences read.
## Dereplicating sequence entries in Fastq file: ./filtered/30V3V4_R_filt.fastq.gz
## Encountered 3710 unique sequences from 39109 total sequences read.
## Dereplicating sequence entries in Fastq file: ./filtered/310V3V4_R_filt.fastq.gz
## Encountered 2557 unique sequences from 30140 total sequences read.
## Dereplicating sequence entries in Fastq file: ./filtered/311V3V4_R_filt.fastq.gz
## Encountered 3615 unique sequences from 34343 total sequences read.
## Dereplicating sequence entries in Fastq file: ./filtered/312V3V4_R_filt.fastq.gz
## Encountered 3958 unique sequences from 45349 total sequences read.
## Dereplicating sequence entries in Fastq file: ./filtered/313V3V4_R_filt.fastq.gz
## Encountered 3311 unique sequences from 35930 total sequences read.
## Dereplicating sequence entries in Fastq file: ./filtered/314V3V4_R_filt.fastq.gz
## Encountered 5155 unique sequences from 48076 total sequences read.
## Dereplicating sequence entries in Fastq file: ./filtered/315V3V4_R_filt.fastq.gz
## Encountered 1739 unique sequences from 19510 total sequences read.
## Dereplicating sequence entries in Fastq file: ./filtered/316V3V4_R_filt.fastq.gz
## Encountered 3118 unique sequences from 34555 total sequences read.
## Dereplicating sequence entries in Fastq file: ./filtered/317V3V4_R_filt.fastq.gz
## Encountered 3277 unique sequences from 28896 total sequences read.
## Dereplicating sequence entries in Fastq file: ./filtered/318V3V4_R_filt.fastq.gz
```

```
## Encountered 1138 unique sequences from 22669 total sequences read.
## Dereplicating sequence entries in Fastq file: ./filtered/319V3V4_R_filt.fastq.gz
## Encountered 2918 unique sequences from 26236 total sequences read.
## Dereplicating sequence entries in Fastq file: ./filtered/320V3V4_R_filt.fastq.gz
## Encountered 4611 unique sequences from 40834 total sequences read.
## Dereplicating sequence entries in Fastq file: ./filtered/321V3V4_R_filt.fastq.gz
## Encountered 4434 unique sequences from 31389 total sequences read.
## Dereplicating sequence entries in Fastq file: ./filtered/322V3V4_R_filt.fastq.gz
## Encountered 3860 unique sequences from 41066 total sequences read.
## Dereplicating sequence entries in Fastq file: ./filtered/323V3V4_R_filt.fastq.gz
## Encountered 3238 unique sequences from 25288 total sequences read.
## Dereplicating sequence entries in Fastq file: ./filtered/324V3V4_R_filt.fastq.gz
## Encountered 2859 unique sequences from 29183 total sequences read.
## Dereplicating sequence entries in Fastq file: ./filtered/325V3V4_R_filt.fastq.gz
## Encountered 4872 unique sequences from 38179 total sequences read.
## Dereplicating sequence entries in Fastq file: ./filtered/326V3V4_R_filt.fastq.gz
## Encountered 3009 unique sequences from 29020 total sequences read.
## Dereplicating sequence entries in Fastq file: ./filtered/327V3V4_R_filt.fastq.gz
## Encountered 4112 unique sequences from 34516 total sequences read.
## Dereplicating sequence entries in Fastq file: ./filtered/328V3V4_R_filt.fastq.gz
## Encountered 1518 unique sequences from 31660 total sequences read.
## Dereplicating sequence entries in Fastq file: ./filtered/329V3V4_R_filt.fastq.gz
## Encountered 4011 unique sequences from 32792 total sequences read.
## Dereplicating sequence entries in Fastq file: ./filtered/330V3V4_R_filt.fastq.gz
## Encountered 3129 unique sequences from 37583 total sequences read.
## Dereplicating sequence entries in Fastq file: ./filtered/331V3V4_R_filt.fastq.gz
## Encountered 2922 unique sequences from 23616 total sequences read.
## Dereplicating sequence entries in Fastq file: ./filtered/332V3V4_R_filt.fastq.gz
## Encountered 3544 unique sequences from 37669 total sequences read.
## Dereplicating sequence entries in Fastq file: ./filtered/333V3V4_R_filt.fastq.gz
## Encountered 3298 unique sequences from 37731 total sequences read.
## Dereplicating sequence entries in Fastq file: ./filtered/334V3V4_R_filt.fastq.gz
## Encountered 2895 unique sequences from 28456 total sequences read.
## Dereplicating sequence entries in Fastq file: ./filtered/335V3V4_R_filt.fastq.gz
## Encountered 3874 unique sequences from 35913 total sequences read.
## Dereplicating sequence entries in Fastq file: ./filtered/336V3V4_R_filt.fastq.gz
## Encountered 3063 unique sequences from 36861 total sequences read.
```

```
## Dereplicating sequence entries in Fastq file: ./filtered/337V3V4_R_filt.fastq.gz
## Encountered 2557 unique sequences from 35569 total sequences read.
## Dereplicating sequence entries in Fastq file: ./filtered/338V3V4_R_filt.fastq.gz
## Encountered 3476 unique sequences from 41625 total sequences read.
## Dereplicating sequence entries in Fastq file: ./filtered/339V3V4_R_filt.fastq.gz
## Encountered 3450 unique sequences from 27782 total sequences read.
## Dereplicating sequence entries in Fastq file: ./filtered/340V3V4_R_filt.fastq.gz
## Encountered 3722 unique sequences from 39916 total sequences read.
## Dereplicating sequence entries in Fastq file: ./filtered/341V3V4_R_filt.fastq.gz
## Encountered 4418 unique sequences from 40216 total sequences read.
## Dereplicating sequence entries in Fastq file: ./filtered/342V3V4_R_filt.fastq.gz
## Encountered 1837 unique sequences from 15582 total sequences read.
## Dereplicating sequence entries in Fastq file: ./filtered/343V3V4_R_filt.fastq.gz
## Encountered 3457 unique sequences from 32544 total sequences read.
## Dereplicating sequence entries in Fastq file: ./filtered/344V3V4_R_filt.fastq.gz
## Encountered 5013 unique sequences from 45852 total sequences read.
## Dereplicating sequence entries in Fastq file: ./filtered/345V3V4_R_filt.fastq.gz
## Encountered 2894 unique sequences from 34702 total sequences read.
## Dereplicating sequence entries in Fastq file: ./filtered/346V3V4_R_filt.fastq.gz
## Encountered 3515 unique sequences from 36982 total sequences read.
## Dereplicating sequence entries in Fastq file: ./filtered/347V3V4_R_filt.fastq.gz
## Encountered 2759 unique sequences from 19274 total sequences read.
## Dereplicating sequence entries in Fastq file: ./filtered/348V3V4_R_filt.fastq.gz
## Encountered 3341 unique sequences from 34428 total sequences read.
## Dereplicating sequence entries in Fastq file: ./filtered/349V3V4_R_filt.fastq.gz
## Encountered 3066 unique sequences from 30679 total sequences read.
## Dereplicating sequence entries in Fastq file: ./filtered/350V3V4_R_filt.fastq.gz
## Encountered 2476 unique sequences from 25346 total sequences read.
## Dereplicating sequence entries in Fastq file: ./filtered/351V3V4_R_filt.fastq.gz
## Encountered 3536 unique sequences from 24485 total sequences read.
## Dereplicating sequence entries in Fastq file: ./filtered/352V3V4_R_filt.fastq.gz
## Encountered 3480 unique sequences from 30542 total sequences read.
## Dereplicating sequence entries in Fastq file: ./filtered/353V3V4_R_filt.fastq.gz
## Encountered 4653 unique sequences from 32027 total sequences read.
## Dereplicating sequence entries in Fastq file: ./filtered/354V3V4_R_filt.fastq.gz
## Encountered 3073 unique sequences from 32743 total sequences read.
## Dereplicating sequence entries in Fastq file: ./filtered/355V3V4_R_filt.fastq.gz
```

```
## Encountered 1851 unique sequences from 18743 total sequences read.
## Dereplicating sequence entries in Fastq file: ./filtered/356V3V4_R_filt.fastq.gz
## Encountered 2646 unique sequences from 33571 total sequences read.
## Dereplicating sequence entries in Fastq file: ./filtered/357V3V4_R_filt.fastq.gz
## Encountered 3194 unique sequences from 34251 total sequences read.
## Dereplicating sequence entries in Fastq file: ./filtered/358V3V4_R_filt.fastq.gz
## Encountered 2462 unique sequences from 28764 total sequences read.
## Dereplicating sequence entries in Fastq file: ./filtered/359V3V4_R_filt.fastq.gz
## Encountered 1853 unique sequences from 31957 total sequences read.
## Dereplicating sequence entries in Fastq file: ./filtered/360V3V4_R_filt.fastq.gz
## Encountered 3342 unique sequences from 41312 total sequences read.
## Dereplicating sequence entries in Fastq file: ./filtered/361V3V4_R_filt.fastq.gz
## Encountered 3560 unique sequences from 38598 total sequences read.
## Dereplicating sequence entries in Fastq file: ./filtered/362V3V4_R_filt.fastq.gz
## Encountered 2870 unique sequences from 35770 total sequences read.
## Dereplicating sequence entries in Fastq file: ./filtered/363V3V4_R_filt.fastq.gz
## Encountered 2800 unique sequences from 23062 total sequences read.
## Dereplicating sequence entries in Fastq file: ./filtered/364V3V4_R_filt.fastq.gz
## Encountered 1609 unique sequences from 14104 total sequences read.
## Dereplicating sequence entries in Fastq file: ./filtered/365V3V4_R_filt.fastq.gz
## Encountered 3652 unique sequences from 29000 total sequences read.
## Dereplicating sequence entries in Fastq file: ./filtered/366V3V4_R_filt.fastq.gz
## Encountered 2240 unique sequences from 27430 total sequences read.
## Dereplicating sequence entries in Fastq file: ./filtered/367V3V4_R_filt.fastq.gz
## Encountered 2104 unique sequences from 29157 total sequences read.
## Dereplicating sequence entries in Fastq file: ./filtered/368V3V4_R_filt.fastq.gz
## Encountered 3611 unique sequences from 29834 total sequences read.
## Dereplicating sequence entries in Fastq file: ./filtered/369V3V4_R_filt.fastq.gz
## Encountered 3212 unique sequences from 32668 total sequences read.
## Dereplicating sequence entries in Fastq file: ./filtered/370V3V4_R_filt.fastq.gz
## Encountered 2362 unique sequences from 33038 total sequences read.
## Dereplicating sequence entries in Fastq file: ./filtered/371V3V4_R_filt.fastq.gz
## Encountered 2935 unique sequences from 22906 total sequences read.
## Dereplicating sequence entries in Fastq file: ./filtered/372V3V4_R_filt.fastq.gz
## Encountered 3249 unique sequences from 33636 total sequences read.
## Dereplicating sequence entries in Fastq file: ./filtered/373V3V4_R_filt.fastq.gz
## Encountered 2927 unique sequences from 30634 total sequences read.
```

```
## Dereplicating sequence entries in Fastq file: ./filtered/374V3V4_R_filt.fastq.gz
## Encountered 2125 unique sequences from 19774 total sequences read.
## Dereplicating sequence entries in Fastq file: ./filtered/375V3V4_R_filt.fastq.gz
## Encountered 2499 unique sequences from 23800 total sequences read.
## Dereplicating sequence entries in Fastq file: ./filtered/376V3V4_R_filt.fastq.gz
## Encountered 3466 unique sequences from 36884 total sequences read.
## Dereplicating sequence entries in Fastq file: ./filtered/377V3V4_R_filt.fastq.gz
## Encountered 4559 unique sequences from 37157 total sequences read.
## Dereplicating sequence entries in Fastq file: ./filtered/378V3V4_R_filt.fastq.gz
## Encountered 2866 unique sequences from 38178 total sequences read.
## Dereplicating sequence entries in Fastq file: ./filtered/379V3V4_R_filt.fastq.gz
## Encountered 3852 unique sequences from 25106 total sequences read.
## Dereplicating sequence entries in Fastq file: ./filtered/380V3V4_R_filt.fastq.gz
## Encountered 3051 unique sequences from 34116 total sequences read.
## Dereplicating sequence entries in Fastq file: ./filtered/381V3V4_R_filt.fastq.gz
## Encountered 3093 unique sequences from 30707 total sequences read.
## Dereplicating sequence entries in Fastq file: ./filtered/382V3V4_R_filt.fastq.gz
## Encountered 2467 unique sequences from 27524 total sequences read.
## Dereplicating sequence entries in Fastq file: ./filtered/383V3V4_R_filt.fastq.gz
## Encountered 2420 unique sequences from 32527 total sequences read.
## Dereplicating sequence entries in Fastq file: ./filtered/384V3V4_R_filt.fastq.gz
## Encountered 3832 unique sequences from 40299 total sequences read.
## Dereplicating sequence entries in Fastq file: ./filtered/385V3V4_R_filt.fastq.gz
## Encountered 4275 unique sequences from 40246 total sequences read.
## Dereplicating sequence entries in Fastq file: ./filtered/386V3V4_R_filt.fastq.gz
## Encountered 3283 unique sequences from 35589 total sequences read.
## Dereplicating sequence entries in Fastq file: ./filtered/387V3V4_R_filt.fastq.gz
## Encountered 1754 unique sequences from 16541 total sequences read.
## Dereplicating sequence entries in Fastq file: ./filtered/388V3V4_R_filt.fastq.gz
## Encountered 1632 unique sequences from 17488 total sequences read.
## Dereplicating sequence entries in Fastq file: ./filtered/389V3V4_R_filt.fastq.gz
## Encountered 3389 unique sequences from 22427 total sequences read.
## Dereplicating sequence entries in Fastq file: ./filtered/38V3V4_R_filt.fastq.gz
## Encountered 5224 unique sequences from 51960 total sequences read.
## Dereplicating sequence entries in Fastq file: ./filtered/390V3V4_R_filt.fastq.gz
## Encountered 1705 unique sequences from 15260 total sequences read.
## Dereplicating sequence entries in Fastq file: ./filtered/391V3V4_R_filt.fastq.gz
```

```
## Encountered 2660 unique sequences from 21125 total sequences read.
## Dereplicating sequence entries in Fastq file: ./filtered/392V3V4_R_filt.fastq.gz
## Encountered 1532 unique sequences from 24778 total sequences read.
## Dereplicating sequence entries in Fastq file: ./filtered/393V3V4_R_filt.fastq.gz
## Encountered 2156 unique sequences from 23560 total sequences read.
## Dereplicating sequence entries in Fastq file: ./filtered/394V3V4_R_filt.fastq.gz
## Encountered 3648 unique sequences from 33363 total sequences read.
## Dereplicating sequence entries in Fastq file: ./filtered/395V3V4_R_filt.fastq.gz
## Encountered 1936 unique sequences from 23249 total sequences read.
## Dereplicating sequence entries in Fastq file: ./filtered/396V3V4_R_filt.fastq.gz
## Encountered 4192 unique sequences from 37426 total sequences read.
## Dereplicating sequence entries in Fastq file: ./filtered/397V3V4_R_filt.fastq.gz
## Encountered 3202 unique sequences from 25788 total sequences read.
## Dereplicating sequence entries in Fastq file: ./filtered/398V3V4_R_filt.fastq.gz
## Encountered 2870 unique sequences from 37756 total sequences read.
## Dereplicating sequence entries in Fastq file: ./filtered/399V3V4_R_filt.fastq.gz
## Encountered 3998 unique sequences from 36360 total sequences read.
## Dereplicating sequence entries in Fastq file: ./filtered/39V3V4_R_filt.fastq.gz
## Encountered 4564 unique sequences from 47704 total sequences read.
## Dereplicating sequence entries in Fastq file: ./filtered/400V3V4_R_filt.fastq.gz
## Encountered 2318 unique sequences from 34959 total sequences read.
## Dereplicating sequence entries in Fastq file: ./filtered/401V3V4_R_filt.fastq.gz
## Encountered 1957 unique sequences from 20870 total sequences read.
## Dereplicating sequence entries in Fastq file: ./filtered/402V3V4_R_filt.fastq.gz
## Encountered 2385 unique sequences from 25325 total sequences read.
## Dereplicating sequence entries in Fastq file: ./filtered/403V3V4_R_filt.fastq.gz
## Encountered 2565 unique sequences from 22182 total sequences read.
## Dereplicating sequence entries in Fastq file: ./filtered/404V3V4_R_filt.fastq.gz
## Encountered 3409 unique sequences from 36937 total sequences read.
## Dereplicating sequence entries in Fastq file: ./filtered/405V3V4_R_filt.fastq.gz
## Encountered 1749 unique sequences from 23136 total sequences read.
## Dereplicating sequence entries in Fastq file: ./filtered/406V3V4_R_filt.fastq.gz
## Encountered 3498 unique sequences from 41498 total sequences read.
## Dereplicating sequence entries in Fastq file: ./filtered/407V3V4_R_filt.fastq.gz
## Encountered 2641 unique sequences from 32112 total sequences read.
## Dereplicating sequence entries in Fastq file: ./filtered/408V3V4_R_filt.fastq.gz
## Encountered 3658 unique sequences from 34875 total sequences read.
```

```
## Dereplicating sequence entries in Fastq file: ./filtered/409V3V4_R_filt.fastq.gz
## Encountered 2154 unique sequences from 24764 total sequences read.
## Dereplicating sequence entries in Fastq file: ./filtered/410V3V4_R_filt.fastq.gz
## Encountered 2975 unique sequences from 31786 total sequences read.
## Dereplicating sequence entries in Fastq file: ./filtered/411V3V4_R_filt.fastq.gz
## Encountered 3900 unique sequences from 31304 total sequences read.
## Dereplicating sequence entries in Fastq file: ./filtered/412V3V4_R_filt.fastq.gz
## Encountered 4250 unique sequences from 37457 total sequences read.
## Dereplicating sequence entries in Fastq file: ./filtered/413V3V4_R_filt.fastq.gz
## Encountered 3075 unique sequences from 26551 total sequences read.
## Dereplicating sequence entries in Fastq file: ./filtered/414V3V4_R_filt.fastq.gz
## Encountered 4223 unique sequences from 48887 total sequences read.
## Dereplicating sequence entries in Fastq file: ./filtered/415V3V4_R_filt.fastq.gz
## Encountered 3304 unique sequences from 34973 total sequences read.
## Dereplicating sequence entries in Fastq file: ./filtered/416V3V4_R_filt.fastq.gz
## Encountered 2328 unique sequences from 31882 total sequences read.
## Dereplicating sequence entries in Fastq file: ./filtered/417V3V4_R_filt.fastq.gz
## Encountered 3894 unique sequences from 24933 total sequences read.
## Dereplicating sequence entries in Fastq file: ./filtered/418V3V4_R_filt.fastq.gz
## Encountered 2542 unique sequences from 27617 total sequences read.
## Dereplicating sequence entries in Fastq file: ./filtered/419V3V4_R_filt.fastq.gz
## Encountered 4066 unique sequences from 31810 total sequences read.
## Dereplicating sequence entries in Fastq file: ./filtered/420V3V4_R_filt.fastq.gz
## Encountered 3014 unique sequences from 30935 total sequences read.
## Dereplicating sequence entries in Fastq file: ./filtered/421V3V4_R_filt.fastq.gz
## Encountered 1870 unique sequences from 16588 total sequences read.
## Dereplicating sequence entries in Fastq file: ./filtered/422V3V4_R_filt.fastq.gz
## Encountered 2584 unique sequences from 34570 total sequences read.
## Dereplicating sequence entries in Fastq file: ./filtered/423V3V4_R_filt.fastq.gz
## Encountered 4750 unique sequences from 35072 total sequences read.
## Dereplicating sequence entries in Fastq file: ./filtered/424V3V4_R_filt.fastq.gz
## Encountered 3377 unique sequences from 39136 total sequences read.
## Dereplicating sequence entries in Fastq file: ./filtered/425V3V4_R_filt.fastq.gz
## Encountered 2602 unique sequences from 21924 total sequences read.
## Dereplicating sequence entries in Fastq file: ./filtered/426V3V4_R_filt.fastq.gz
## Encountered 3003 unique sequences from 28276 total sequences read.
## Dereplicating sequence entries in Fastq file: ./filtered/427V3V4_R_filt.fastq.gz
```

```
## Encountered 2458 unique sequences from 22542 total sequences read.
## Dereplicating sequence entries in Fastq file: ./filtered/428V3V4_R_filt.fastq.gz
## Encountered 2521 unique sequences from 34406 total sequences read.
## Dereplicating sequence entries in Fastq file: ./filtered/429V3V4_R_filt.fastq.gz
## Encountered 3267 unique sequences from 26941 total sequences read.
## Dereplicating sequence entries in Fastq file: ./filtered/430V3V4_R_filt.fastq.gz
## Encountered 2851 unique sequences from 37958 total sequences read.
## Dereplicating sequence entries in Fastq file: ./filtered/431V3V4_R_filt.fastq.gz
## Encountered 3437 unique sequences from 38060 total sequences read.
## Dereplicating sequence entries in Fastq file: ./filtered/432V3V4_R_filt.fastq.gz
## Encountered 3331 unique sequences from 38185 total sequences read.
## Dereplicating sequence entries in Fastq file: ./filtered/433V3V4_R_filt.fastq.gz
## Encountered 2970 unique sequences from 27024 total sequences read.
## Dereplicating sequence entries in Fastq file: ./filtered/434V3V4_R_filt.fastq.gz
## Encountered 2105 unique sequences from 26740 total sequences read.
## Dereplicating sequence entries in Fastq file: ./filtered/435V3V4_R_filt.fastq.gz
## Encountered 2784 unique sequences from 36840 total sequences read.
## Dereplicating sequence entries in Fastq file: ./filtered/436V3V4_R_filt.fastq.gz
## Encountered 3834 unique sequences from 45165 total sequences read.
## Dereplicating sequence entries in Fastq file: ./filtered/437V3V4_R_filt.fastq.gz
## Encountered 3337 unique sequences from 29443 total sequences read.
## Dereplicating sequence entries in Fastq file: ./filtered/438V3V4_R_filt.fastq.gz
## Encountered 4686 unique sequences from 48706 total sequences read.
## Dereplicating sequence entries in Fastq file: ./filtered/439V3V4_R_filt.fastq.gz
## Encountered 3920 unique sequences from 41013 total sequences read.
## Dereplicating sequence entries in Fastq file: ./filtered/43V3V4_R_filt.fastq.gz
## Encountered 3302 unique sequences from 40885 total sequences read.
## Dereplicating sequence entries in Fastq file: ./filtered/440V3V4_R_filt.fastq.gz
## Encountered 4475 unique sequences from 47852 total sequences read.
## Dereplicating sequence entries in Fastq file: ./filtered/441V3V4_R_filt.fastq.gz
## Encountered 2884 unique sequences from 25072 total sequences read.
## Dereplicating sequence entries in Fastq file: ./filtered/442V3V4_R_filt.fastq.gz
## Encountered 2636 unique sequences from 22795 total sequences read.
## Dereplicating sequence entries in Fastq file: ./filtered/443V3V4_R_filt.fastq.gz
## Encountered 3132 unique sequences from 26905 total sequences read.
## Dereplicating sequence entries in Fastq file: ./filtered/444V3V4_R_filt.fastq.gz
## Encountered 2040 unique sequences from 29185 total sequences read.
```

```
## Dereplicating sequence entries in Fastq file: ./filtered/445V3V4_R_filt.fastq.gz
## Encountered 3333 unique sequences from 24921 total sequences read.
## Dereplicating sequence entries in Fastq file: ./filtered/446V3V4_R_filt.fastq.gz
## Encountered 3018 unique sequences from 30117 total sequences read.
## Dereplicating sequence entries in Fastq file: ./filtered/447V3V4_R_filt.fastq.gz
## Encountered 3155 unique sequences from 33702 total sequences read.
## Dereplicating sequence entries in Fastq file: ./filtered/448V3V4_R_filt.fastq.gz
## Encountered 1620 unique sequences from 14605 total sequences read.
## Dereplicating sequence entries in Fastq file: ./filtered/449V3V4_R_filt.fastq.gz
## Encountered 2536 unique sequences from 24622 total sequences read.
## Dereplicating sequence entries in Fastq file: ./filtered/450V3V4_R_filt.fastq.gz
## Encountered 2887 unique sequences from 26269 total sequences read.
## Dereplicating sequence entries in Fastq file: ./filtered/451V3V4_R_filt.fastq.gz
## Encountered 3236 unique sequences from 28276 total sequences read.
## Dereplicating sequence entries in Fastq file: ./filtered/452V3V4_R_filt.fastq.gz
## Encountered 2613 unique sequences from 37602 total sequences read.
## Dereplicating sequence entries in Fastq file: ./filtered/453V3V4_R_filt.fastq.gz
## Encountered 3778 unique sequences from 30757 total sequences read.
## Dereplicating sequence entries in Fastq file: ./filtered/454V3V4_R_filt.fastq.gz
## Encountered 3732 unique sequences from 32059 total sequences read.
## Dereplicating sequence entries in Fastq file: ./filtered/455V3V4_R_filt.fastq.gz
## Encountered 5478 unique sequences from 35182 total sequences read.
## Dereplicating sequence entries in Fastq file: ./filtered/456V3V4_R_filt.fastq.gz
## Encountered 4859 unique sequences from 41065 total sequences read.
## Dereplicating sequence entries in Fastq file: ./filtered/457V3V4_R_filt.fastq.gz
## Encountered 3785 unique sequences from 25853 total sequences read.
## Dereplicating sequence entries in Fastq file: ./filtered/458V3V4_R_filt.fastq.gz
## Encountered 2933 unique sequences from 26804 total sequences read.
## Dereplicating sequence entries in Fastq file: ./filtered/459V3V4_R_filt.fastq.gz
## Encountered 3078 unique sequences from 24325 total sequences read.
## Dereplicating sequence entries in Fastq file: ./filtered/460V3V4_R_filt.fastq.gz
## Encountered 4560 unique sequences from 40939 total sequences read.
## Dereplicating sequence entries in Fastq file: ./filtered/461V3V4_R_filt.fastq.gz
## Encountered 3185 unique sequences from 28684 total sequences read.
## Dereplicating sequence entries in Fastq file: ./filtered/462V3V4_R_filt.fastq.gz
## Encountered 2158 unique sequences from 36168 total sequences read.
## Dereplicating sequence entries in Fastq file: ./filtered/463V3V4_R_filt.fastq.gz
```

```
## Encountered 3948 unique sequences from 37807 total sequences read.

## Dereplicating sequence entries in Fastq file: ./filtered/46V3V4_R_filt.fastq.gz

## Encountered 6311 unique sequences from 44426 total sequences read.

## Dereplicating sequence entries in Fastq file: ./filtered/49V3V4_R_filt.fastq.gz

## Encountered 3650 unique sequences from 30604 total sequences read.

## Dereplicating sequence entries in Fastq file: ./filtered/50V3V4_R_filt.fastq.gz

## Encountered 3885 unique sequences from 40847 total sequences read.

## Dereplicating sequence entries in Fastq file: ./filtered/61V3V4_R_filt.fastq.gz

## Encountered 4077 unique sequences from 37184 total sequences read.

## Dereplicating sequence entries in Fastq file: ./filtered/64V3V4_R_filt.fastq.gz

## Encountered 3431 unique sequences from 39922 total sequences read.

## Dereplicating sequence entries in Fastq file: ./filtered/72V3V4_R_filt.fastq.gz

## Encountered 4750 unique sequences from 33786 total sequences read.

## Dereplicating sequence entries in Fastq file: ./filtered/73V3V4_R_filt.fastq.gz

## Encountered 3833 unique sequences from 33683 total sequences read.

## Dereplicating sequence entries in Fastq file: ./filtered/77V3V4_R_filt.fastq.gz

## Encountered 2962 unique sequences from 26849 total sequences read.

## Dereplicating sequence entries in Fastq file: ./filtered/9V3V4_R_filt.fastq.gz

## Encountered 3380 unique sequences from 33857 total sequences read.

# Name the derep-class objects by the sample names

names(derepFs) <- sample.names

names(derepRs) <- sample.names


errF <- learnErrors(derepFs, multithread = 20)

## 102993376 total bases in 338794 reads from 9 samples will be used for learning the
error rates.

errR <- learnErrors(derepRs, multithread = 20)

## 102224460 total bases in 655285 reads from 19 samples will be used for learning the
error rates.

#I will keep samples pooled for the moment, implying a common error structure to all t
he samples

dadaFs <- dada(derepFs, err=errF, selfConsist = TRUE, multithread = 20)

## selfConsist step 1 ................................................................
......................................................................................
......................................................................................
......

##    selfConsist step 2

##    selfConsist step 3

##    selfConsist step 4
```

```
##    selfConsist step 5

##    selfConsist step 6

## Convergence after  6  rounds.

dadaRs <- dada(derepRs, err=errR, selfConsist = TRUE, multithread = 20)

## selfConsist step 1 ...................................................................
......................................................................................
......................................................................................
......

##    selfConsist step 2

##    selfConsist step 3

##    selfConsist step 4

##    selfConsist step 5

##    selfConsist step 6

## Convergence after  6  rounds.

###

mergers <- mergePairs(dadaFs, derepFs, dadaRs, derepRs, verbose=TRUE)

## 39286 paired-reads (in 389 unique pairings) successfully merged out of 39456 (in 48
7 pairings) input.

## 48317 paired-reads (in 133 unique pairings) successfully merged out of 48396 (in 17
2 pairings) input.

## 36732 paired-reads (in 525 unique pairings) successfully merged out of 37919 (in 92
3 pairings) input.

## 18550 paired-reads (in 100 unique pairings) successfully merged out of 18870 (in 16
7 pairings) input.

## 40551 paired-reads (in 430 unique pairings) successfully merged out of 41043 (in 53
2 pairings) input.

## 26193 paired-reads (in 319 unique pairings) successfully merged out of 26525 (in 41
7 pairings) input.

## 40835 paired-reads (in 454 unique pairings) successfully merged out of 41934 (in 63
8 pairings) input.

## 40254 paired-reads (in 657 unique pairings) successfully merged out of 41904 (in 10
78 pairings) input.

## 35871 paired-reads (in 447 unique pairings) successfully merged out of 37851 (in 71
9 pairings) input.

## 35535 paired-reads (in 504 unique pairings) successfully merged out of 36581 (in 83
0 pairings) input.

## 24189 paired-reads (in 314 unique pairings) successfully merged out of 24709 (in 44
2 pairings) input.

## 27716 paired-reads (in 361 unique pairings) successfully merged out of 28690 (in 55
4 pairings) input.

## 51413 paired-reads (in 495 unique pairings) successfully merged out of 52351 (in 75
4 pairings) input.

## 26172 paired-reads (in 265 unique pairings) successfully merged out of 27099 (in 45
9 pairings) input.
```

```
## 18517 paired-reads (in 56 unique pairings) successfully merged out of 18857 (in 81
pairings) input.

## 32448 paired-reads (in 365 unique pairings) successfully merged out of 33297 (in 52
4 pairings) input.

## 29351 paired-reads (in 308 unique pairings) successfully merged out of 30020 (in 43
1 pairings) input.

## 22193 paired-reads (in 425 unique pairings) successfully merged out of 23079 (in 68
5 pairings) input.

## 34922 paired-reads (in 446 unique pairings) successfully merged out of 35766 (in 67
0 pairings) input.

## 21172 paired-reads (in 143 unique pairings) successfully merged out of 21879 (in 22
7 pairings) input.

## 43599 paired-reads (in 354 unique pairings) successfully merged out of 44313 (in 54
1 pairings) input.

## 22534 paired-reads (in 353 unique pairings) successfully merged out of 23606 (in 64
4 pairings) input.

## 29795 paired-reads (in 292 unique pairings) successfully merged out of 30355 (in 38
8 pairings) input.

## 30912 paired-reads (in 97 unique pairings) successfully merged out of 32729 (in 158
pairings) input.

## 21876 paired-reads (in 161 unique pairings) successfully merged out of 24891 (in 28
5 pairings) input.

## 29591 paired-reads (in 518 unique pairings) successfully merged out of 30965 (in 84
4 pairings) input.

## 27033 paired-reads (in 314 unique pairings) successfully merged out of 27772 (in 46
1 pairings) input.

## 32604 paired-reads (in 486 unique pairings) successfully merged out of 34096 (in 78
0 pairings) input.

## 36214 paired-reads (in 420 unique pairings) successfully merged out of 39075 (in 69
7 pairings) input.

## 28708 paired-reads (in 428 unique pairings) successfully merged out of 29591 (in 62
8 pairings) input.

## 50444 paired-reads (in 568 unique pairings) successfully merged out of 52013 (in 99
5 pairings) input.

## 44241 paired-reads (in 614 unique pairings) successfully merged out of 45742 (in 10
38 pairings) input.

## 40520 paired-reads (in 494 unique pairings) successfully merged out of 41738 (in 77
4 pairings) input.

## 25020 paired-reads (in 370 unique pairings) successfully merged out of 25726 (in 52
3 pairings) input.

## 35832 paired-reads (in 467 unique pairings) successfully merged out of 36494 (in 65
8 pairings) input.

## 24527 paired-reads (in 195 unique pairings) successfully merged out of 24974 (in 26
7 pairings) input.

## 22437 paired-reads (in 164 unique pairings) successfully merged out of 23917 (in 27
6 pairings) input.
```

```
## 30669 paired-reads (in 312 unique pairings) successfully merged out of 32768 (in 53
6 pairings) input.

## 29243 paired-reads (in 389 unique pairings) successfully merged out of 30219 (in 59
9 pairings) input.

## 36046 paired-reads (in 430 unique pairings) successfully merged out of 37377 (in 76
5 pairings) input.

## 28611 paired-reads (in 423 unique pairings) successfully merged out of 30019 (in 71
7 pairings) input.

## 31228 paired-reads (in 173 unique pairings) successfully merged out of 32728 (in 27
4 pairings) input.

## 24426 paired-reads (in 409 unique pairings) successfully merged out of 25664 (in 67
2 pairings) input.

## 30803 paired-reads (in 413 unique pairings) successfully merged out of 32264 (in 69
7 pairings) input.

## 21911 paired-reads (in 68 unique pairings) successfully merged out of 22583 (in 108
pairings) input.

## 38504 paired-reads (in 428 unique pairings) successfully merged out of 39574 (in 66
2 pairings) input.

## 16838 paired-reads (in 150 unique pairings) successfully merged out of 17577 (in 27
8 pairings) input.

## 34098 paired-reads (in 329 unique pairings) successfully merged out of 35377 (in 55
7 pairings) input.

## 40109 paired-reads (in 452 unique pairings) successfully merged out of 41571 (in 75
2 pairings) input.

## 33594 paired-reads (in 373 unique pairings) successfully merged out of 35050 (in 71
3 pairings) input.

## 22559 paired-reads (in 235 unique pairings) successfully merged out of 23712 (in 43
1 pairings) input.

## 24328 paired-reads (in 288 unique pairings) successfully merged out of 26545 (in 59
3 pairings) input.

## 27093 paired-reads (in 204 unique pairings) successfully merged out of 27642 (in 33
8 pairings) input.

## 44514 paired-reads (in 475 unique pairings) successfully merged out of 45956 (in 71
6 pairings) input.

## 25680 paired-reads (in 388 unique pairings) successfully merged out of 26165 (in 56
1 pairings) input.

## 47795 paired-reads (in 485 unique pairings) successfully merged out of 48780 (in 60
2 pairings) input.

## 37850 paired-reads (in 213 unique pairings) successfully merged out of 38964 (in 30
7 pairings) input.

## 38023 paired-reads (in 268 unique pairings) successfully merged out of 39515 (in 37
9 pairings) input.

## 18904 paired-reads (in 251 unique pairings) successfully merged out of 19471 (in 37
7 pairings) input.

## 21288 paired-reads (in 277 unique pairings) successfully merged out of 21837 (in 38
0 pairings) input.
```

```
## 21804 paired-reads (in 220 unique pairings) successfully merged out of 22509 (in 32
9 pairings) input.

## 34303 paired-reads (in 355 unique pairings) successfully merged out of 35270 (in 49
3 pairings) input.

## 19857 paired-reads (in 362 unique pairings) successfully merged out of 20548 (in 56
5 pairings) input.

## 30654 paired-reads (in 304 unique pairings) successfully merged out of 31392 (in 46
4 pairings) input.

## 13669 paired-reads (in 138 unique pairings) successfully merged out of 14014 (in 26
5 pairings) input.

## 34587 paired-reads (in 415 unique pairings) successfully merged out of 35617 (in 60
9 pairings) input.

## 40537 paired-reads (in 456 unique pairings) successfully merged out of 42406 (in 75
7 pairings) input.

## 29740 paired-reads (in 364 unique pairings) successfully merged out of 30438 (in 61
8 pairings) input.

## 35971 paired-reads (in 616 unique pairings) successfully merged out of 37296 (in 10
20 pairings) input.

## 36071 paired-reads (in 661 unique pairings) successfully merged out of 36957 (in 92
0 pairings) input.

## 35341 paired-reads (in 555 unique pairings) successfully merged out of 36534 (in 91
2 pairings) input.

## 45930 paired-reads (in 873 unique pairings) successfully merged out of 47534 (in 13
21 pairings) input.

## 19265 paired-reads (in 374 unique pairings) successfully merged out of 19967 (in 57
8 pairings) input.

## 26496 paired-reads (in 206 unique pairings) successfully merged out of 28605 (in 36
1 pairings) input.

## 30762 paired-reads (in 340 unique pairings) successfully merged out of 32285 (in 54
7 pairings) input.

## 38314 paired-reads (in 272 unique pairings) successfully merged out of 38708 (in 42
1 pairings) input.

## 28869 paired-reads (in 258 unique pairings) successfully merged out of 29723 (in 35
1 pairings) input.

## 32494 paired-reads (in 405 unique pairings) successfully merged out of 33619 (in 64
2 pairings) input.

## 43617 paired-reads (in 347 unique pairings) successfully merged out of 44876 (in 52
5 pairings) input.

## 32559 paired-reads (in 263 unique pairings) successfully merged out of 35199 (in 46
0 pairings) input.

## 45271 paired-reads (in 699 unique pairings) successfully merged out of 46991 (in 12
04 pairings) input.

## 18593 paired-reads (in 146 unique pairings) successfully merged out of 19115 (in 21
1 pairings) input.

## 33275 paired-reads (in 442 unique pairings) successfully merged out of 34094 (in 61
8 pairings) input.
```

```
## 27605 paired-reads (in 329 unique pairings) successfully merged out of 28249 (in 47
1 pairings) input.

## 22031 paired-reads (in 49 unique pairings) successfully merged out of 22553 (in 67
pairings) input.

## 25045 paired-reads (in 337 unique pairings) successfully merged out of 25756 (in 47
3 pairings) input.

## 38752 paired-reads (in 634 unique pairings) successfully merged out of 39828 (in 93
0 pairings) input.

## 29280 paired-reads (in 540 unique pairings) successfully merged out of 30359 (in 87
5 pairings) input.

## 39137 paired-reads (in 579 unique pairings) successfully merged out of 40446 (in 84
0 pairings) input.

## 23841 paired-reads (in 494 unique pairings) successfully merged out of 24735 (in 74
3 pairings) input.

## 27483 paired-reads (in 227 unique pairings) successfully merged out of 28683 (in 41
0 pairings) input.

## 36237 paired-reads (in 532 unique pairings) successfully merged out of 37178 (in 96
6 pairings) input.

## 27850 paired-reads (in 278 unique pairings) successfully merged out of 28428 (in 45
8 pairings) input.

## 32802 paired-reads (in 498 unique pairings) successfully merged out of 33614 (in 78
4 pairings) input.

## 29932 paired-reads (in 64 unique pairings) successfully merged out of 31489 (in 82
pairings) input.

## 30106 paired-reads (in 407 unique pairings) successfully merged out of 31897 (in 68
5 pairings) input.

## 36038 paired-reads (in 370 unique pairings) successfully merged out of 36932 (in 51
0 pairings) input.

## 22305 paired-reads (in 261 unique pairings) successfully merged out of 22843 (in 41
3 pairings) input.

## 36088 paired-reads (in 373 unique pairings) successfully merged out of 37150 (in 56
0 pairings) input.

## 36021 paired-reads (in 248 unique pairings) successfully merged out of 37083 (in 40
7 pairings) input.

## 27081 paired-reads (in 300 unique pairings) successfully merged out of 27890 (in 46
3 pairings) input.

## 34361 paired-reads (in 337 unique pairings) successfully merged out of 35150 (in 51
1 pairings) input.

## 35630 paired-reads (in 250 unique pairings) successfully merged out of 36461 (in 33
8 pairings) input.

## 33077 paired-reads (in 227 unique pairings) successfully merged out of 35291 (in 37
9 pairings) input.

## 40151 paired-reads (in 301 unique pairings) successfully merged out of 41006 (in 45
9 pairings) input.

## 26343 paired-reads (in 475 unique pairings) successfully merged out of 27190 (in 71
2 pairings) input.
```

```
## 37698 paired-reads (in 474 unique pairings) successfully merged out of 39426 (in 82
0 pairings) input.

## 37969 paired-reads (in 558 unique pairings) successfully merged out of 39423 (in 92
4 pairings) input.

## 15029 paired-reads (in 182 unique pairings) successfully merged out of 15169 (in 23
8 pairings) input.

## 30824 paired-reads (in 323 unique pairings) successfully merged out of 31904 (in 53
2 pairings) input.

## 43030 paired-reads (in 524 unique pairings) successfully merged out of 45060 (in 92
6 pairings) input.

## 31752 paired-reads (in 178 unique pairings) successfully merged out of 34399 (in 32
5 pairings) input.

## 34928 paired-reads (in 329 unique pairings) successfully merged out of 36291 (in 55
0 pairings) input.

## 17790 paired-reads (in 314 unique pairings) successfully merged out of 18667 (in 57
3 pairings) input.

## 33172 paired-reads (in 336 unique pairings) successfully merged out of 33966 (in 54
3 pairings) input.

## 29607 paired-reads (in 308 unique pairings) successfully merged out of 30335 (in 44
6 pairings) input.

## 24446 paired-reads (in 197 unique pairings) successfully merged out of 25075 (in 28
3 pairings) input.

## 23016 paired-reads (in 310 unique pairings) successfully merged out of 23531 (in 49
8 pairings) input.

## 29118 paired-reads (in 262 unique pairings) successfully merged out of 29859 (in 41
0 pairings) input.

## 29663 paired-reads (in 587 unique pairings) successfully merged out of 30985 (in 95
6 pairings) input.

## 31485 paired-reads (in 358 unique pairings) successfully merged out of 32131 (in 48
3 pairings) input.

## 18107 paired-reads (in 199 unique pairings) successfully merged out of 18417 (in 30
1 pairings) input.

## 32589 paired-reads (in 305 unique pairings) successfully merged out of 33185 (in 43
0 pairings) input.

## 31963 paired-reads (in 321 unique pairings) successfully merged out of 33503 (in 49
7 pairings) input.

## 27838 paired-reads (in 256 unique pairings) successfully merged out of 28251 (in 33
4 pairings) input.

## 30400 paired-reads (in 85 unique pairings) successfully merged out of 31728 (in 122
pairings) input.

## 40005 paired-reads (in 240 unique pairings) successfully merged out of 41019 (in 35
5 pairings) input.

## 36156 paired-reads (in 310 unique pairings) successfully merged out of 37809 (in 51
3 pairings) input.

## 34558 paired-reads (in 329 unique pairings) successfully merged out of 35227 (in 43
7 pairings) input.
```

```
## 21770 paired-reads (in 367 unique pairings) successfully merged out of 22552 (in 55
7 pairings) input.

## 13546 paired-reads (in 124 unique pairings) successfully merged out of 13657 (in 17
0 pairings) input.

## 27505 paired-reads (in 331 unique pairings) successfully merged out of 28072 (in 51
9 pairings) input.

## 26936 paired-reads (in 269 unique pairings) successfully merged out of 27092 (in 34
8 pairings) input.

## 28224 paired-reads (in 189 unique pairings) successfully merged out of 28936 (in 25
9 pairings) input.

## 28398 paired-reads (in 327 unique pairings) successfully merged out of 29150 (in 52
5 pairings) input.

## 31026 paired-reads (in 285 unique pairings) successfully merged out of 32168 (in 44
6 pairings) input.

## 31928 paired-reads (in 192 unique pairings) successfully merged out of 32753 (in 28
3 pairings) input.

## 21847 paired-reads (in 321 unique pairings) successfully merged out of 22334 (in 46
7 pairings) input.

## 32762 paired-reads (in 469 unique pairings) successfully merged out of 33208 (in 60
0 pairings) input.

## 29169 paired-reads (in 351 unique pairings) successfully merged out of 30273 (in 49
8 pairings) input.

## 19316 paired-reads (in 258 unique pairings) successfully merged out of 19478 (in 35
6 pairings) input.

## 22742 paired-reads (in 230 unique pairings) successfully merged out of 23397 (in 34
5 pairings) input.

## 35914 paired-reads (in 460 unique pairings) successfully merged out of 36359 (in 61
7 pairings) input.

## 35202 paired-reads (in 718 unique pairings) successfully merged out of 36264 (in 11
08 pairings) input.

## 36729 paired-reads (in 204 unique pairings) successfully merged out of 37728 (in 28
3 pairings) input.

## 22792 paired-reads (in 388 unique pairings) successfully merged out of 23868 (in 77
9 pairings) input.

## 33051 paired-reads (in 344 unique pairings) successfully merged out of 33683 (in 48
1 pairings) input.

## 29274 paired-reads (in 226 unique pairings) successfully merged out of 30235 (in 35
9 pairings) input.

## 26449 paired-reads (in 276 unique pairings) successfully merged out of 26990 (in 39
1 pairings) input.

## 30725 paired-reads (in 153 unique pairings) successfully merged out of 32202 (in 23
8 pairings) input.

## 38835 paired-reads (in 235 unique pairings) successfully merged out of 39934 (in 37
3 pairings) input.

## 38502 paired-reads (in 398 unique pairings) successfully merged out of 39728 (in 63
0 pairings) input.
```

```
## 34114 paired-reads (in 348 unique pairings) successfully merged out of 35090 (in 57
7 pairings) input.

## 15715 paired-reads (in 151 unique pairings) successfully merged out of 16234 (in 22
7 pairings) input.

## 15781 paired-reads (in 123 unique pairings) successfully merged out of 17245 (in 22
5 pairings) input.

## 20418 paired-reads (in 309 unique pairings) successfully merged out of 21179 (in 55
9 pairings) input.

## 50612 paired-reads (in 540 unique pairings) successfully merged out of 51214 (in 79
4 pairings) input.

## 14057 paired-reads (in 139 unique pairings) successfully merged out of 14936 (in 21
6 pairings) input.

## 19607 paired-reads (in 217 unique pairings) successfully merged out of 20452 (in 42
8 pairings) input.

## 23315 paired-reads (in 108 unique pairings) successfully merged out of 24384 (in 14
7 pairings) input.

## 22565 paired-reads (in 225 unique pairings) successfully merged out of 23217 (in 31
0 pairings) input.

## 31981 paired-reads (in 525 unique pairings) successfully merged out of 32762 (in 70
1 pairings) input.

## 22232 paired-reads (in 140 unique pairings) successfully merged out of 22929 (in 21
2 pairings) input.

## 35870 paired-reads (in 409 unique pairings) successfully merged out of 36743 (in 66
7 pairings) input.

## 23728 paired-reads (in 353 unique pairings) successfully merged out of 24675 (in 59
1 pairings) input.

## 36004 paired-reads (in 275 unique pairings) successfully merged out of 37464 (in 39
8 pairings) input.

## 34882 paired-reads (in 534 unique pairings) successfully merged out of 35724 (in 77
5 pairings) input.

## 46644 paired-reads (in 392 unique pairings) successfully merged out of 47129 (in 60
8 pairings) input.

## 33278 paired-reads (in 151 unique pairings) successfully merged out of 34624 (in 21
5 pairings) input.

## 19917 paired-reads (in 247 unique pairings) successfully merged out of 20521 (in 35
4 pairings) input.

## 24441 paired-reads (in 216 unique pairings) successfully merged out of 25018 (in 33
4 pairings) input.

## 21026 paired-reads (in 233 unique pairings) successfully merged out of 21700 (in 36
2 pairings) input.

## 35862 paired-reads (in 534 unique pairings) successfully merged out of 36565 (in 63
8 pairings) input.

## 22276 paired-reads (in 143 unique pairings) successfully merged out of 22607 (in 21
5 pairings) input.

## 39929 paired-reads (in 242 unique pairings) successfully merged out of 41000 (in 39
8 pairings) input.
```

```
## 30851 paired-reads (in 343 unique pairings) successfully merged out of 31710 (in 46
3 pairings) input.

## 33363 paired-reads (in 405 unique pairings) successfully merged out of 34234 (in 62
8 pairings) input.

## 23795 paired-reads (in 220 unique pairings) successfully merged out of 24497 (in 32
2 pairings) input.

## 30290 paired-reads (in 254 unique pairings) successfully merged out of 31227 (in 45
8 pairings) input.

## 29532 paired-reads (in 311 unique pairings) successfully merged out of 30169 (in 59
2 pairings) input.

## 35642 paired-reads (in 354 unique pairings) successfully merged out of 36976 (in 66
0 pairings) input.

## 25295 paired-reads (in 484 unique pairings) successfully merged out of 25850 (in 68
0 pairings) input.

## 47062 paired-reads (in 358 unique pairings) successfully merged out of 48538 (in 56
2 pairings) input.

## 32614 paired-reads (in 250 unique pairings) successfully merged out of 34437 (in 41
3 pairings) input.

## 30084 paired-reads (in 144 unique pairings) successfully merged out of 31438 (in 22
4 pairings) input.

## 22659 paired-reads (in 325 unique pairings) successfully merged out of 23322 (in 60
8 pairings) input.

## 26615 paired-reads (in 183 unique pairings) successfully merged out of 27224 (in 29
0 pairings) input.

## 29637 paired-reads (in 373 unique pairings) successfully merged out of 30789 (in 76
5 pairings) input.

## 29978 paired-reads (in 235 unique pairings) successfully merged out of 30474 (in 31
3 pairings) input.

## 15671 paired-reads (in 121 unique pairings) successfully merged out of 16088 (in 18
0 pairings) input.

## 32762 paired-reads (in 161 unique pairings) successfully merged out of 34253 (in 26
6 pairings) input.

## 32954 paired-reads (in 563 unique pairings) successfully merged out of 34060 (in 99
3 pairings) input.

## 38047 paired-reads (in 544 unique pairings) successfully merged out of 38827 (in 71
7 pairings) input.

## 20659 paired-reads (in 227 unique pairings) successfully merged out of 21449 (in 39
2 pairings) input.

## 27443 paired-reads (in 256 unique pairings) successfully merged out of 27864 (in 40
1 pairings) input.

## 21470 paired-reads (in 194 unique pairings) successfully merged out of 22005 (in 32
7 pairings) input.

## 33142 paired-reads (in 246 unique pairings) successfully merged out of 34105 (in 34
1 pairings) input.

## 25432 paired-reads (in 363 unique pairings) successfully merged out of 26184 (in 61
0 pairings) input.
```

```
## 36748 paired-reads (in 278 unique pairings) successfully merged out of 37729 (in 33
8 pairings) input.

## 34558 paired-reads (in 316 unique pairings) successfully merged out of 37334 (in 53
5 pairings) input.

## 36651 paired-reads (in 271 unique pairings) successfully merged out of 37449 (in 42
7 pairings) input.

## 25928 paired-reads (in 305 unique pairings) successfully merged out of 26449 (in 51
2 pairings) input.

## 25913 paired-reads (in 194 unique pairings) successfully merged out of 26426 (in 24
3 pairings) input.

## 35563 paired-reads (in 210 unique pairings) successfully merged out of 36436 (in 34
9 pairings) input.

## 43629 paired-reads (in 291 unique pairings) successfully merged out of 44787 (in 42
8 pairings) input.

## 27937 paired-reads (in 371 unique pairings) successfully merged out of 28472 (in 61
6 pairings) input.

## 46301 paired-reads (in 439 unique pairings) successfully merged out of 47887 (in 70
9 pairings) input.

## 38613 paired-reads (in 570 unique pairings) successfully merged out of 40476 (in 84
8 pairings) input.

## 40380 paired-reads (in 571 unique pairings) successfully merged out of 40587 (in 69
3 pairings) input.

## 45987 paired-reads (in 432 unique pairings) successfully merged out of 47315 (in 69
1 pairings) input.

## 23852 paired-reads (in 318 unique pairings) successfully merged out of 24586 (in 45
9 pairings) input.

## 21858 paired-reads (in 246 unique pairings) successfully merged out of 22295 (in 37
3 pairings) input.

## 25373 paired-reads (in 269 unique pairings) successfully merged out of 26311 (in 49
3 pairings) input.

## 27816 paired-reads (in 121 unique pairings) successfully merged out of 28892 (in 15
7 pairings) input.

## 23174 paired-reads (in 387 unique pairings) successfully merged out of 24099 (in 69
4 pairings) input.

## 28592 paired-reads (in 249 unique pairings) successfully merged out of 29614 (in 41
7 pairings) input.

## 31676 paired-reads (in 222 unique pairings) successfully merged out of 33287 (in 42
8 pairings) input.

## 14340 paired-reads (in 80 unique pairings) successfully merged out of 14450 (in 97
pairings) input.

## 23407 paired-reads (in 203 unique pairings) successfully merged out of 24152 (in 31
3 pairings) input.

## 25237 paired-reads (in 175 unique pairings) successfully merged out of 25795 (in 27
3 pairings) input.

## 27127 paired-reads (in 345 unique pairings) successfully merged out of 27823 (in 51
1 pairings) input.
```

```
## 36172 paired-reads (in 217 unique pairings) successfully merged out of 37406 (in 28
2 pairings) input.

## 29043 paired-reads (in 345 unique pairings) successfully merged out of 29584 (in 60
7 pairings) input.

## 30334 paired-reads (in 172 unique pairings) successfully merged out of 31506 (in 29
1 pairings) input.

## 32789 paired-reads (in 593 unique pairings) successfully merged out of 33727 (in 10
33 pairings) input.

## 38792 paired-reads (in 449 unique pairings) successfully merged out of 40206 (in 77
7 pairings) input.

## 24025 paired-reads (in 386 unique pairings) successfully merged out of 24688 (in 71
0 pairings) input.

## 26012 paired-reads (in 177 unique pairings) successfully merged out of 26211 (in 28
5 pairings) input.

## 22544 paired-reads (in 260 unique pairings) successfully merged out of 23555 (in 46
0 pairings) input.

## 39274 paired-reads (in 434 unique pairings) successfully merged out of 40165 (in 68
6 pairings) input.

## 26615 paired-reads (in 363 unique pairings) successfully merged out of 27742 (in 61
1 pairings) input.

## 33790 paired-reads (in 134 unique pairings) successfully merged out of 35910 (in 22
9 pairings) input.

## 36317 paired-reads (in 359 unique pairings) successfully merged out of 37314 (in 59
1 pairings) input.

## 41818 paired-reads (in 617 unique pairings) successfully merged out of 43002 (in 11
66 pairings) input.

## 29529 paired-reads (in 390 unique pairings) successfully merged out of 30030 (in 60
4 pairings) input.

## 40274 paired-reads (in 459 unique pairings) successfully merged out of 40492 (in 57
8 pairings) input.

## 36211 paired-reads (in 544 unique pairings) successfully merged out of 36481 (in 68
1 pairings) input.

## 39599 paired-reads (in 234 unique pairings) successfully merged out of 39651 (in 26
1 pairings) input.

## 32201 paired-reads (in 548 unique pairings) successfully merged out of 32921 (in 85
1 pairings) input.

## 32675 paired-reads (in 514 unique pairings) successfully merged out of 33062 (in 68
4 pairings) input.

## 25906 paired-reads (in 346 unique pairings) successfully merged out of 26421 (in 49
5 pairings) input.

## 32574 paired-reads (in 362 unique pairings) successfully merged out of 33173 (in 54
9 pairings) input.

save(file = "MergedReads2.RData", mergers)

load("MergedReads2.RData")

seqtab <- makeSequenceTable(mergers)
```

```
dim(seqtab)

## [1]   242 25096

seqtab.nochim <- removeBimeraDenovo(seqtab, verbose=TRUE, multithread = 12)

## Identified 23178 bimeras out of 25096 input sequences.

dim(seqtab.nochim)

## [1]  242 1918

save(file = "SeqTab2.RData", seqtab)

#library("openxlsx")

#write.xlsx(seqtab.nochim, file = "seqtab.nochim.xlsx")

getN <- function(x) sum(getUniques(x))

track <- cbind(out, sapply(dadaFs, getN), sapply(dadaRs, getN), sapply(mergers, getN),
rowSums(seqtab.nochim))

# If processing a single sample, remove the sapply calls: e.g. replace sapply(dadaFs,
getN) with getN(dadaFs)

colnames(track) <- c("input", "filtered", "denoisedF", "denoisedR", "merged", "nonchim
")

rownames(track) <- sample.names

head(track)

##         input filtered denoisedF denoisedR merged nonchim

## 13V3V4  47546    39830     39552     39712  39286   34183

## 14V3V4  57834    48601     48455     48526  48317   46645

## 238V3V4 57671    38897     38102     38693  36732   32141

## 239V3V4 30529    19134     18919     19074  18550   18017

## 240V3V4 63960    41533     41176     41389  40551   35466

## 241V3V4 51849    26986     26592     26876  26193   22204

sum(track[,6])/sum(track[,1]) #fraction of reads kept at the end

## [1] 0.5290161

hist(track[,6]/track[,1],20, xlab="Fraction Retained", main = NULL) #histogram of frac
tion of reads kept in each sample
```

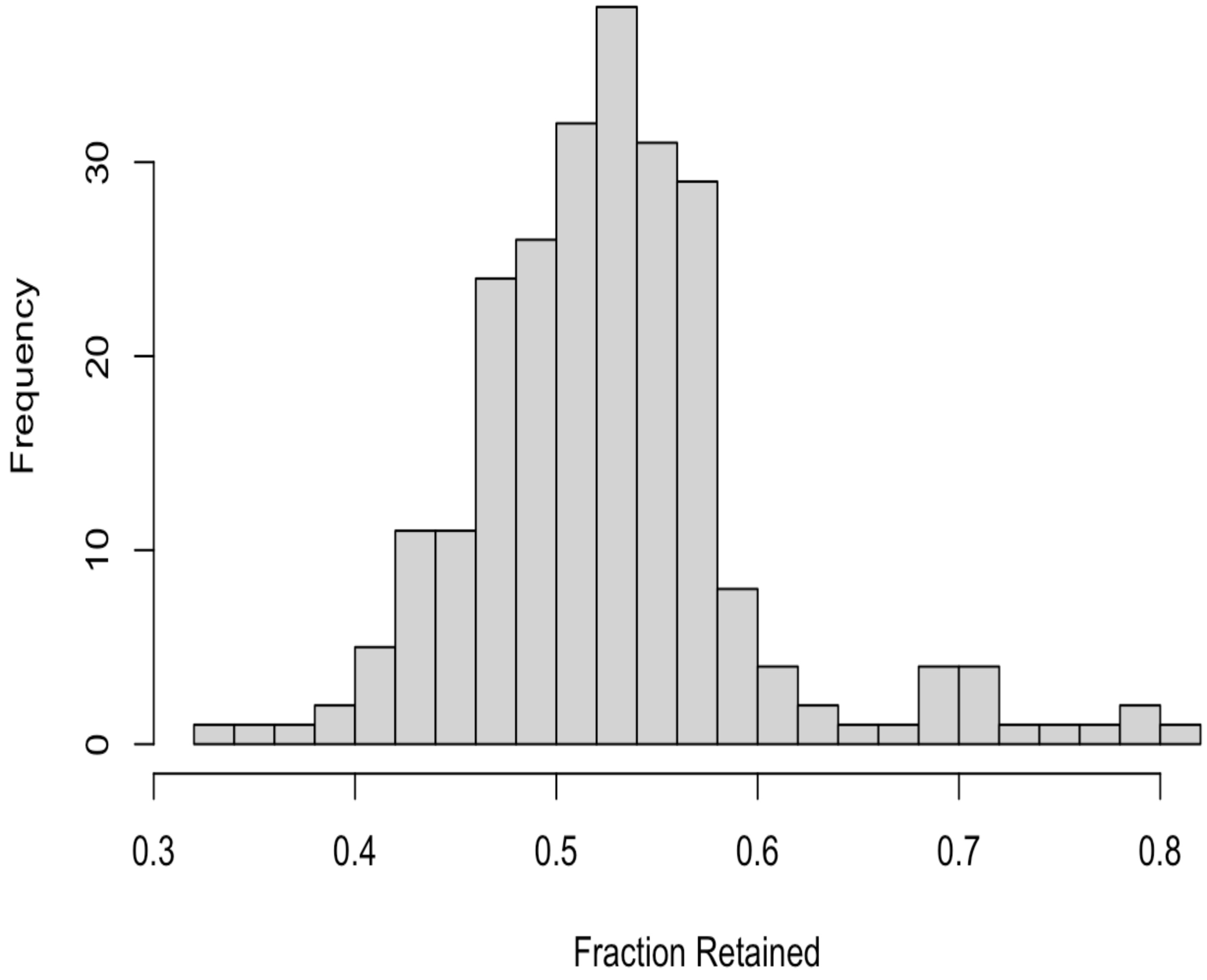


```
dim(seqtab.nochim)

## [1]  242 1918

hist(colSums(seqtab.nochim),100, xlab="Read Counts per SV", main=NULL) #histogram of r
ead counts per SV
```

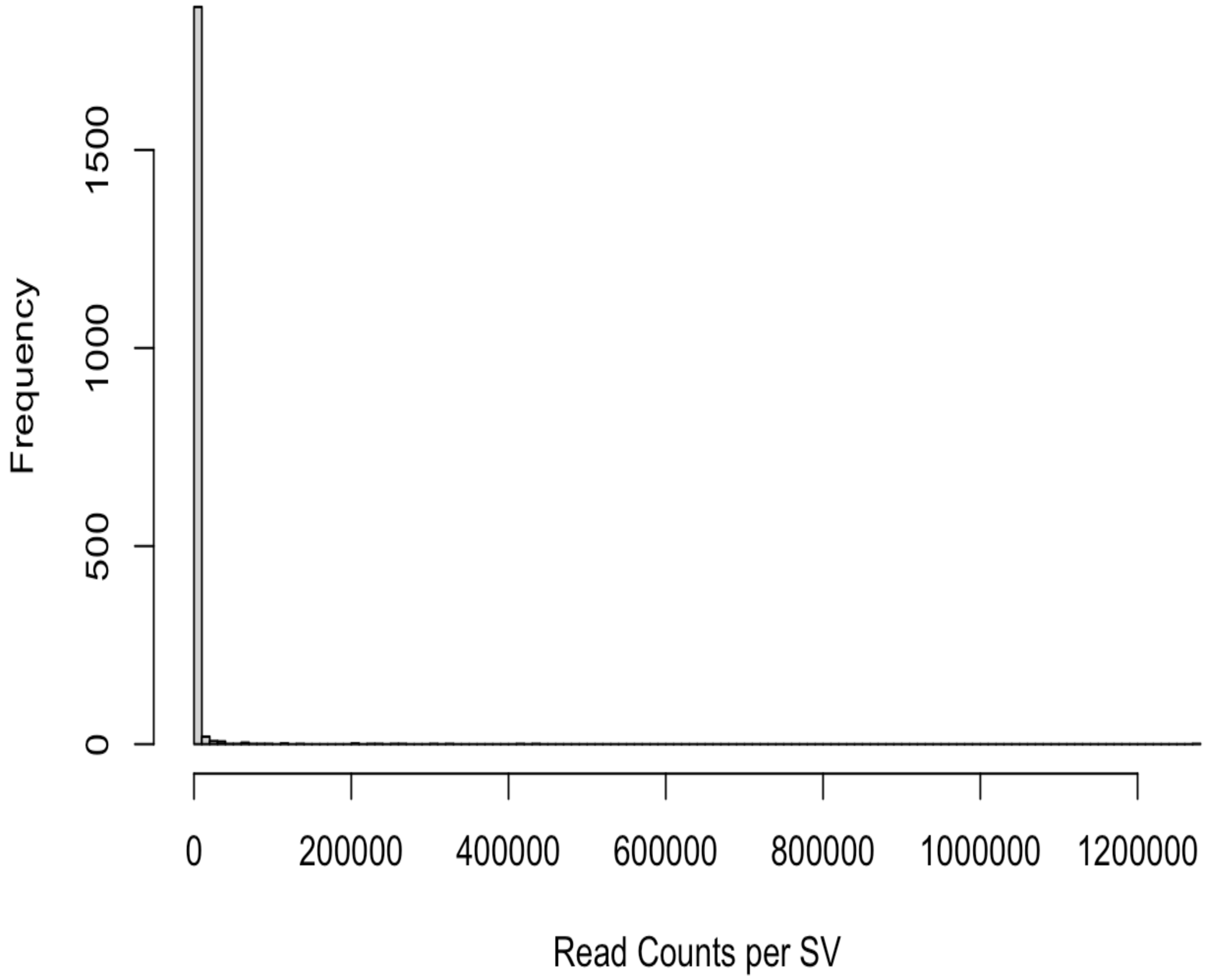


```
###make a fasta file for SPINGO
sink("FMT.fasta", append = T)
for(i in seq_len(ncol(seqtab.nochim))){
  cat(paste(">ASV",i, "\n", sep=""))
  cat(paste(colnames(seqtab.nochim))[i], "\n")
}
sink()
```

Filtering

Giovana

12/11/2021

```
#Running sequence in blast
###To get the sequence of an ambiguous ASV do this:
#getSequenceByASV("ASV262")
#Merge samples information with the SPINGO output
#load("SeqTab2.RData")
#library("openxlsx")
#ids <- read.xlsx("SPINGO.xlsx")

#get rid of V3, V4 which are the L1 (group) assignments from SPINGO
#ids[,c(3:4)] <- NULL
#names(ids) <- c("ASV", "Kmer_Similarity", "Genus", "Genus_Bootstrap", "Species", "Spe
cies_Bootstrap")

#counttab <- seqtab.nochim
#colnames(counttab) <- paste("ASV",1:ncol(seqtab.nochim),sep="") #rename column of seq
tab to ASV1, ASV2, ... , ASV3358

#counttab <- t(counttab)
#counttab <- as.data.frame(counttab)
#counttab$Species <- ids[match(rownames(counttab),ids$ASV),"Species"]

#counttab.agg <- aggregate(counttab[,-1*ncol(counttab)], by=counttab[,ncol(counttab),d
rop=F], sum)
#counttab.agg <- counttab.agg[order(rowSums(counttab.agg[,-1]), decreasing = T),]

#metadata <- read.xlsx("Metadata.xlsx")

#analysisData <- as.data.frame(t(counttab.agg[,-1]))
#colnames(analysisData) <- counttab.agg[,1]
#analysisData$ZymoSample <- colnames(counttab.agg[,-1])

#library("openxlsx")
```

```
#write.xlsx(analysisData, file = "analysisData.xlsx", rowNames=T)

#' Fitering method based on information loss and graph network


##Load packages

packages = c("readxl","Tmisc","dirmult","imputeTS","kableExtra","compositions","string
r","dplyr",
             "pracma","splus2R","matrixcalc","plotly","reshape","viridis","Hmisc","gri
dExtra","DT",
             "infotheo","networkD3","igraph","WGCNA","micEcon","decontam","inflection"
,"gplots","boot",
             "kableExtra","qwraps2","caret","ggrepel","network","ggnet","openxlsx")

invisible(lapply(packages, library,character.only=TRUE))

## Warning: package 'Tmisc' was built under R version 4.0.2

## Warning: package 'imputeTS' was built under R version 4.0.2

## Registered S3 method overwritten by 'quantmod':

##   method            from

##   as.zoo.data.frame zoo

## Warning: package 'kableExtra' was built under R version 4.0.2

## Warning: package 'compositions' was built under R version 4.0.2

## Welcome to compositions, a package for compositional data analysis.

## Find an intro with "? compositions"

##

## Attaching package: 'compositions'

## The following object is masked from 'package:Tmisc':

##

##     ellipses

## The following objects are masked from 'package:stats':

##

##     cor, cov, dist, var

## The following objects are masked from 'package:base':

##

##     %*%, norm, scale, scale.default

## Warning: package 'dplyr' was built under R version 4.0.2

##

## Attaching package: 'dplyr'

## The following object is masked from 'package:kableExtra':

##

##     group_rows
```

```
## The following objects are masked from 'package:stats':
##
##     filter, lag
## The following objects are masked from 'package:base':
##
##     intersect, setdiff, setequal, union
##
## Attaching package: 'pracma'
## The following object is masked from 'package:Tmisc':
##
##     Mode
##
## Attaching package: 'splus2R'
## The following object is masked from 'package:pracma':
##
##     peaks
## Warning: package 'plotly' was built under R version 4.0.2
## Loading required package: ggplot2
## Warning: package 'ggplot2' was built under R version 4.0.2
##
## Attaching package: 'plotly'
## The following object is masked from 'package:ggplot2':
##
##     last_plot
## The following object is masked from 'package:stats':
##
##     filter
## The following object is masked from 'package:graphics':
##
##     layout
##
## Attaching package: 'reshape'
## The following object is masked from 'package:plotly':
##
##     rename
## The following object is masked from 'package:dplyr':
##
```

```
##     rename
## Warning: package 'viridis' was built under R version 4.0.2
## Loading required package: viridisLite
## Warning: package 'viridisLite' was built under R version 4.0.2
## Warning: package 'Hmisc' was built under R version 4.0.2
## Loading required package: lattice
## Loading required package: survival
## Warning: package 'survival' was built under R version 4.0.2
## Loading required package: Formula
## Warning: package 'Formula' was built under R version 4.0.2
##
## Attaching package: 'Hmisc'
## The following object is masked from 'package:plotly':
##
##     subplot
## The following object is masked from 'package:pracma':
##
##     ceil
## The following objects are masked from 'package:dplyr':
##
##     src, summarize
## The following object is masked from 'package:Tmisc':
##
##     %nin%
## The following objects are masked from 'package:base':
##
##     format.pval, units
##
## Attaching package: 'gridExtra'
## The following object is masked from 'package:dplyr':
##
##     combine
##
## Attaching package: 'networkD3'
## The following object is masked from 'package:DT':
##
##     JS
```

```
## Warning: package 'igraph' was built under R version 4.0.2
##
## Attaching package: 'igraph'
## The following object is masked from 'package:plotly':
##
##     groups
## The following object is masked from 'package:matrixcalc':
##
##     %s%
## The following objects are masked from 'package:dplyr':
##
##     as_data_frame, groups, union
## The following object is masked from 'package:compositions':
##
##     normalize
## The following objects are masked from 'package:stats':
##
##     decompose, spectrum
## The following object is masked from 'package:base':
##
##     union
## Loading required package: dynamicTreeCut
## Loading required package: fastcluster
##
## Attaching package: 'fastcluster'
## The following object is masked from 'package:stats':
##
##     hclust
##
##
## Attaching package: 'WGCNA'
## The following object is masked from 'package:compositions':
##
##     cor
## The following object is masked from 'package:stats':
##
##     cor
```

```
##
## If you have questions, suggestions, or comments regarding one of the 'micEcon' pack
ages, please use a forum or 'tracker' at micEcon's R-Forge site:
## https://r-forge.r-project.org/projects/micecon/
## Warning: package 'gplots' was built under R version 4.0.2
##
## Attaching package: 'gplots'
## The following object is masked from 'package:stats':
##
##     lowess
##
## Attaching package: 'boot'
## The following object is masked from 'package:survival':
##
##     aml
## The following object is masked from 'package:lattice':
##
##     melanoma
## The following object is masked from 'package:pracma':
##
##     logit
## Warning: package 'qwraps2' was built under R version 4.0.2
##
## Attaching package: 'qwraps2'
## The following object is masked from 'package:boot':
##
##     logit
## The following object is masked from 'package:pracma':
##
##     logit
##
## Attaching package: 'caret'
## The following object is masked from 'package:survival':
##
##     cluster
## The following object is masked from 'package:compositions':
##
```

```
##     R2
## Warning: package 'ggrepel' was built under R version 4.0.2
## Warning: package 'network' was built under R version 4.0.2
## network: Classes for Relational Data
## Version 1.16.1 created on 2020-10-06.
## copyright (c) 2005, Carter T. Butts, University of California-Irvine
##                     Mark S. Handcock, University of California -- Los Angeles
##                     David R. Hunter, Penn State University
##                     Martina Morris, University of Washington
##                     Skye Bender-deMoll, University of Washington
##  For citation information, type citation("network").
##  Type help("network-package") to get started.
##
## Attaching package: 'network'
## The following objects are masked from 'package:igraph':
##
##     %c%, %s%, add.edges, add.vertices, delete.edges, delete.vertices,
##     get.edge.attribute, get.edges, get.vertex.attribute, is.bipartite,
##     is.directed, list.edge.attributes, list.vertex.attributes,
##     set.edge.attribute, set.vertex.attribute
## The following object is masked from 'package:Hmisc':
##
##     is.discrete
## The following object is masked from 'package:matrixcalc':
##
##     %s%
## Warning: package 'openxlsx' was built under R version 4.0.2
dta <- read.xlsx("analysisData.xlsx", rowNames = TRUE)
rownames(dta) = dta$ZymoSample
dta$...1=NULL
Samples =dta$ZymoSample
dta$ZymoSample=NULL



## functions
source("Info.R")
```

```
source("perm.info.R")

# number of bins
nbins.scott=function (x)
{
  h <- stats::sd(x)
  if (h == 0)
    h <- stats::mad(x, constant = 2)
  if (h > 0)
    ceiling(diff(range(x))/(3.49 * h * length(x)^(-1/3)))
  else 1L
}



## Fit the power law distribution to empirical data in log space
scaleFreeR2= function (connectivity,plt=TRUE,histo=TRUE){
  ## Use FD method for binning
  nbins = nbins.scott(connectivity)
  if(nbins == 1)
    nbins<-nbins+1
  k = connectivity
  discretized.k = cut(k, nbins)
  dk = tapply(k, discretized.k, mean)  ## mean of values in each interval
  p.dk = as.vector(tapply(k, discretized.k, length)/length(k)) ## relative freq
  breaks1 = seq(from = min(k), to = max(k), length = nbins +1)
  if(histo==TRUE){
    hist1 =hist(k, breaks = breaks1)
  }
  else{
    hist1 =hist(k, breaks = breaks1,plot = FALSE)
  }

  dk2 = hist1$mids
  dk = ifelse(is.na(dk), dk2, dk)
  dk = ifelse(dk == 0, dk2, dk)
  p.dk = ifelse(is.na(p.dk), 0, p.dk)
  log.dk = as.vector(log10(dk))
```

```
  log.p.dk = as.numeric(log10(p.dk + 1e-09))
  lm1 = lm(log.p.dk ~ log.dk)
  OUTPUT = data.frame(scaleFreeRsquared = round(summary(lm1)$r.squared,
                                                    2), slope = round(lm1$coefficients[[2]
], 2))
  title = paste(" scale free R^2=", as.character(round(summary(lm1)$r.squared,
                                                           2)), ", slope=", round(lm1$coef
ficients[[2]], 2))
  if(plt==TRUE){
    suppressWarnings(plot(log.dk, log.p.dk, xlab = "log10(k)",
                          ylab = "log10(p(k))", main = title))
    lines(log.dk, predict(lm1), col = 1)
  }

  OUTPUT
}



boot.R2= function(deg,b=NULL,plt=TRUE,histo=TRUE){
  nbins = nbins.scott(deg)
  if(nbins == 1)
    nbins<-nbins+1
  k = deg
  discretized.k = cut(k, nbins)
  dk = tapply(k, discretized.k, mean)  ## mean of values in each interval
  p.dk = as.vector(tapply(k, discretized.k, length)/length(k)) ## relative freq
  breaks1 = seq(from = min(k), to = max(k), length = nbins +1)
  if(histo==TRUE){
    hist1 =hist(k, breaks = breaks1)
  }
  else{
    hist1 =hist(k, breaks = breaks1,plot = FALSE)
  }

  dk2 = hist1$mids
  dk = ifelse(is.na(dk), dk2, dk)
```

```
  dk = ifelse(dk == 0, dk2, dk)
  p.dk = ifelse(is.na(p.dk), 0, p.dk)
  log.dk = as.vector(log10(dk))
  log.p.dk = as.numeric(log10(p.dk + 1e-09))
  dta = data.frame(log.p.dk,log.dk)
  rsq <- function(formula, data, indices){
    d<- data[indices,]
    fit<- lm(formula,data = d)
    return(summary(fit)$r.square)
  }
  results <- suppressWarnings(boot(data=dta, statistic=rsq,
                                   R=b, formula=log.p.dk~log.dk))
  rsqrd = mean(as.vector(results$t),na.rm=TRUE)
  return(rsqrd)
}
#Mutual Information
## We estimate mutual information and marginal entropies
##and normalized mutual information by joint entropy
mi.res = mutinformation(dta,method = "emp")   ## mutual information between each pair
of taxa
marg.res = apply(dta,2,entropy,method = "emp")  ## marginal entropy of each taxon

# calculate joint and conditional entropy
cond.ent= NULL
joint.ent= NULL
for ( i in 1: dim(dta)[2]){
  cond.ent.vec=c()
  joint.ent.vec=c()
  for(j in 1: dim(dta)[2]){
    cond.ent.vec = c(cond.ent.vec,round(condentropy(dta[,i],dta[,j],method="emp"),3))
    joint.ent.vec = c(joint.ent.vec,round(cond.ent.vec[j]+entropy(dta[,j],method = "em
p"),3))
  }
  cond.ent = rbind(cond.ent,cond.ent.vec)
  joint.ent = rbind(joint.ent,joint.ent.vec)
}
```

```
colnames(cond.ent) <-colnames(dta)
colnames(joint.ent) <-colnames(dta)

## weighted network adjacency matrix
I_adj_univ = round(mi.res/joint.ent,3) ## 2 decimal places makes the matrix to be symm
etric
#isSymmetric(I_adj_univ)

#  matrix symmetric
I_adj_univ[lower.tri(I_adj_univ)] = t(I_adj_univ)[lower.tri(I_adj_univ)]
#isSymmetric(I_adj_univ)
##calculate mean degree and R2(goodness of fit)

tau =seq(0.05,0.95,0.05)

# threshold based on median degree
R2=c()
mean.deg=c()
slope=c()
Rsqrd.boot=c()

for( i in 1: length(tau)){
  I_unw = ifelse(I_adj_univ<tau[i],0,1)
  diag(I_unw)=0
  bb = mean(apply(I_unw,1,sum))
  r2 = scaleFreeR2(apply(I_unw,1,sum),plt=FALSE,histo = FALSE)$scaleFreeRsquared
  gamma = scaleFreeR2(apply(I_unw,1,sum),plt=FALSE,histo=FALSE)$slope
  R2 = c(R2,r2)

  r2.boot = boot.R2(apply(I_unw,1,sum),b=5000,plt=FALSE,histo = FALSE)
  Rsqrd.boot = c(Rsqrd.boot,r2.boot)
  slope = c(slope,gamma)
  mean.deg=c(mean.deg,bb)
  #print(i)
}

df <- data.frame(thresh=tau,tau=tau,Rsqrd=Rsqrd.boot,meandeg = mean.deg,Slope=slope)
```

```
p1 = ggplot(data=df,aes(x=tau,y=(Rsqrd))) +
  geom_point(size=4,col="red") +
  # geom_line(size=1,col="red") +
  geom_text(aes(label="",hjust=0,vjust=2))+
  theme_bw()+xlab("Threshold")+ylab("R-squared")+
  theme(axis.text.x=element_text(angle=45, hjust=1,size=12)) +
  theme(axis.text.y=element_text(hjust=1,size=12),
        axis.title=element_text(size=12,face="bold"),
        legend.text=element_text(size=10),
        legend.title=element_text(size=12,face="bold")) +
  theme(plot.title = element_text(size=14))+
  xlab("Thresholds")+
  scale_x_continuous(breaks = seq(0.05,0.95,0.05))+
  #geom_text(aes(0.5, .92, label="R2:0.97", color=""))+
  theme(legend.position = "none") +
  scale_y_continuous(limits = c(0,1))+geom_line()

p2=ggplot(data=df,aes(x=tau,y=(meandeg))) +
  geom_point(size=4,col="red") +
  #geom_line(size=1,col="red") +
  geom_text(aes(label="",hjust=0,vjust=2))+
  theme_bw()+xlab("Threshold")+ylab("mean degree")+
  theme(axis.text.x=element_text(angle=45, hjust=1,size=12)) +
  theme(axis.text.y=element_text(hjust=1,size=12),
        axis.title=element_text(size=12,face="bold"),
        legend.text=element_text(size=10),
        legend.title=element_text(size=12,face="bold")) +
  theme(plot.title = element_text(size=14))+
  xlab("Thresholds")+
  scale_x_continuous(breaks = seq(0.05,0.95,0.05))+
  theme(legend.position = "none")+geom_line()

grid.arrange(p1,p2,ncol=2)
```

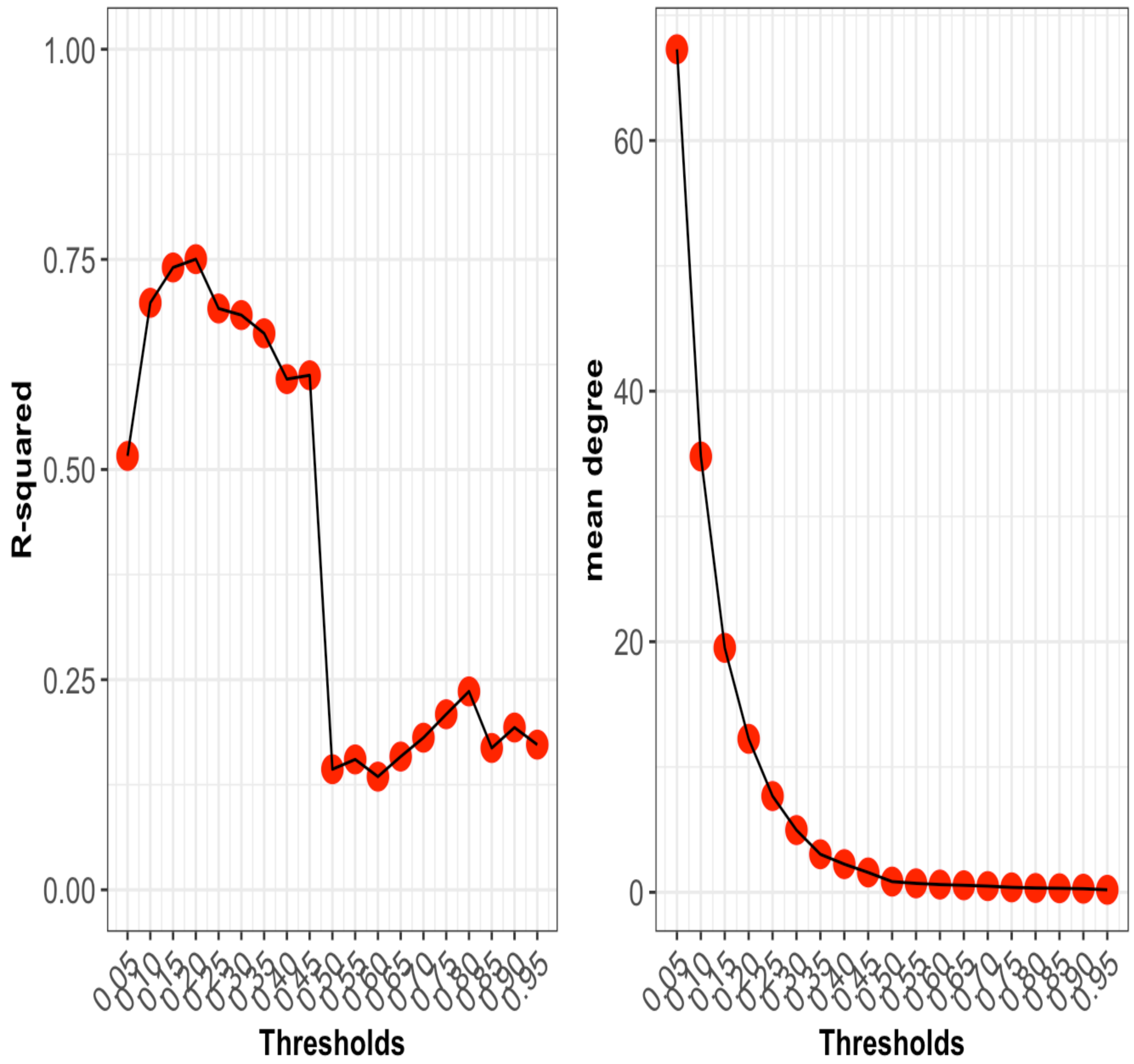


```
##Choose tau that maximizes goodness of fit and binarize the information adjacency mat
rix


tau1=tau[which(R2==max(R2))]

I_unw = ifelse(I_adj_univ<tau1,0,1)

diag(I_unw)=0

R2

##  [1] 0.53 0.81 0.82 0.85 0.80 0.79 0.77 0.71 0.75 0.09 0.11 0.07 0.12 0.15 0.17

## [16] 0.19 0.12 0.15 0.06
```

```
which.max(R2)
## [1] 4
tau[4]
## [1] 0.2
net = network(I_unw, directed = FALSE)
#network.vertex.names(net)

ggnet2(net,  palette = "Set2",size=1,  label = TRUE,
       label.size = 3,mode = "kamadakawai",legend.size = 0)
## Loading required package: sna
## Warning: package 'sna' was built under R version 4.0.2
## Loading required package: statnet.common
## Warning: package 'statnet.common' was built under R version 4.0.2
##
## Attaching package: 'statnet.common'
## The following object is masked from 'package:compositions':
##
##     ult
## The following object is masked from 'package:base':
##
##     order
## sna: Tools for Social Network Analysis
## Version 2.6 created on 2020-10-5.
## copyright (c) 2005, Carter T. Butts, University of California-Irvine
##  For citation information, type citation("sna").
##  Type help(package="sna") to get started.
##
## Attaching package: 'sna'
## The following objects are masked from 'package:igraph':
##
##     betweenness, bonpow, closeness, components, degree, dyad.census,
##     evcent, hierarchy, is.connected, neighborhood, triad.census
## The following object is masked from 'package:pracma':
##
##     cutpoints
## Loading required package: scales
##
```

```
## Attaching package: 'scales'
## The following object is masked from 'package:viridis':
##
##     viridis_pal
## Warning: `guides(<scale> = FALSE)` is deprecated. Please use `guides(<scale> =
## "none")` instead.
## Warning: `guides(<scale> = FALSE)` is deprecated. Please use `guides(<scale> =
## "none")` instead.

## Warning: `guides(<scale> = FALSE)` is deprecated. Please use `guides(<scale> =
## "none")` instead.
```

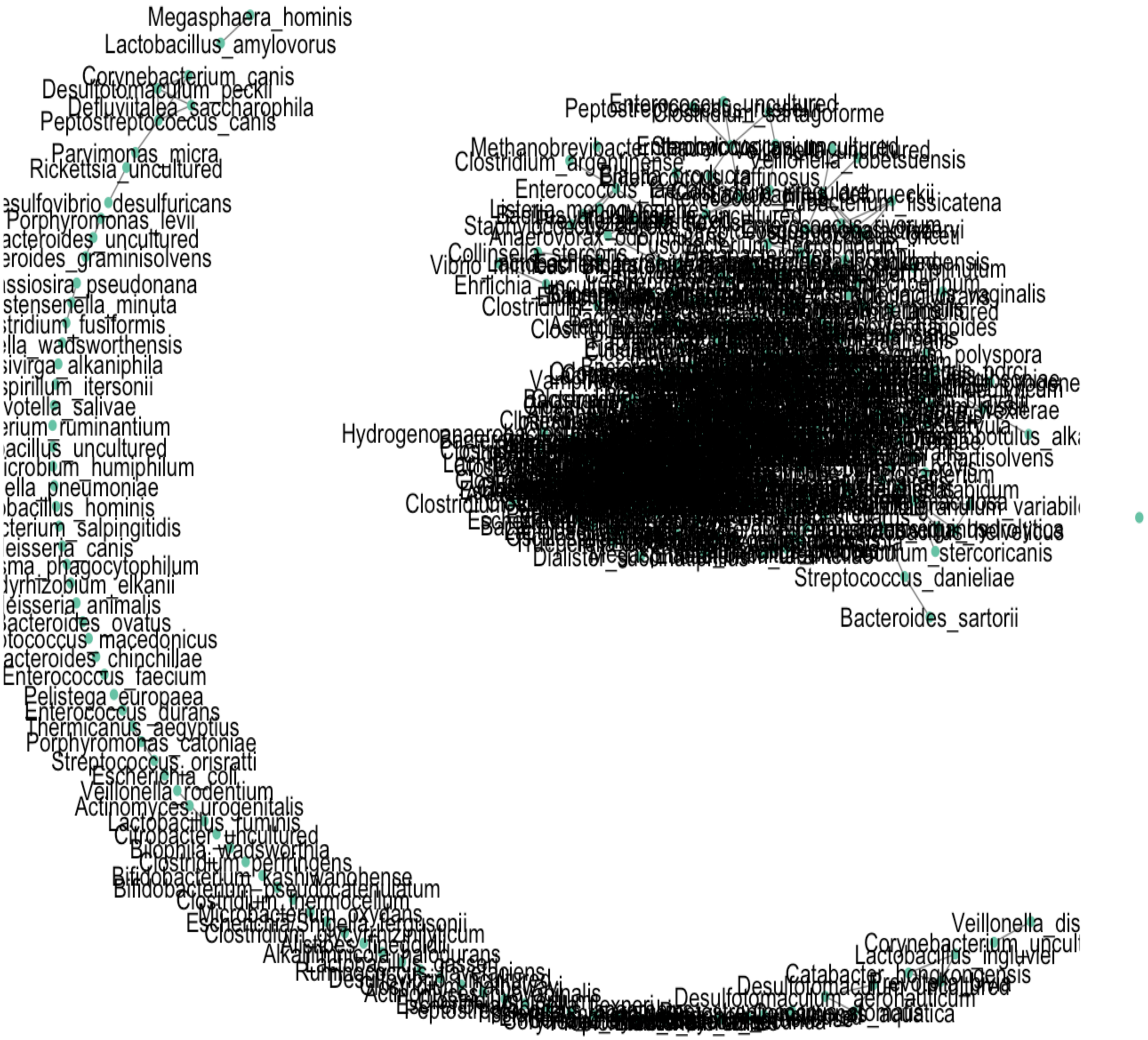


```
##Choose ASVs using both unweighted and weighted network based on their 3 rd qurtile o
f degree


thresh_unw= summary(apply(I_unw,1,sum))[5]   ## based on 3rd quantile

deggrt1 = apply(I_unw,1,sum)[which(as.vector(apply(I_unw,1,sum))>thresh_unw)]


thresh_wnw= as.numeric(summary(apply(I_adj_univ,1,sum))[5])

deggrt2 = apply(I_adj_univ,1,sum)[which(as.vector(apply(I_adj_univ,1,sum))>thresh_wnw)
]
```

```
## unweighted network
output= data.frame(degree=as.vector(deggrt1)[order(as.vector(deggrt1))],
                   Taxonomy=names(deggrt1[order(as.vector(deggrt1))]))  ## 99 ASVs

## weighted network
output2= data.frame(degree=as.vector(deggrt2)[order(as.vector(deggrt2))],
                    Taxonomy=names(deggrt2[order(as.vector(deggrt2))])) ## 103 ASVs
##Information Loss calculation based on weighted network

diag(I_adj_univ)=0
connect_w=sort(rowSums(I_adj_univ,na.rm=TRUE),decreasing=TRUE)
sort.connect = sort(connect_w)
vec= c(seq(0.01,1,0.05),0.99)



InfoLoss=c()
q2.data.ls=list()
for ( i in 1:length(vec)){
  #print(i)
  quantile(sort.connect, vec[i])
  q1.data=sort.connect[sort.connect > quantile(sort.connect, vec[i])]
  q2.data=names(sort.connect[sort.connect <= quantile(sort.connect, vec[i])])
  q2.data.ls[[i]]=q2.data
  names(q2.data.ls)[[i]]=vec[i]
  trunc.dta=dta[,names(q1.data)]

  if(is.vector(trunc.dta)==TRUE){
    trunc.mi = entropy(trunc.dta,method = "emp")} else{
      trunc.mi = mutinformation(trunc.dta,method = "emp")}

  info.filt =psych::tr(t(trunc.mi)%*%trunc.mi)

  ## norm of truncated MI for whole data
  results = mutinformation(dta, method="emp")
  info.all = psych::tr(t(results)%*%results)
```

```
  ## information loss
  InfoLos=round(1-(info.filt/info.all),2)

  InfoLoss=c(InfoLoss,InfoLos)
}

DInfloss = diff(InfoLoss)



p1=ggplot()+geom_line(aes(x=vec,y=InfoLoss),color="coral",size=1)+
  geom_point(aes(x=vec,y=InfoLoss),color="coral",size= 3)+theme_bw()+
  scale_x_continuous(breaks = vec,limits=c(0, 1))+
  theme(axis.text.x = element_text(angle = 60, hjust = 1))+
  ylab("Information loss")+
  xlab("Cutoff")+
  theme_bw()+
  theme(axis.text.x=element_text(angle=45, hjust=1,size=12)) +
  theme(axis.text.y=element_text(hjust=1,size=12),
        axis.title=element_text(size=12,face="bold"),
        legend.text=element_text(size=10),
        legend.title=element_text(size=12,face="bold")) +
  theme(plot.title = element_text(size=14))+
  xlab("Thresholds")+
  theme(legend.position = "none")

name=c()
for ( i in 1: length(vec)-1){name=c(name,paste0(vec[i+1],"-", vec[i]))}
name=name[-1]

p2=ggplot()+geom_line(aes(x=as.character(vec[-1]),y=DInfloss),group=1,color="coral",si
ze=1)+
  geom_point(aes(x=as.character(vec[-1]),y=DInfloss,group=1),color="coral",size=3)+the
me_bw()+
  scale_x_discrete(breaks=as.character(vec[-1]),
                   labels=name)+
```

```
  theme(axis.text.x=element_text(angle=45, hjust=1,size=12)) +
  theme(axis.text.y=element_text(hjust=1,size=12),
        axis.title=element_text(size=12,face="bold"),
        legend.text=element_text(size=10),
        legend.title=element_text(size=12,face="bold")) +
  theme(plot.title = element_text(size=14))+
  xlab("Thresholds")+
  theme(legend.position = "none") +
  ylab("Difference Information loss")+xlab("Diff. Threshold")

grid.arrange(p1,p2,ncol=2)
```

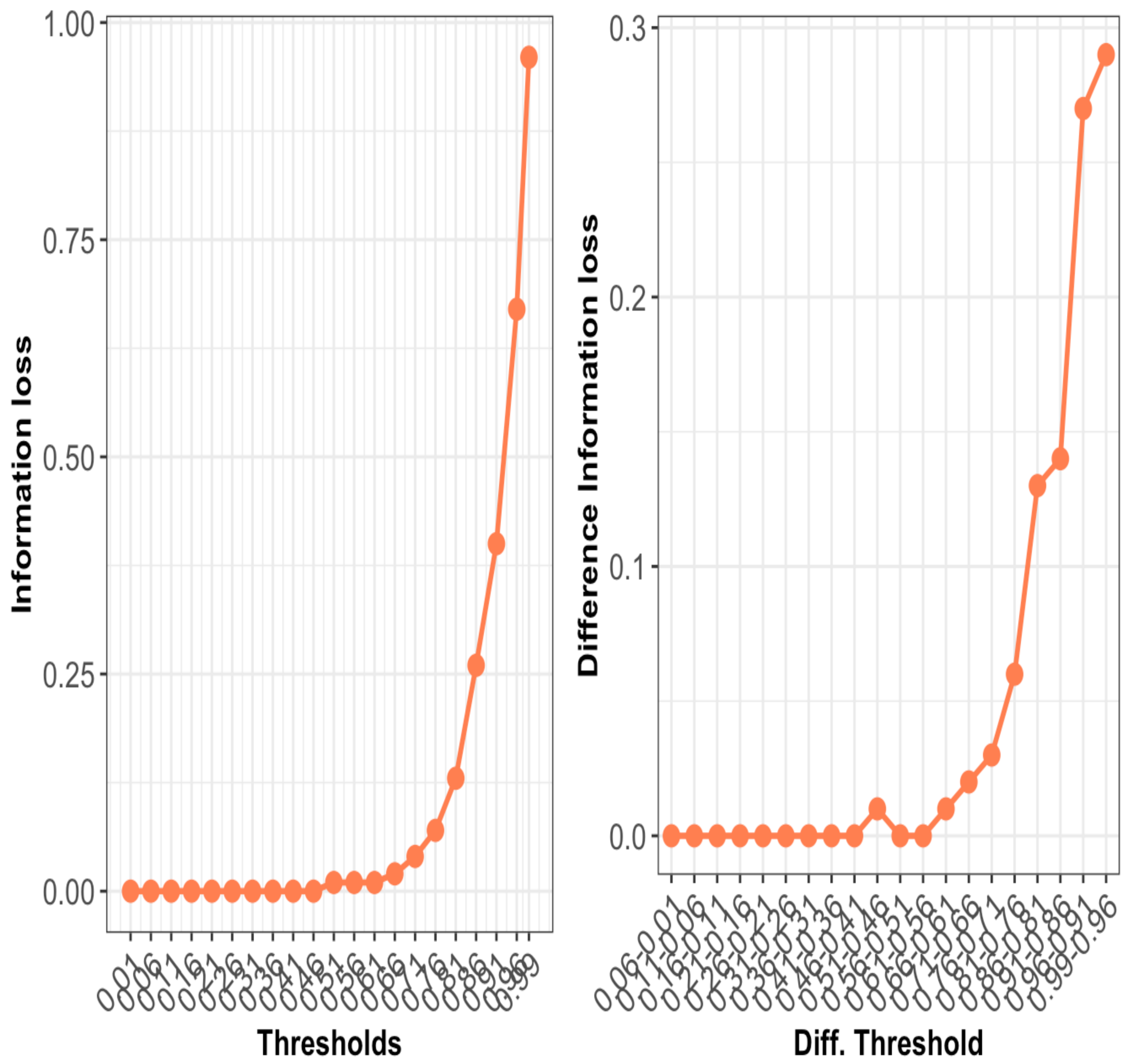


```
##Permutation Test
PS = dta
cutoff<- c(seq(0.01,1,0.05))
num = 100
y = InformationLoss(ps=PS,cutoff,sort.connect)
```

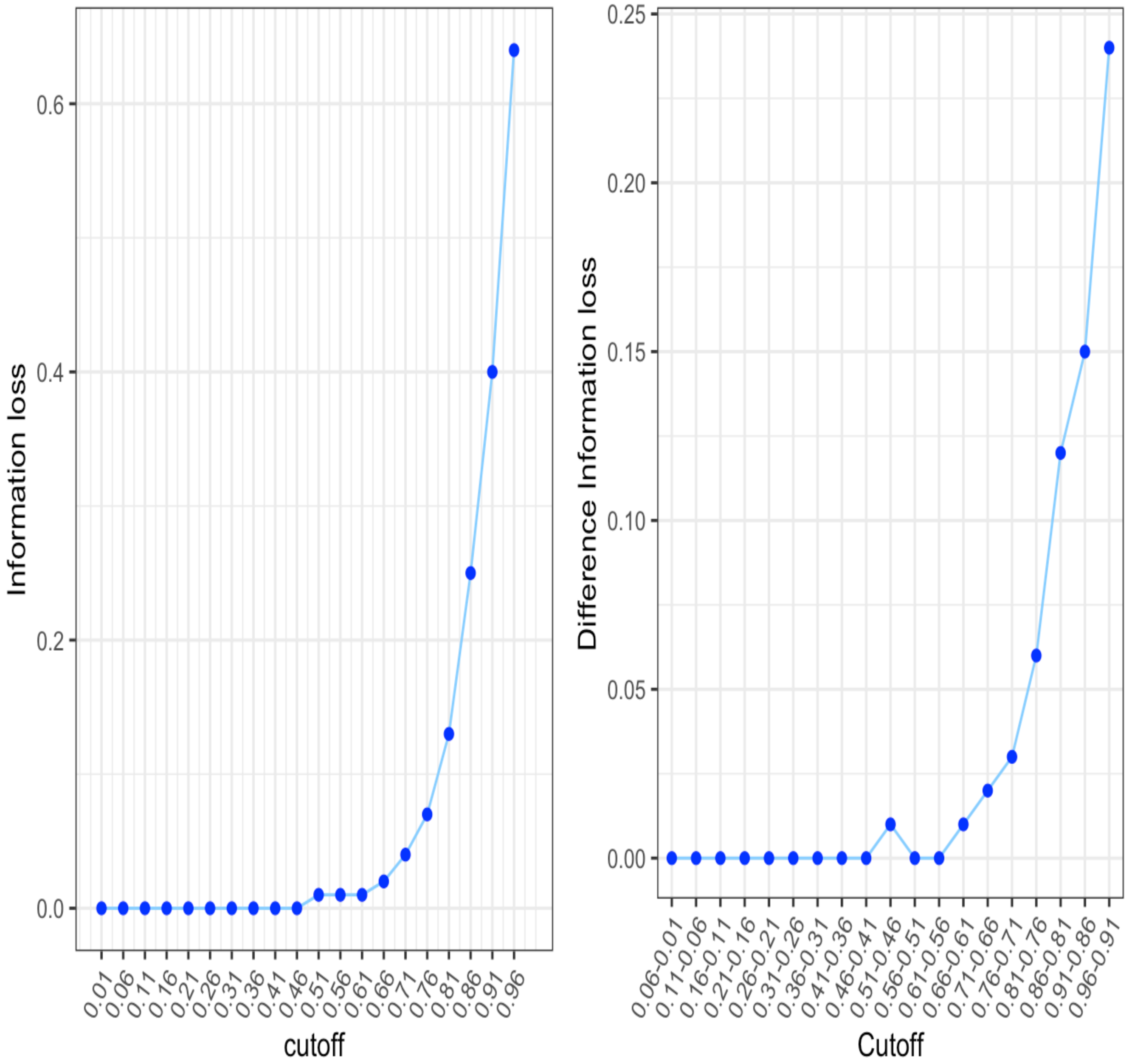


```
res=perm.mi(ps=PS,Num=y$num,M=num,y=y,cutoff,prbar = FALSE)
##
## Attaching package: 'svMisc'
## The following object is masked from 'package:caret':
##
##     progress
## The following object is masked from 'package:utils':
##
##     ?
```

```
Pval = res$p_vals

Pval

##            X.pvalsperm2.

## 0.06-0.01          1.00

## 0.11-0.06          1.00

## 0.16-0.11          1.00

## 0.21-0.16          1.00

## 0.26-0.21          1.00

## 0.31-0.26          1.00

## 0.36-0.31          1.00

## 0.41-0.36          1.00

## 0.46-0.41          1.00

## 0.51-0.46          0.95

## 0.56-0.51          1.00

## 0.61-0.56          1.00

## 0.66-0.61          0.93

## 0.71-0.66          0.63

## 0.76-0.71          0.40

## 0.81-0.76          0.04

## 0.86-0.81          0.00

## 0.91-0.86          0.00

## 0.96-0.91          0.00

bh = (c(1:length(Pval$X.pvalsperm2.)/length(Pval$X.pvalsperm2.)))*0.1


cbind(Pval$X.pvalsperm2.,bh,c(1:length(Pval$X.pvalsperm2.)))

##                        bh

##  [1,] 1.00 0.005263158  1

##  [2,] 1.00 0.010526316  2

##  [3,] 1.00 0.015789474  3

##  [4,] 1.00 0.021052632  4

##  [5,] 1.00 0.026315789  5

##  [6,] 1.00 0.031578947  6

##  [7,] 1.00 0.036842105  7

##  [8,] 1.00 0.042105263  8

##  [9,] 1.00 0.047368421  9

## [10,] 0.95 0.052631579 10

## [11,] 1.00 0.057894737 11
```

```
## [12,] 1.00 0.063157895 12
## [13,] 0.93 0.068421053 13
## [14,] 0.63 0.073684211 14
## [15,] 0.40 0.078947368 15
## [16,] 0.04 0.084210526 16
## [17,] 0.00 0.089473684 17
## [18,] 0.00 0.094736842 18
## [19,] 0.00 0.100000000 19
##Remove ASVs with degree less than 76 percentile 5% sig. level

outy=data.frame(colnames(dta)[-which(colnames(dta)%in%q2.data.ls$`0.76`)])
colnames(outy) = "Taxa to keep"
outy$num = c(1:dim(outy)[1])
outy = data.frame(outy$num,outy$`Taxa to keep`)
colnames(outy) = c("number","Taxa To Keep")
outy
##    number                         Taxa To Keep
## 1       1                Bifidobacterium_longum
## 2       2          Faecalibacterium_prausnitzii
## 3       3              Lactobacillus_uncultured
## 4       4                            Uncultured
## 5       5                  Ruminococcus_torques
## 6       6          Butyricicoccus_pullicaecorum
## 7       7                    Clostridium_nexile
## 8       8              Collinsella_aerofaciens
## 9       9              Lactobacillus_johnsonii
## 10     10                    Blautia_uncultured
## 11     11                   Ruminococcus_gnavus
## 12     12            Streptococcus_uncultured
## 13     13                  Bacteroides_fragilis
## 14     14                  Bacteroides_vulgatus
## 15     15                          Blautia_luti
## 16     16              Bifidobacterium_bifidum
## 17     17    Escherichia/Shigella_uncultured
## 18     18          Bifidobacterium_uncultured
## 19     19                 Gallibacterium_anatis
## 20     20                Clostridium_uncultured
```

```
## 21      21                    Veillonella_magna
## 22      22                   Veillonella_caviae
## 23      23                 Megasphaera_elsdenii
## 24      24                 Prevotella_stercorea
## 25      25                Dorea_formicigenerans
## 26      26                  Alloprevotella_rava
## 27      27                     Prevotella_copri
## 28      28                  Gemmiger_formicilis
## 29      29  Lachnoclostridium_urinimassiliense
## 30      30                     Blautia_schinkii
## 31      31                  Clostridium_ramosum
## 32      32               Flavonifractor_plautii
## 33      33            Streptococcus_pleomorphus
## 34      34              Akkermansia_muciniphila
## 35      35                  Ruminococcus_bromii
## 36      36     Pseudoflavonifractor_capillosus
## 37      37       Clostridium_lactatifermentans
## 38      38                 Enterococcus_cecorum
## 39      39 Phascolarctobacterium_succinatutens
## 40      40              Lactobacillus_salivarius
## 41      41                    Coprococcus_comes
## 42      42                   Ruminococcus_obeum
## 43      43              Sutterella_stercoricanis
## 44      44                   Sharpea_azabuensis
## 45      45            Bacteroides_xylanisolvens
## 46      46                     Prevotella_oris
## 47      47                Lactobacillus_reuteri
## 48      48        Barnesiella_intestinihominis
## 49      49                Clostridium_cadaveris
## 50      50         Clostridium_saccharolyticum
## 51      51                  Eubacterium_biforme
## 52      52            Butyrivibrio_fibrisolvens
## 53      53    Parasutterella_excrementihominis
## 54      54                 Clostridium_oroticum
## 55      55        Bacteroides_thetaiotaomicron
## 56      56                Bacteroides_uniformis
## 57      57        Bifidobacterium_pseudolongum
```

```
## 58      58       Eubacterium_coprostanoligenes
## 59      59               Howardella_ureilytica
## 60      60            Bacteroides_acidifaciens
## 61      61               Ruminococcus_lactaris
## 62      62          Parabacteroides_distasonis
## 63      63               Clostridium_hylemonae
## 64      64                    Prevotella_fusca
## 65      65           Parabacteroides_johnsonii
## 66      66                  Clostridium_leptum
## 67      67                  Blautia_glucerasea
## 68      68                 Eubacterium_limosum
## 69      69                 Anaerostipes_caccae
## 70      70         Intestinimonas_massiliensis
## 71      71         Anaerophaga_thermohalophila
## 72      72                   Eggerthella_lenta
## 73      73        Alkaliphilus_crotonatoxidans
## 74      74                  Oribacterium_sinus
## 75      75        Allisonella_histaminiformans
## 76      76               Clostridium_aldenense
## 77      77              Clostridium_spiroforme
## 78      78         Oscillibacter_valericigenes
## 79      79              Parabacteroides_merdae
## 80      80               Pedobacter_alluvionis
## 81      81    Intestinimonas_butyriciproducens
## 82      82                  Olsenella_umbonata
## 83      83                Clostridium_scindens
## 84      84          Clostridium_methylpentosum
## 85      85             Vallitalea_guaymasensis
## 86      86                 Prevotella_baroniae
## 87      87           Clostridium_asparagiforme
## 88      88            Mogibacterium_uncultured
## 89      89                Solobacterium_moorei
## 90      90                   Prevotella_buccae
##  Shared ASVs that were chosen to keep based on permutation test on weighted network
as well as based on unweighted network with power-law distribution fit
```

```
shrd = as.character(outy$`Taxa To Keep`)[which((as.character(outy$`Taxa To Keep`)%in%as.character(output$Taxonomy)))]

shrd= data.frame(shrd)

shrd$num = c(1:dim(shrd)[1])

shrd = data.frame(shrd$num,shrd$shrd)

colnames(shrd) = c("number","Taxa shared")


shrd

##    number                    Taxa shared
## 1       1           Bifidobacterium_longum
## 2       2      Faecalibacterium_prausnitzii
## 3       3         Lactobacillus_uncultured
## 4       4                       Uncultured
## 5       5             Ruminococcus_torques
## 6       6      Butyricicoccus_pullicaecorum
## 7       7               Clostridium_nexile
## 8       8          Collinsella_aerofaciens
## 9       9          Lactobacillus_johnsonii
## 10     10               Blautia_uncultured
## 11     11              Ruminococcus_gnavus
## 12     12         Streptococcus_uncultured
## 13     13             Bacteroides_fragilis
## 14     14             Bacteroides_vulgatus
## 15     15                     Blautia_luti
## 16     16          Bifidobacterium_bifidum
## 17     17   Escherichia/Shigella_uncultured
## 18     18       Bifidobacterium_uncultured
## 19     19            Gallibacterium_anatis
## 20     20           Clostridium_uncultured
## 21     21               Veillonella_magna
## 22     22              Veillonella_caviae
## 23     23             Megasphaera_elsdenii
## 24     24             Prevotella_stercorea
## 25     25           Dorea_formicigenerans
## 26     26              Alloprevotella_rava
## 27     27                 Prevotella_copri
## 28     28             Gemmiger_formicilis
```

```
## 29      29  Lachnoclostridium_urinimassiliense
## 30      30                    Blautia_schinkii
## 31      31                 Clostridium_ramosum
## 32      32              Flavonifractor_plautii
## 33      33           Streptococcus_pleomorphus
## 34      34             Akkermansia_muciniphila
## 35      35                 Ruminococcus_bromii
## 36      36     Pseudoflavonifractor_capillosus
## 37      37       Clostridium_lactatifermentans
## 38      38                Enterococcus_cecorum
## 39      39 Phascolarctobacterium_succinatutens
## 40      40            Lactobacillus_salivarius
## 41      41                  Coprococcus_comes
## 42      42                 Ruminococcus_obeum
## 43      43            Sutterella_stercoricanis
## 44      44                  Sharpea_azabuensis
## 45      45           Bacteroides_xylanisolvens
## 46      46                     Prevotella_oris
## 47      47              Lactobacillus_reuteri
## 48      48        Barnesiella_intestinihominis
## 49      49              Clostridium_cadaveris
## 50      50         Clostridium_saccharolyticum
## 51      51                 Eubacterium_biforme
## 52      52           Butyrivibrio_fibrisolvens
## 53      53    Parasutterella_excrementihominis
## 54      54                Clostridium_oroticum
## 55      55              Bacteroides_uniformis
## 56      56        Bifidobacterium_pseudolongum
## 57      57       Eubacterium_coprostanoligenes
## 58      58              Howardella_ureilytica
## 59      59              Ruminococcus_lactaris
## 60      60              Clostridium_hylemonae
## 61      61                   Prevotella_fusca
## 62      62           Parabacteroides_johnsonii
## 63      63                 Clostridium_leptum
## 64      64                 Blautia_glucerasea
## 65      65                Eubacterium_limosum
```

```
## 66     66                 Anaerostipes_caccae
## 67     67         Intestinimonas_massiliensis
## 68     68         Anaerophaga_thermohalophila
## 69     69                   Eggerthella_lenta
## 70     70        Alkaliphilus_crotonatoxidans
## 71     71                  Oribacterium_sinus
## 72     72         Oscillibacter_valericigenes
## 73     73             Parabacteroides_merdae
## 74     74              Pedobacter_alluvionis
## 75     75    Intestinimonas_butyriciproducens
## 76     76                  Olsenella_umbonata
## 77     77          Clostridium_methylpentosum
## 78     78             Vallitalea_guaymasensis
## 79     79                 Prevotella_baroniae
## 80     80                Solobacterium_moorei
## 81     81                   Prevotella_buccae
##Bootstrap Hypothesis Test
#cutoff<- c(seq(0.01,1,0.05))
#yboot = InformationLossboot(ps=PS,cutoff,total = sort.connect)

#DInflossabove = (yboot$DInfloss)
#total.cov = yboot$tot.covar
#under = sqrt(RcppRoll::roll_sum(total.cov,2))
#t = DInflossabove/under
#b=500

#res.boot = boot.hypo.test(data=PS,cutoff,DInflossabove,B = b,Teststat = t,total = sor
t.connect)

#name=c()
#for ( i in 1: length(cutoff)-1){name=c(name,paste0(cutoff[i+1],"-", cutoff[i]))}
#name = name[-1]

#load("~/Documents/Filtering Base on Mutual Information/data/bootstrapres.RData")

#cbind(name,res.boot)
```

```
## benjamin-hochberg method to FDR in multiple testing.
#Pval_ordboot= sort(res.boot)
#bh.boot = (c(1:length(Pval_ordboot)/length(Pval_ordboot)))*0.1

#cbind(Pval_ordboot,bh.boot,c(1:length(Pval_ordboot)))
```

Data Analysis

Giovana Slanzon

12/22/2021

```
## After filtering the data, we have 81 ASVs to keep out of 373 total ASVs
#Extract certain ASVs from ASV count table

#Saving 81 ASVs after filtering the data
saveRDS(shrd, file = "shrd.rds")
asvs <- readRDS("shrd.rds")

asvs = as.character(asvs$`Taxa shared`)

library("readxl")
data <- read_excel("analysisData.xlsx")

analysisData2 = data[,asvs]
rownames(output) = data$...1

library("openxlsx")
write.xlsx(analysisData2, file = "analysisData2.xlsx", row.names=T)
library("readxl")
#analysisData2 has the 3 FMT product samples; analysisData3 only has the fecal samples
without the FMT product samples
analysisData2 <- read_excel("analysisData2.xlsx")
analysisData3 <- read_excel("analysisData3.xlsx")
#Alpha-Diversity
library("vegan")
library("ggplot2")
```

```
library("lme4")
library("emmeans")
library("magrittr")
library("dplyr")
library("tidyr")
library("lmerTest")
library("car")
library("effects")
library("MASS")
library("ggpubr")

#Model for Alpha diversity Shannon
Shannon_Index <- diversity(analysisData3[,2:81], "shannon") #excluding metadata
hist(Shannon_Index,20)
```

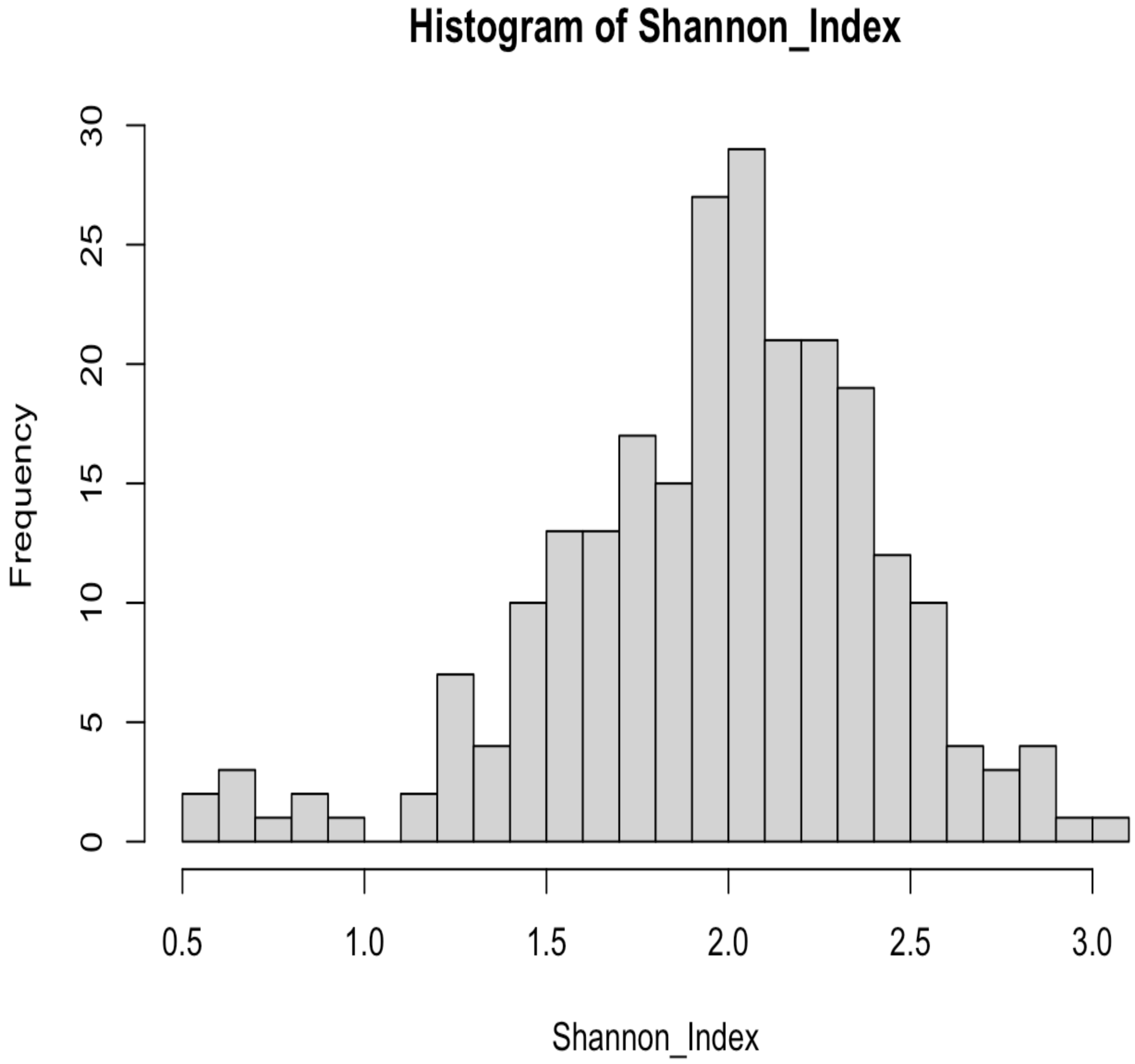


```
Shannon_Index <- as.data.frame(Shannon_Index)
shanonn <- lmer(Shannon_Index ~ SampleType + FMT + Breed + SubGroup + (1|Animal),
                       data = Shannon_Index)


emmip(shanonn, ~ SampleType | FMT, type = "response", CIs = T) + theme_bw() + ylab("Sh
annon Diversity") + scale_size_manual(values=c(0,0)) +
aes(size = "FMT") +
guides(col = F, size = F) +
theme(legend.position="bottom") #to plot
```

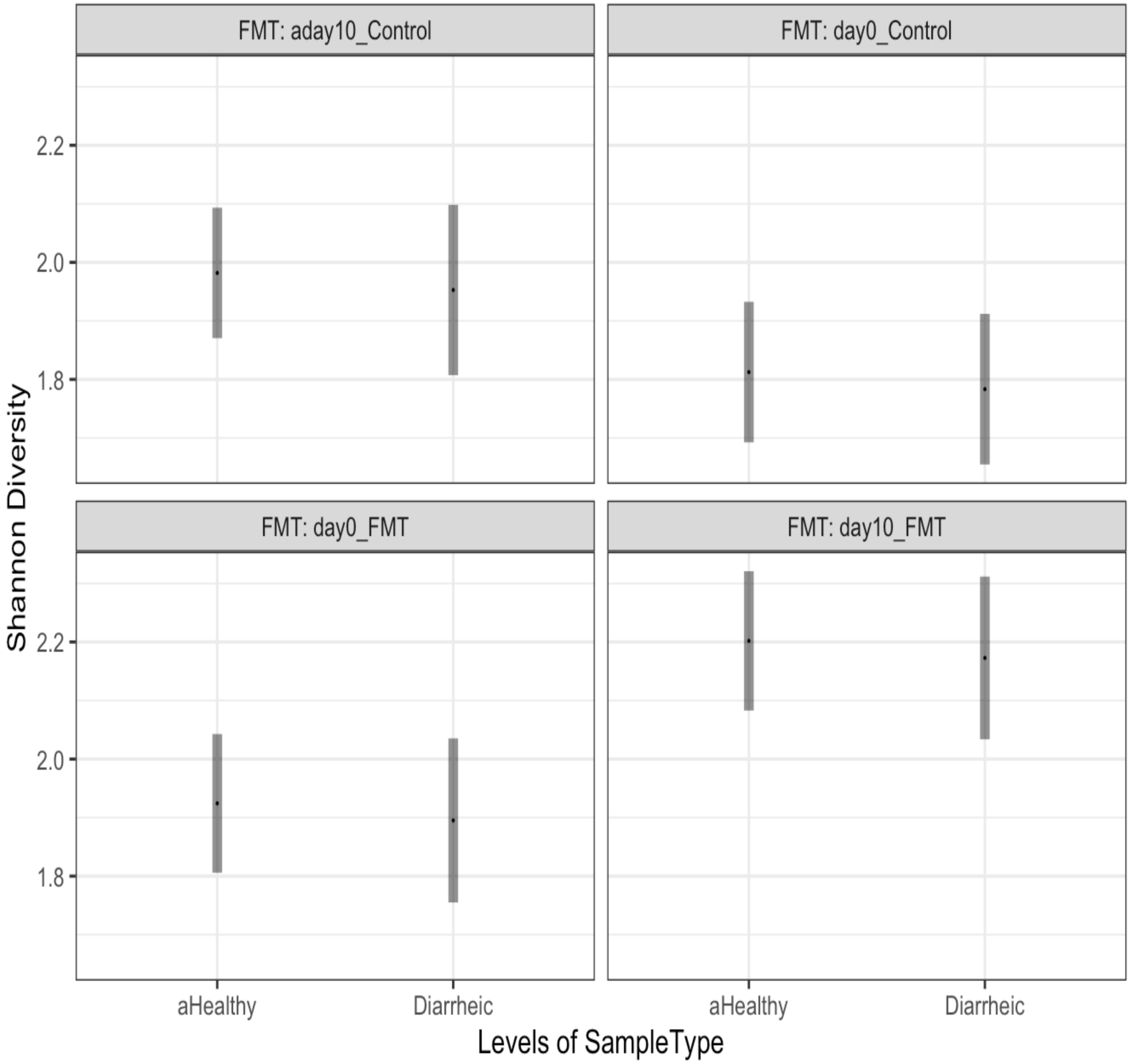


```
summary(shanonn)
## Linear mixed model fit by REML. t-tests use Satterthwaite's method [
## lmerModLmerTest]
## Formula: Shannon_Index ~ SampleType + FMT + Breed + SubGroup + (1 | Animal)
##    Data: Shannon_Index
##
## REML criterion at convergence: 298
##
## Scaled residuals:
```

```
##      Min       1Q   Median       3Q      Max
## -3.01725 -0.46970  0.07547  0.62745  2.28608
##
## Random effects:
##  Groups   Name        Variance Std.Dev.
##  Animal   (Intercept) 0.03871  0.1967
##  Residual             0.14595  0.3820
## Number of obs: 242, groups:  Animal, 121
##
## Fixed effects:
##                       Estimate Std. Error        df t value Pr(>|t|)
## (Intercept)            1.95510    0.08843 158.41735  22.109  < 2e-16 ***
## SampleTypeDiarrheic  -0.02916    0.05901 204.40348  -0.494  0.62179
## FMTday0_Control      -0.16939    0.07009 121.52347  -2.417  0.01714 *
## FMTday0_FMT          -0.05759    0.07870 223.10331  -0.732  0.46506
## FMTday10_FMT          0.22001    0.07880 223.21370   2.792  0.00569 **
## BreedJersey          -0.05256    0.06802 115.97444  -0.773  0.44121
## BreedJerseyCross     -0.03953    0.08682 116.09574  -0.455  0.64977
## SubGroup              0.01157    0.01233 116.04785   0.938  0.35033
## ---
## Signif. codes:  0 '***' 0.001 '**' 0.01 '*' 0.05 '.' 0.1 ' ' 1
##
## Correlation of Fixed Effects:
##             (Intr) SmplTD FMT0_C FMT0_F FMT10_ BrdJrs BrdJrC
## SmplTypDrrh -0.113
## FMTdy0_Cntr -0.357 -0.204
## FMTday0_FMT -0.410 -0.086  0.444
## FMTdy10_FMT -0.407 -0.098  0.446  0.601
## BreedJersey -0.389 -0.022  0.004  0.048  0.049
## BrdJrsyCrss -0.256 -0.035  0.007  0.064  0.065  0.434
## SubGroup    -0.621  0.014 -0.003 -0.056 -0.058 -0.067 -0.144
#Plotting shannon results model
#install.packages("sjPlot")
library("sjPlot")
p <- plot_model(shanonn)
p
```

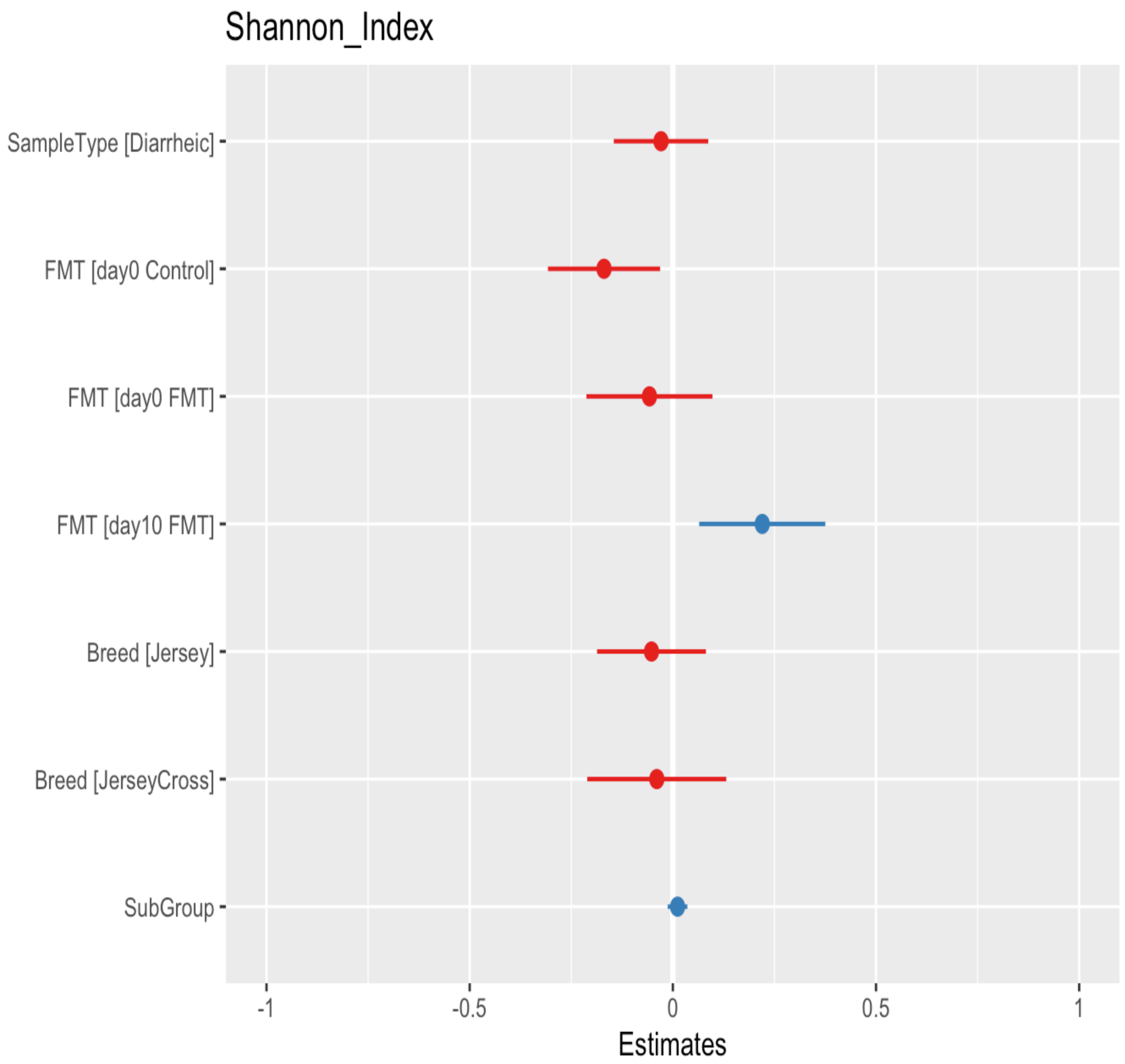


```
#Plotting the empirical shannon results (not modeled)

bxp <- ggboxplot(Shannon_Index, x = "FMT", y = "Shannon_Index",color = "SampleType", p
alette = "aaas",add = "jitter",bxp.errorbar=T)

bxp
```

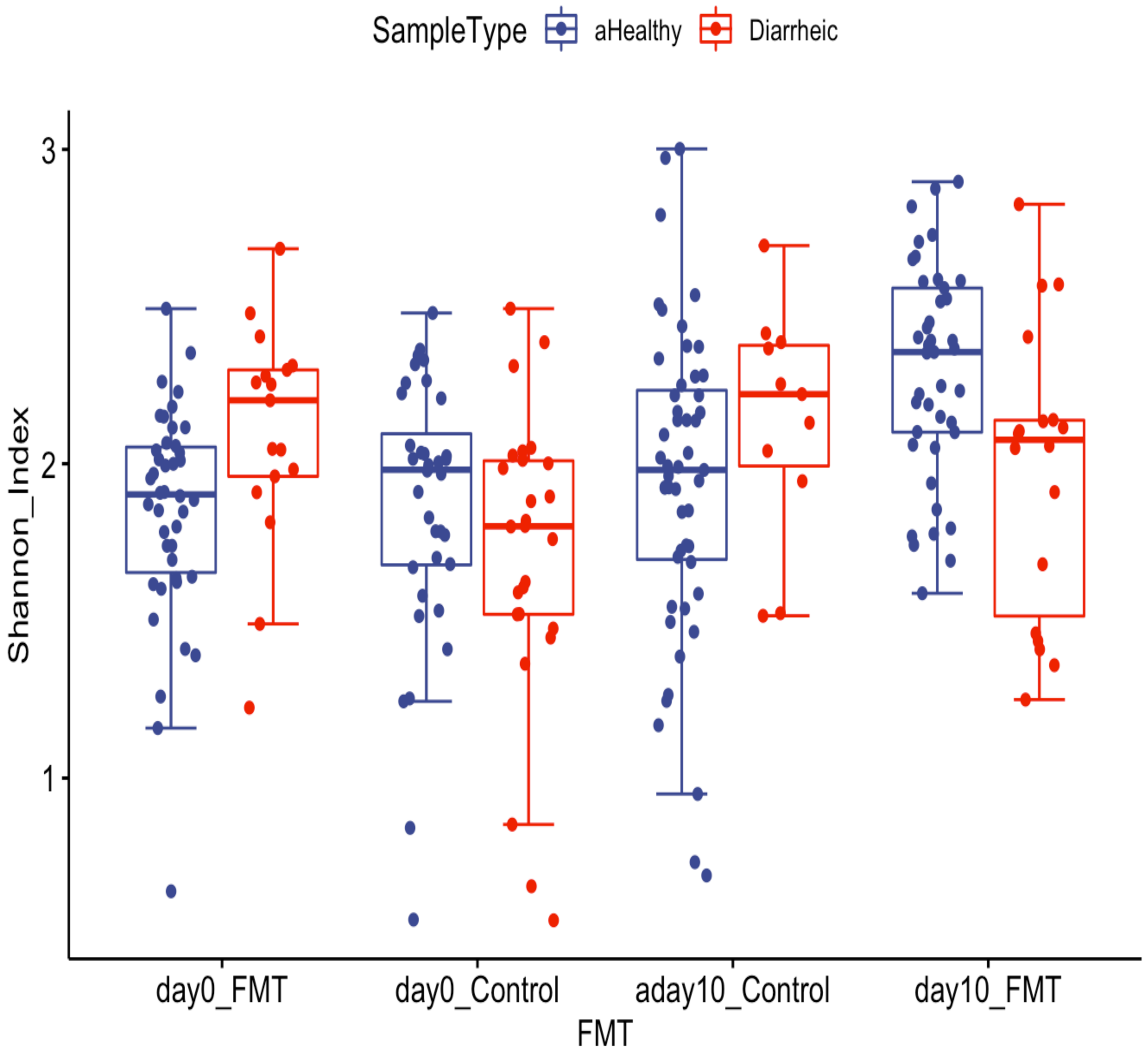


```
#Plotting the diversity for the FMT product
Shannon_Index_Product <- diversity(analysisData2[243:245,2:81], "shannon")
Shannon_Index_Product
## [1] 2.455659 2.473634 2.518141
```

#Making phyloseq obkects

```
library("phyloseq")
library("ggplot2")
library("readxl")
```

```
library("dplyr")
library("readxl")
otu_mat <- read_excel("otu_mat.xlsx")
otu_mat<- as.data.frame(otu_mat)

tax_mat <- read_excel("tax_mat.xlsx")
tax_mat<- as.data.frame(tax_mat)

samplescalves <- read_excel("samples.xlsx")
samples_df <- as.data.frame(samplescalves)
```

#Define the row names from the otu column

```
row.names(otu_mat) <- otu_mat$otu
```

#Remove the column otu since it is now used as a row name

```
otu_mat$otu <- NULL
```

#Idem for the two other matrixes

```
  row.names(tax_mat) <- tax_mat$otu
  tax_mat$otu <- NULL
```

#Transform into matrixes otu and tax tables (sample table can be left as data frame)

```
  otu_mat <- as.matrix(otu_mat)
  colnames(otu_mat) <- as.character(otu_mat[1,]) # I added this to make zymo id as hea
der
  class(otu_mat) <- "numeric" # I added this to change to numeric
  otu_mat = otu_mat[-1,] #Deleting 1st row that is NA
  tax_mat <- as.matrix(tax_mat)
```

#Transform to phyloseq objects

```
  OTU = otu_table(otu_mat, taxa_are_rows = TRUE)
  TAX = tax_table(tax_mat)
    row.names(samples_df) <- samples_df$sample
  samples_df$sample <- NULL
  meta.df <- samples_df
  samples = sample_data(meta.df)
```

```
dta <- phyloseq(OTU, TAX, samples)
dta
## phyloseq-class experiment-level object
## otu_table()   OTU Table:         [ 82 taxa and 245 samples ]
## sample_data() Sample Data:       [ 245 samples by 10 sample variables ]
## tax_table()   Taxonomy Table:    [ 82 taxa by 2 taxonomic ranks ]
```

#Visualize data

```
  sample_names(dta)
##   [1] "13V3V4"     "14V3V4"     "238V3V4"    "239V3V4"    "240V3V4"
##   [6] "241V3V4"    "242V3V4"    "243V3V4"    "244V3V4"    "245V3V4"
##  [11] "246V3V4"    "247V3V4"    "248V3V4"    "249V3V4"    "250V3V4"
##  [16] "251V3V4"    "252V3V4"    "253V3V4"    "254V3V4"    "255V3V4"
##  [21] "256V3V4"    "257V3V4"    "258V3V4"    "259V3V4"    "260V3V4"
##  [26] "261V3V4"    "262V3V4"    "263V3V4"    "264V3V4"    "265V3V4"
##  [31] "266V3V4"    "267V3V4"    "268V3V4"    "269V3V4"    "26V3V4"
##  [36] "270V3V4"    "271V3V4"    "272V3V4"    "273V3V4"    "274V3V4"
##  [41] "275V3V4"    "276V3V4"    "277V3V4"    "278V3V4"    "279V3V4"
##  [46] "280V3V4"    "281V3V4"    "282V3V4"    "283V3V4"    "284V3V4"
##  [51] "285V3V4"    "286V3V4"    "287V3V4"    "288V3V4"    "289V3V4"
##  [56] "290V3V4"    "291V3V4"    "292V3V4"    "293V3V4"    "294V3V4"
##  [61] "295V3V4"    "296V3V4"    "297V3V4"    "298V3V4"    "299V3V4"
##  [66] "300V3V4"    "301V3V4"    "302V3V4"    "303V3V4"    "304V3V4"
##  [71] "305V3V4"    "306V3V4"    "307V3V4"    "308V3V4"    "309V3V4"
##  [76] "30V3V4"     "310V3V4"    "311V3V4"    "312V3V4"    "313V3V4"
##  [81] "314V3V4"    "315V3V4"    "316V3V4"    "317V3V4"    "318V3V4"
##  [86] "319V3V4"    "320V3V4"    "321V3V4"    "322V3V4"    "323V3V4"
##  [91] "324V3V4"    "325V3V4"    "326V3V4"    "327V3V4"    "328V3V4"
##  [96] "329V3V4"    "330V3V4"    "331V3V4"    "332V3V4"    "333V3V4"
## [101] "334V3V4"    "335V3V4"    "336V3V4"    "337V3V4"    "338V3V4"
## [106] "339V3V4"    "340V3V4"    "341V3V4"    "342V3V4"    "343V3V4"
## [111] "344V3V4"    "345V3V4"    "346V3V4"    "347V3V4"    "348V3V4"
## [116] "349V3V4"    "350V3V4"    "351V3V4"    "352V3V4"    "353V3V4"
## [121] "354V3V4"    "355V3V4"    "356V3V4"    "357V3V4"    "358V3V4"
## [126] "359V3V4"    "360V3V4"    "361V3V4"    "362V3V4"    "363V3V4"
## [131] "364V3V4"    "365V3V4"    "366V3V4"    "367V3V4"    "368V3V4"
## [136] "369V3V4"    "370V3V4"    "371V3V4"    "372V3V4"    "373V3V4"
```

```
## [141] "374V3V4"      "375V3V4"      "376V3V4"      "377V3V4"      "378V3V4"
## [146] "379V3V4"      "380V3V4"      "381V3V4"      "382V3V4"      "383V3V4"
## [151] "384V3V4"      "385V3V4"      "386V3V4"      "387V3V4"      "388V3V4"
## [156] "389V3V4"      "38V3V4"       "390V3V4"      "391V3V4"      "392V3V4"
## [161] "393V3V4"      "394V3V4"      "395V3V4"      "396V3V4"      "397V3V4"
## [166] "398V3V4"      "399V3V4"      "39V3V4"       "400V3V4"      "401V3V4"
## [171] "402V3V4"      "403V3V4"      "404V3V4"      "405V3V4"      "406V3V4"
## [176] "407V3V4"      "408V3V4"      "409V3V4"      "410V3V4"      "411V3V4"
## [181] "412V3V4"      "413V3V4"      "414V3V4"      "415V3V4"      "416V3V4"
## [186] "417V3V4"      "418V3V4"      "419V3V4"      "420V3V4"      "421V3V4"
## [191] "422V3V4"      "423V3V4"      "424V3V4"      "425V3V4"      "426V3V4"
## [196] "427V3V4"      "428V3V4"      "429V3V4"      "430V3V4"      "431V3V4"
## [201] "432V3V4"      "433V3V4"      "434V3V4"      "435V3V4"      "436V3V4"
## [206] "437V3V4"      "438V3V4"      "439V3V4"      "43V3V4"       "440V3V4"
## [211] "441V3V4"      "442V3V4"      "443V3V4"      "444V3V4"      "445V3V4"
## [216] "446V3V4"      "447V3V4"      "448V3V4"      "449V3V4"      "450V3V4"
## [221] "451V3V4"      "452V3V4"      "453V3V4"      "454V3V4"      "455V3V4"
## [226] "456V3V4"      "457V3V4"      "458V3V4"      "459V3V4"      "460V3V4"
## [231] "461V3V4"      "462V3V4"      "463V3V4"      "46V3V4"       "49V3V4"
## [236] "50V3V4"       "61V3V4"       "64V3V4"       "72V3V4"       "73V3V4"
## [241] "77V3V4"       "9V3V4"        "FMT_Product1" "FMT_Product2" "FMT_Product3"
  rank_names(dta)
## [1] "Genus"   "Species"
  sample_variables(dta)
##  [1] "Animal"        "Died"          "HealthOutcome" "FMT"
##  [5] "Breed"         "SampleAge"     "AgeWeek"       "SampleType"
##  [9] "SampleTime"    "FarmID"
#Making a Venn diagram
library("phyloseq")
library("eulerr")
library("microbiome")
library("devtools")
pseq <- subset_samples(dta, SampleTime =="2sample")
# simple way to count number of samples in each group
table(meta(pseq)$FMT, useNA = "always")
##
## day10_Control     day10_FMT       Product          <NA>
```

```
##            62            59             3            0
group_states <- unique(as.character(meta(pseq)$FMT))
print(group_states)
## [1] "day10_Control" "day10_FMT"     "Product"
list_core <- c() # an empty object to store information

for (n in group_states){
    ps.sub <- subset_samples(pseq, FMT == n)
    core_m <- core_members(ps.sub,
                           detection = 0.001,
                           prevalence = 0.00) # The data is already filtered and we wa
nt to consider all 81 ASVs
    print(paste0("No. of core taxa in ", n, " : ", length(core_m)))
    list_core[[n]] <- core_m # add to a list core taxa for each group.
    print(list_core)
}
## [1] "No. of core taxa in day10_Control : 81"
## $day10_Control
##  [1] "Bifidobacterium_longum"              "Faecalibacterium_prausnitzii"
##  [3] "Lactobacillus_uncultured"            "Uncultured"
##  [5] "Ruminococcus_torques"                "Butyricicoccus_pullicaecorum"
##  [7] "Clostridium_nexile"                  "Collinsella_aerofaciens"
##  [9] "Lactobacillus_johnsonii"             "Blautia_uncultured"
## [11] "Ruminococcus_gnavus"                 "Streptococcus_uncultured"
## [13] "Bacteroides_fragilis"                "Bacteroides_vulgatus"
## [15] "Blautia_luti"                        "Bifidobacterium_bifidum"
## [17] "Escherichia/Shigella_uncultured"     "Bifidobacterium_uncultured"
## [19] "Gallibacterium_anatis"               "Clostridium_uncultured"
## [21] "Veillonella_magna"                   "Veillonella_caviae"
## [23] "Megasphaera_elsdenii"                "Prevotella_stercorea"
## [25] "Dorea_formicigenerans"               "Alloprevotella_rava"
## [27] "Prevotella_copri"                    "Gemmiger_formicilis"
## [29] "Lachnoclostridium_urinimassiliense"  "Blautia_schinkii"
## [31] "Clostridium_ramosum"                 "Flavonifractor_plautii"
## [33] "Streptococcus_pleomorphus"           "Akkermansia_muciniphila"
## [35] "Ruminococcus_bromii"                 "Pseudoflavonifractor_capillosus"
## [37] "Clostridium_lactatifermentans"       "Enterococcus_cecorum"
```

```
## [39] "Phascolarctobacterium_succinatutens" "Lactobacillus_salivarius"
## [41] "Coprococcus_comes"                   "Ruminococcus_obeum"
## [43] "Sutterella_stercoricanis"            "Sharpea_azabuensis"
## [45] "Bacteroides_xylanisolvens"           "Prevotella_oris"
## [47] "Lactobacillus_reuteri"               "Barnesiella_intestinihominis"
## [49] "Clostridium_cadaveris"               "Clostridium_saccharolyticum"
## [51] "Eubacterium_biforme"                 "Butyrivibrio_fibrisolvens"
## [53] "Parasutterella_excrementihominis"    "Clostridium_oroticum"
## [55] "Bacteroides_uniformis"               "Bifidobacterium_pseudolongum"
## [57] "Eubacterium_coprostanoligenes"       "Howardella_ureilytica"
## [59] "Ruminococcus_lactaris"               "Clostridium_hylemonae"
## [61] "Prevotella_fusca"                    "Parabacteroides_johnsonii"
## [63] "Clostridium_leptum"                  "Blautia_glucerasea"
## [65] "Eubacterium_limosum"                 "Anaerostipes_caccae"
## [67] "Intestinimonas_massiliensis"         "Anaerophaga_thermohalophila"
## [69] "Eggerthella_lenta"                   "Alkaliphilus_crotonatoxidans"
## [71] "Oscillibacter_valericigenes"         "Parabacteroides_merdae"
## [73] "Pedobacter_alluvionis"               "Intestinimonas_butyriciproducens"
## [75] "Olsenella_umbonata"                  "Clostridium_methylpentosum"
## [77] "Vallitalea_guaymasensis"             "Prevotella_baroniae"
## [79] "Solobacterium_moorei"                "Prevotella_buccae"
## [81] "Others"
##
## [1] "No. of core taxa in day10_FMT : 82"
## $day10_Control
##  [1] "Bifidobacterium_longum"              "Faecalibacterium_prausnitzii"
##  [3] "Lactobacillus_uncultured"            "Uncultured"
##  [5] "Ruminococcus_torques"                "Butyricicoccus_pullicaecorum"
##  [7] "Clostridium_nexile"                  "Collinsella_aerofaciens"
##  [9] "Lactobacillus_johnsonii"             "Blautia_uncultured"
## [11] "Ruminococcus_gnavus"                 "Streptococcus_uncultured"
## [13] "Bacteroides_fragilis"                "Bacteroides_vulgatus"
## [15] "Blautia_luti"                        "Bifidobacterium_bifidum"
## [17] "Escherichia/Shigella_uncultured"     "Bifidobacterium_uncultured"
## [19] "Gallibacterium_anatis"               "Clostridium_uncultured"
## [21] "Veillonella_magna"                   "Veillonella_caviae"
## [23] "Megasphaera_elsdenii"                "Prevotella_stercorea"
```

```
## [25] "Dorea_formicigenerans"                 "Alloprevotella_rava"
## [27] "Prevotella_copri"                      "Gemmiger_formicilis"
## [29] "Lachnoclostridium_urinimassiliense"    "Blautia_schinkii"
## [31] "Clostridium_ramosum"                   "Flavonifractor_plautii"
## [33] "Streptococcus_pleomorphus"             "Akkermansia_muciniphila"
## [35] "Ruminococcus_bromii"                   "Pseudoflavonifractor_capillosus"
## [37] "Clostridium_lactatifermentans"         "Enterococcus_cecorum"
## [39] "Phascolarctobacterium_succinatutens" "Lactobacillus_salivarius"
## [41] "Coprococcus_comes"                     "Ruminococcus_obeum"
## [43] "Sutterella_stercoricanis"              "Sharpea_azabuensis"
## [45] "Bacteroides_xylanisolvens"             "Prevotella_oris"
## [47] "Lactobacillus_reuteri"                 "Barnesiella_intestinihominis"
## [49] "Clostridium_cadaveris"                 "Clostridium_saccharolyticum"
## [51] "Eubacterium_biforme"                   "Butyrivibrio_fibrisolvens"
## [53] "Parasutterella_excrementihominis"      "Clostridium_oroticum"
## [55] "Bacteroides_uniformis"                 "Bifidobacterium_pseudolongum"
## [57] "Eubacterium_coprostanoligenes"         "Howardella_ureilytica"
## [59] "Ruminococcus_lactaris"                 "Clostridium_hylemonae"
## [61] "Prevotella_fusca"                      "Parabacteroides_johnsonii"
## [63] "Clostridium_leptum"                    "Blautia_glucerasea"
## [65] "Eubacterium_limosum"                   "Anaerostipes_caccae"
## [67] "Intestinimonas_massiliensis"           "Anaerophaga_thermohalophila"
## [69] "Eggerthella_lenta"                     "Alkaliphilus_crotonatoxidans"
## [71] "Oscillibacter_valericigenes"           "Parabacteroides_merdae"
## [73] "Pedobacter_alluvionis"                 "Intestinimonas_butyriciproducens"
## [75] "Olsenella_umbonata"                    "Clostridium_methylpentosum"
## [77] "Vallitalea_guaymasensis"               "Prevotella_baroniae"
## [79] "Solobacterium_moorei"                  "Prevotella_buccae"
## [81] "Others"
##
## $day10_FMT
##  [1] "Bifidobacterium_longum"                "Faecalibacterium_prausnitzii"
##  [3] "Lactobacillus_uncultured"              "Uncultured"
##  [5] "Ruminococcus_torques"                  "Butyricicoccus_pullicaecorum"
##  [7] "Clostridium_nexile"                    "Collinsella_aerofaciens"
##  [9] "Lactobacillus_johnsonii"               "Blautia_uncultured"
## [11] "Ruminococcus_gnavus"                   "Streptococcus_uncultured"
```

```
## [13] "Bacteroides_fragilis"                "Bacteroides_vulgatus"
## [15] "Blautia_luti"                        "Bifidobacterium_bifidum"
## [17] "Escherichia/Shigella_uncultured"     "Bifidobacterium_uncultured"
## [19] "Gallibacterium_anatis"               "Clostridium_uncultured"
## [21] "Veillonella_magna"                   "Veillonella_caviae"
## [23] "Megasphaera_elsdenii"                "Prevotella_stercorea"
## [25] "Dorea_formicigenerans"               "Alloprevotella_rava"
## [27] "Prevotella_copri"                    "Gemmiger_formicilis"
## [29] "Lachnoclostridium_urinimassiliense"  "Blautia_schinkii"
## [31] "Clostridium_ramosum"                 "Flavonifractor_plautii"
## [33] "Streptococcus_pleomorphus"           "Akkermansia_muciniphila"
## [35] "Ruminococcus_bromii"                 "Pseudoflavonifractor_capillosus"
## [37] "Clostridium_lactatifermentans"       "Enterococcus_cecorum"
## [39] "Phascolarctobacterium_succinatutens" "Lactobacillus_salivarius"
## [41] "Coprococcus_comes"                   "Ruminococcus_obeum"
## [43] "Sutterella_stercoricanis"            "Sharpea_azabuensis"
## [45] "Bacteroides_xylanisolvens"           "Prevotella_oris"
## [47] "Lactobacillus_reuteri"               "Barnesiella_intestinihominis"
## [49] "Clostridium_cadaveris"               "Clostridium_saccharolyticum"
## [51] "Eubacterium_biforme"                 "Butyrivibrio_fibrisolvens"
## [53] "Parasutterella_excrementihominis"    "Clostridium_oroticum"
## [55] "Bacteroides_uniformis"               "Bifidobacterium_pseudolongum"
## [57] "Eubacterium_coprostanoligenes"       "Howardella_ureilytica"
## [59] "Ruminococcus_lactaris"               "Clostridium_hylemonae"
## [61] "Prevotella_fusca"                    "Parabacteroides_johnsonii"
## [63] "Clostridium_leptum"                  "Blautia_glucerasea"
## [65] "Eubacterium_limosum"                 "Anaerostipes_caccae"
## [67] "Intestinimonas_massiliensis"         "Anaerophaga_thermohalophila"
## [69] "Eggerthella_lenta"                   "Alkaliphilus_crotonatoxidans"
## [71] "Oribacterium_sinus"                  "Oscillibacter_valericigenes"
## [73] "Parabacteroides_merdae"              "Pedobacter_alluvionis"
## [75] "Intestinimonas_butyriciproducens"    "Olsenella_umbonata"
## [77] "Clostridium_methylpentosum"          "Vallitalea_guaymasensis"
## [79] "Prevotella_baroniae"                 "Solobacterium_moorei"
## [81] "Prevotella_buccae"                   "Others"
##
## [1] "No. of core taxa in Product : 56"
```

```
## $day10_Control
##  [1] "Bifidobacterium_longum"               "Faecalibacterium_prausnitzii"
##  [3] "Lactobacillus_uncultured"             "Uncultured"
##  [5] "Ruminococcus_torques"                 "Butyricicoccus_pullicaecorum"
##  [7] "Clostridium_nexile"                   "Collinsella_aerofaciens"
##  [9] "Lactobacillus_johnsonii"              "Blautia_uncultured"
## [11] "Ruminococcus_gnavus"                  "Streptococcus_uncultured"
## [13] "Bacteroides_fragilis"                 "Bacteroides_vulgatus"
## [15] "Blautia_luti"                         "Bifidobacterium_bifidum"
## [17] "Escherichia/Shigella_uncultured"      "Bifidobacterium_uncultured"
## [19] "Gallibacterium_anatis"                "Clostridium_uncultured"
## [21] "Veillonella_magna"                    "Veillonella_caviae"
## [23] "Megasphaera_elsdenii"                 "Prevotella_stercorea"
## [25] "Dorea_formicigenerans"                "Alloprevotella_rava"
## [27] "Prevotella_copri"                     "Gemmiger_formicilis"
## [29] "Lachnoclostridium_urinimassiliense"   "Blautia_schinkii"
## [31] "Clostridium_ramosum"                  "Flavonifractor_plautii"
## [33] "Streptococcus_pleomorphus"            "Akkermansia_muciniphila"
## [35] "Ruminococcus_bromii"                  "Pseudoflavonifractor_capillosus"
## [37] "Clostridium_lactatifermentans"        "Enterococcus_cecorum"
## [39] "Phascolarctobacterium_succinatutens" "Lactobacillus_salivarius"
## [41] "Coprococcus_comes"                    "Ruminococcus_obeum"
## [43] "Sutterella_stercoricanis"             "Sharpea_azabuensis"
## [45] "Bacteroides_xylanisolvens"            "Prevotella_oris"
## [47] "Lactobacillus_reuteri"                "Barnesiella_intestinihominis"
## [49] "Clostridium_cadaveris"                "Clostridium_saccharolyticum"
## [51] "Eubacterium_biforme"                  "Butyrivibrio_fibrisolvens"
## [53] "Parasutterella_excrementihominis"     "Clostridium_oroticum"
## [55] "Bacteroides_uniformis"                "Bifidobacterium_pseudolongum"
## [57] "Eubacterium_coprostanoligenes"        "Howardella_ureilytica"
## [59] "Ruminococcus_lactaris"                "Clostridium_hylemonae"
## [61] "Prevotella_fusca"                     "Parabacteroides_johnsonii"
## [63] "Clostridium_leptum"                   "Blautia_glucerasea"
## [65] "Eubacterium_limosum"                  "Anaerostipes_caccae"
## [67] "Intestinimonas_massiliensis"          "Anaerophaga_thermohalophila"
## [69] "Eggerthella_lenta"                    "Alkaliphilus_crotonatoxidans"
## [71] "Oscillibacter_valericigenes"          "Parabacteroides_merdae"
```

```
## [73] "Pedobacter_alluvionis"                 "Intestinimonas_butyriciproducens"
## [75] "Olsenella_umbonata"                    "Clostridium_methylpentosum"
## [77] "Vallitalea_guaymasensis"               "Prevotella_baroniae"
## [79] "Solobacterium_moorei"                  "Prevotella_buccae"
## [81] "Others"
##
## $day10_FMT
##  [1] "Bifidobacterium_longum"                "Faecalibacterium_prausnitzii"
##  [3] "Lactobacillus_uncultured"              "Uncultured"
##  [5] "Ruminococcus_torques"                  "Butyricicoccus_pullicaecorum"
##  [7] "Clostridium_nexile"                    "Collinsella_aerofaciens"
##  [9] "Lactobacillus_johnsonii"               "Blautia_uncultured"
## [11] "Ruminococcus_gnavus"                   "Streptococcus_uncultured"
## [13] "Bacteroides_fragilis"                  "Bacteroides_vulgatus"
## [15] "Blautia_luti"                          "Bifidobacterium_bifidum"
## [17] "Escherichia/Shigella_uncultured"       "Bifidobacterium_uncultured"
## [19] "Gallibacterium_anatis"                 "Clostridium_uncultured"
## [21] "Veillonella_magna"                     "Veillonella_caviae"
## [23] "Megasphaera_elsdenii"                  "Prevotella_stercorea"
## [25] "Dorea_formicigenerans"                 "Alloprevotella_rava"
## [27] "Prevotella_copri"                      "Gemmiger_formicilis"
## [29] "Lachnoclostridium_urinimassiliense"    "Blautia_schinkii"
## [31] "Clostridium_ramosum"                   "Flavonifractor_plautii"
## [33] "Streptococcus_pleomorphus"             "Akkermansia_muciniphila"
## [35] "Ruminococcus_bromii"                   "Pseudoflavonifractor_capillosus"
## [37] "Clostridium_lactatifermentans"         "Enterococcus_cecorum"
## [39] "Phascolarctobacterium_succinatutens"   "Lactobacillus_salivarius"
## [41] "Coprococcus_comes"                     "Ruminococcus_obeum"
## [43] "Sutterella_stercoricanis"              "Sharpea_azabuensis"
## [45] "Bacteroides_xylanisolvens"             "Prevotella_oris"
## [47] "Lactobacillus_reuteri"                 "Barnesiella_intestinihominis"
## [49] "Clostridium_cadaveris"                 "Clostridium_saccharolyticum"
## [51] "Eubacterium_biforme"                   "Butyrivibrio_fibrisolvens"
## [53] "Parasutterella_excrementihominis"      "Clostridium_oroticum"
## [55] "Bacteroides_uniformis"                 "Bifidobacterium_pseudolongum"
## [57] "Eubacterium_coprostanoligenes"         "Howardella_ureilytica"
## [59] "Ruminococcus_lactaris"                 "Clostridium_hylemonae"
```

```
## [61] "Prevotella_fusca"                    "Parabacteroides_johnsonii"
## [63] "Clostridium_leptum"                  "Blautia_glucerasea"
## [65] "Eubacterium_limosum"                 "Anaerostipes_caccae"
## [67] "Intestinimonas_massiliensis"         "Anaerophaga_thermohalophila"
## [69] "Eggerthella_lenta"                   "Alkaliphilus_crotonatoxidans"
## [71] "Oribacterium_sinus"                  "Oscillibacter_valericigenes"
## [73] "Parabacteroides_merdae"              "Pedobacter_alluvionis"
## [75] "Intestinimonas_butyriciproducens"    "Olsenella_umbonata"
## [77] "Clostridium_methylpentosum"          "Vallitalea_guaymasensis"
## [79] "Prevotella_baroniae"                 "Solobacterium_moorei"
## [81] "Prevotella_buccae"                   "Others"
##
## $Product
##  [1] "Bifidobacterium_longum"              "Faecalibacterium_prausnitzii"
##  [3] "Lactobacillus_uncultured"            "Uncultured"
##  [5] "Ruminococcus_torques"                "Butyricicoccus_pullicaecorum"
##  [7] "Collinsella_aerofaciens"             "Lactobacillus_johnsonii"
##  [9] "Ruminococcus_gnavus"                 "Bacteroides_fragilis"
## [11] "Bacteroides_vulgatus"                "Bifidobacterium_bifidum"
## [13] "Gallibacterium_anatis"               "Veillonella_magna"
## [15] "Megasphaera_elsdenii"                "Prevotella_stercorea"
## [17] "Dorea_formicigenerans"               "Alloprevotella_rava"
## [19] "Prevotella_copri"                    "Lachnoclostridium_urinimassiliense"
## [21] "Blautia_schinkii"                    "Clostridium_ramosum"
## [23] "Flavonifractor_plautii"              "Akkermansia_muciniphila"
## [25] "Ruminococcus_bromii"                 "Pseudoflavonifractor_capillosus"
## [27] "Enterococcus_cecorum"                "Phascolarctobacterium_succinatutens"
## [29] "Lactobacillus_salivarius"            "Coprococcus_comes"
## [31] "Sharpea_azabuensis"                  "Bacteroides_xylanisolvens"
## [33] "Prevotella_oris"                     "Lactobacillus_reuteri"
## [35] "Barnesiella_intestinihominis"        "Clostridium_cadaveris"
## [37] "Parasutterella_excrementihominis"    "Bacteroides_uniformis"
## [39] "Bifidobacterium_pseudolongum"        "Eubacterium_coprostanoligenes"
## [41] "Howardella_ureilytica"               "Ruminococcus_lactaris"
## [43] "Prevotella_fusca"                    "Parabacteroides_johnsonii"
## [45] "Blautia_glucerasea"                  "Eubacterium_limosum"
## [47] "Anaerostipes_caccae"                 "Intestinimonas_massiliensis"
```

```
## [49] "Anaerophaga_thermohalophila"          "Eggerthella_lenta"
## [51] "Oribacterium_sinus"                   "Intestinimonas_butyriciproducens"
## [53] "Olsenella_umbonata"                   "Prevotella_baroniae"
## [55] "Solobacterium_moorei"                 "Others"
print(list_core)
## $day10_Control
##  [1] "Bifidobacterium_longum"               "Faecalibacterium_prausnitzii"
##  [3] "Lactobacillus_uncultured"             "Uncultured"
##  [5] "Ruminococcus_torques"                 "Butyricicoccus_pullicaecorum"
##  [7] "Clostridium_nexile"                   "Collinsella_aerofaciens"
##  [9] "Lactobacillus_johnsonii"              "Blautia_uncultured"
## [11] "Ruminococcus_gnavus"                  "Streptococcus_uncultured"
## [13] "Bacteroides_fragilis"                 "Bacteroides_vulgatus"
## [15] "Blautia_luti"                         "Bifidobacterium_bifidum"
## [17] "Escherichia/Shigella_uncultured"      "Bifidobacterium_uncultured"
## [19] "Gallibacterium_anatis"                "Clostridium_uncultured"
## [21] "Veillonella_magna"                    "Veillonella_caviae"
## [23] "Megasphaera_elsdenii"                 "Prevotella_stercorea"
## [25] "Dorea_formicigenerans"                "Alloprevotella_rava"
## [27] "Prevotella_copri"                     "Gemmiger_formicilis"
## [29] "Lachnoclostridium_urinimassiliense"   "Blautia_schinkii"
## [31] "Clostridium_ramosum"                  "Flavonifractor_plautii"
## [33] "Streptococcus_pleomorphus"            "Akkermansia_muciniphila"
## [35] "Ruminococcus_bromii"                  "Pseudoflavonifractor_capillosus"
## [37] "Clostridium_lactatifermentans"        "Enterococcus_cecorum"
## [39] "Phascolarctobacterium_succinatutens"  "Lactobacillus_salivarius"
## [41] "Coprococcus_comes"                    "Ruminococcus_obeum"
## [43] "Sutterella_stercoricanis"             "Sharpea_azabuensis"
## [45] "Bacteroides_xylanisolvens"            "Prevotella_oris"
## [47] "Lactobacillus_reuteri"                "Barnesiella_intestinihominis"
## [49] "Clostridium_cadaveris"                "Clostridium_saccharolyticum"
## [51] "Eubacterium_biforme"                  "Butyrivibrio_fibrisolvens"
## [53] "Parasutterella_excrementihominis"     "Clostridium_oroticum"
## [55] "Bacteroides_uniformis"                "Bifidobacterium_pseudolongum"
## [57] "Eubacterium_coprostanoligenes"        "Howardella_ureilytica"
## [59] "Ruminococcus_lactaris"                "Clostridium_hylemonae"
## [61] "Prevotella_fusca"                     "Parabacteroides_johnsonii"
```

```
## [63] "Clostridium_leptum"                    "Blautia_glucerasea"
## [65] "Eubacterium_limosum"                   "Anaerostipes_caccae"
## [67] "Intestinimonas_massiliensis"           "Anaerophaga_thermohalophila"
## [69] "Eggerthella_lenta"                     "Alkaliphilus_crotonatoxidans"
## [71] "Oscillibacter_valericigenes"           "Parabacteroides_merdae"
## [73] "Pedobacter_alluvionis"                 "Intestinimonas_butyriciproducens"
## [75] "Olsenella_umbonata"                    "Clostridium_methylpentosum"
## [77] "Vallitalea_guaymasensis"               "Prevotella_baroniae"
## [79] "Solobacterium_moorei"                  "Prevotella_buccae"
## [81] "Others"
##
## $day10_FMT
##  [1] "Bifidobacterium_longum"                "Faecalibacterium_prausnitzii"
##  [3] "Lactobacillus_uncultured"              "Uncultured"
##  [5] "Ruminococcus_torques"                  "Butyricicoccus_pullicaecorum"
##  [7] "Clostridium_nexile"                    "Collinsella_aerofaciens"
##  [9] "Lactobacillus_johnsonii"               "Blautia_uncultured"
## [11] "Ruminococcus_gnavus"                   "Streptococcus_uncultured"
## [13] "Bacteroides_fragilis"                  "Bacteroides_vulgatus"
## [15] "Blautia_luti"                          "Bifidobacterium_bifidum"
## [17] "Escherichia/Shigella_uncultured"       "Bifidobacterium_uncultured"
## [19] "Gallibacterium_anatis"                 "Clostridium_uncultured"
## [21] "Veillonella_magna"                     "Veillonella_caviae"
## [23] "Megasphaera_elsdenii"                  "Prevotella_stercorea"
## [25] "Dorea_formicigenerans"                 "Alloprevotella_rava"
## [27] "Prevotella_copri"                      "Gemmiger_formicilis"
## [29] "Lachnoclostridium_urinimassiliense"    "Blautia_schinkii"
## [31] "Clostridium_ramosum"                   "Flavonifractor_plautii"
## [33] "Streptococcus_pleomorphus"             "Akkermansia_muciniphila"
## [35] "Ruminococcus_bromii"                   "Pseudoflavonifractor_capillosus"
## [37] "Clostridium_lactatifermentans"         "Enterococcus_cecorum"
## [39] "Phascolarctobacterium_succinatutens" "Lactobacillus_salivarius"
## [41] "Coprococcus_comes"                     "Ruminococcus_obeum"
## [43] "Sutterella_stercoricanis"              "Sharpea_azabuensis"
## [45] "Bacteroides_xylanisolvens"             "Prevotella_oris"
## [47] "Lactobacillus_reuteri"                 "Barnesiella_intestinihominis"
## [49] "Clostridium_cadaveris"                 "Clostridium_saccharolyticum"
```

```
## [51] "Eubacterium_biforme"                  "Butyrivibrio_fibrisolvens"
## [53] "Parasutterella_excrementihominis"     "Clostridium_oroticum"
## [55] "Bacteroides_uniformis"                "Bifidobacterium_pseudolongum"
## [57] "Eubacterium_coprostanoligenes"        "Howardella_ureilytica"
## [59] "Ruminococcus_lactaris"                "Clostridium_hylemonae"
## [61] "Prevotella_fusca"                     "Parabacteroides_johnsonii"
## [63] "Clostridium_leptum"                   "Blautia_glucerasea"
## [65] "Eubacterium_limosum"                  "Anaerostipes_caccae"
## [67] "Intestinimonas_massiliensis"          "Anaerophaga_thermohalophila"
## [69] "Eggerthella_lenta"                    "Alkaliphilus_crotonatoxidans"
## [71] "Oribacterium_sinus"                   "Oscillibacter_valericigenes"
## [73] "Parabacteroides_merdae"               "Pedobacter_alluvionis"
## [75] "Intestinimonas_butyriciproducens"     "Olsenella_umbonata"
## [77] "Clostridium_methylpentosum"           "Vallitalea_guaymasensis"
## [79] "Prevotella_baroniae"                  "Solobacterium_moorei"
## [81] "Prevotella_buccae"                    "Others"
##
## $Product
##  [1] "Bifidobacterium_longum"               "Faecalibacterium_prausnitzii"
##  [3] "Lactobacillus_uncultured"             "Uncultured"
##  [5] "Ruminococcus_torques"                 "Butyricicoccus_pullicaecorum"
##  [7] "Collinsella_aerofaciens"              "Lactobacillus_johnsonii"
##  [9] "Ruminococcus_gnavus"                  "Bacteroides_fragilis"
## [11] "Bacteroides_vulgatus"                 "Bifidobacterium_bifidum"
## [13] "Gallibacterium_anatis"                "Veillonella_magna"
## [15] "Megasphaera_elsdenii"                 "Prevotella_stercorea"
## [17] "Dorea_formicigenerans"                "Alloprevotella_rava"
## [19] "Prevotella_copri"                     "Lachnoclostridium_urinimassiliense"
## [21] "Blautia_schinkii"                     "Clostridium_ramosum"
## [23] "Flavonifractor_plautii"               "Akkermansia_muciniphila"
## [25] "Ruminococcus_bromii"                  "Pseudoflavonifractor_capillosus"
## [27] "Enterococcus_cecorum"                 "Phascolarctobacterium_succinatutens"
## [29] "Lactobacillus_salivarius"             "Coprococcus_comes"
## [31] "Sharpea_azabuensis"                   "Bacteroides_xylanisolvens"
## [33] "Prevotella_oris"                      "Lactobacillus_reuteri"
## [35] "Barnesiella_intestinihominis"         "Clostridium_cadaveris"
## [37] "Parasutterella_excrementihominis"     "Bacteroides_uniformis"
```

```
## [39] "Bifidobacterium_pseudolongum"        "Eubacterium_coprostanoligenes"
## [41] "Howardella_ureilytica"               "Ruminococcus_lactaris"
## [43] "Prevotella_fusca"                    "Parabacteroides_johnsonii"
## [45] "Blautia_glucerasea"                  "Eubacterium_limosum"
## [47] "Anaerostipes_caccae"                 "Intestinimonas_massiliensis"
## [49] "Anaerophaga_thermohalophila"         "Eggerthella_lenta"
## [51] "Oribacterium_sinus"                  "Intestinimonas_butyriciproducens"
## [53] "Olsenella_umbonata"                  "Prevotella_baroniae"
## [55] "Solobacterium_moorei"                "Others"
# Specify colors and plot venn
# supplying colors in the order they appear in list_core
mycols <- c(nonCRC="#d6e2e9", CRC="#cbf3f0", H="#fcf5c7")
plot(venn(list_core),
     fills = mycols)
```

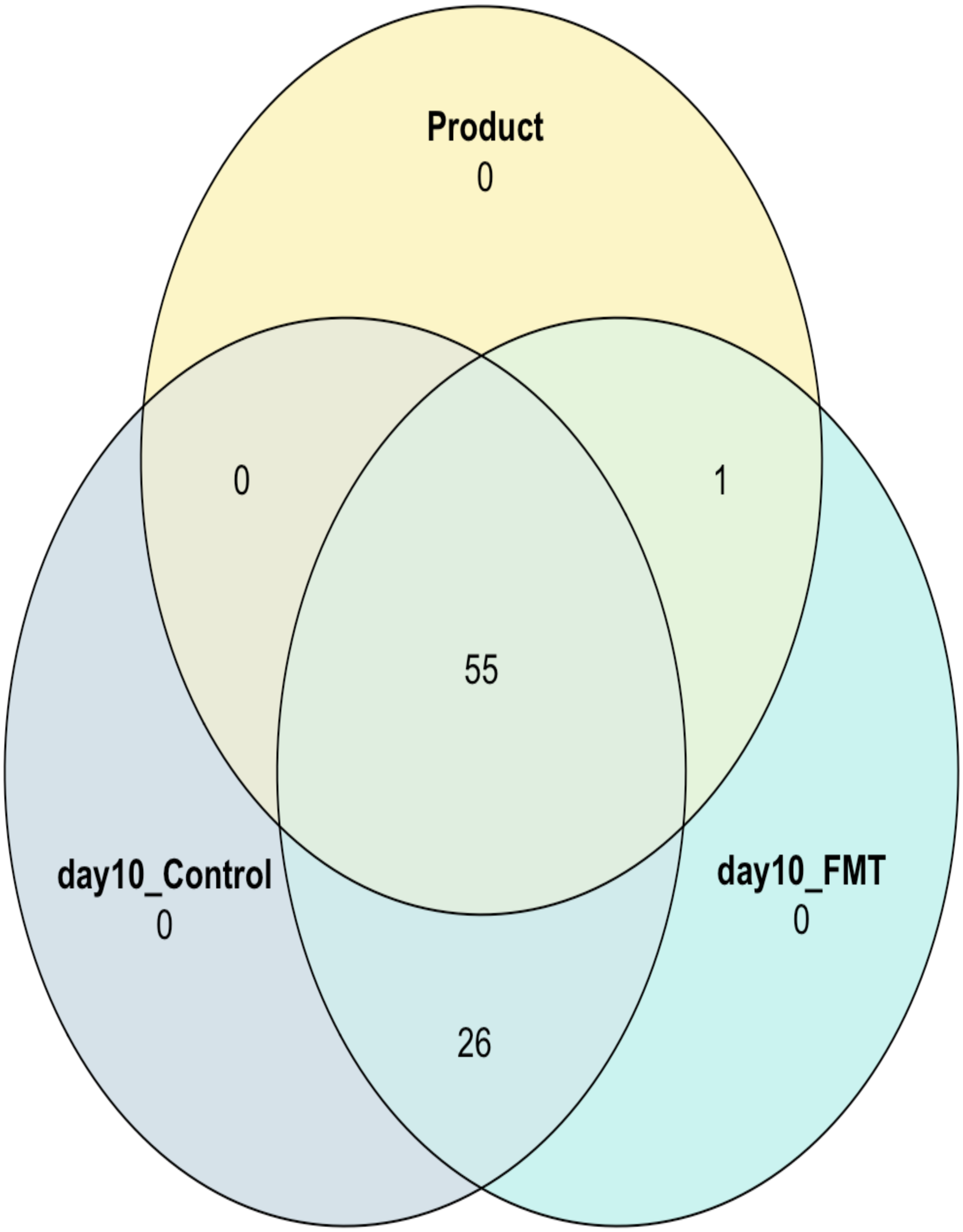


```
##Making a PCoA
library("ggplot2")
library("phyloseq")
library("tidyverse")
ordu = ordinate(dta, "PCoA", "altGower", weighted=TRUE)
p = plot_ordination(dta, ordu, color="FMT", shape = "SampleType")
p = p + geom_point(size=5, alpha=0.6)
p = p + scale_colour_brewer(type="qual", palette="Set1")
```

```
p + ggtitle("PCoA altGower") + theme_bw() + theme(text = element_text(size=20))
```

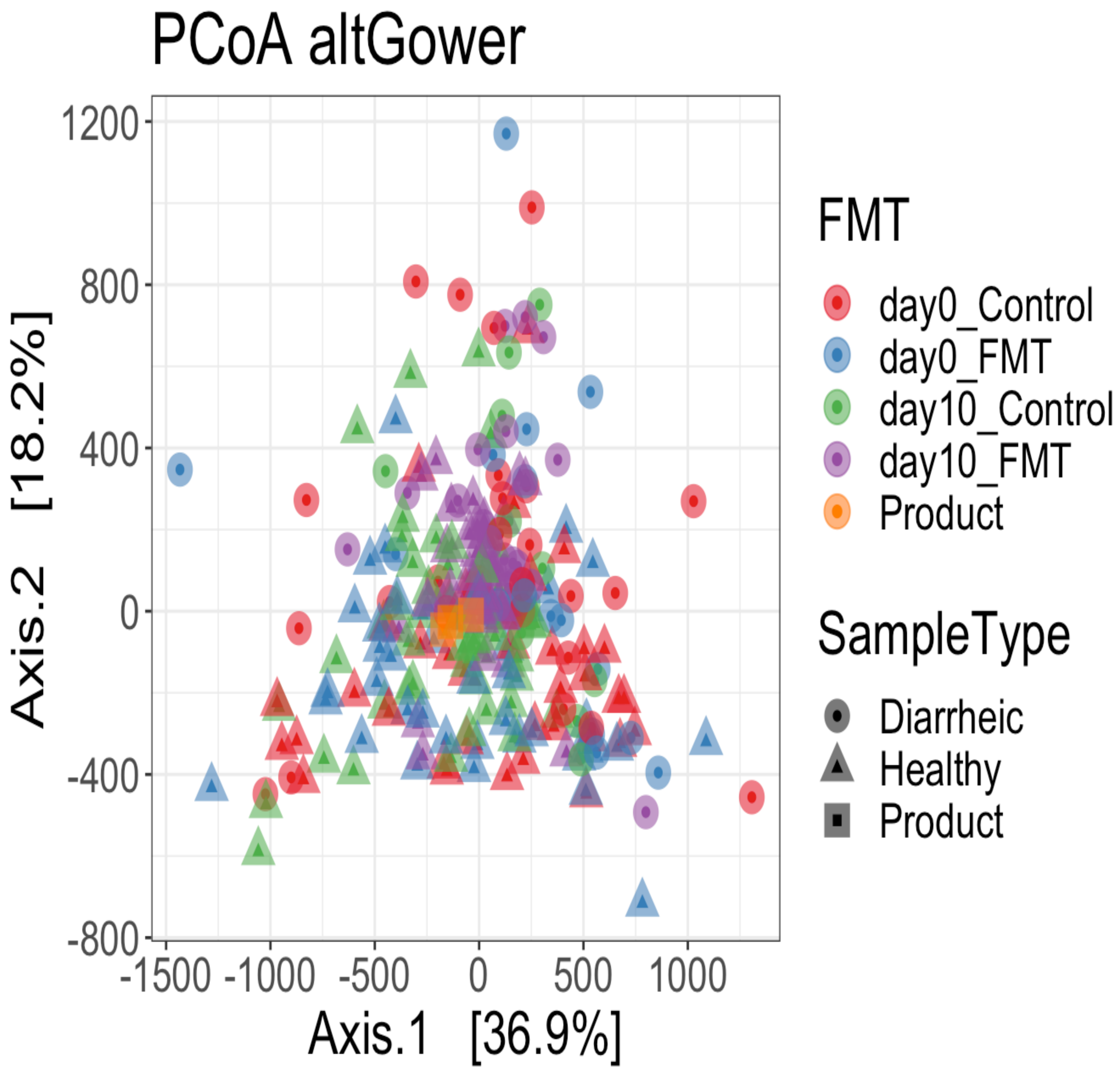


```
#PERMANOVA
# Calculate distance matrix
dta_altGower <- phyloseq::distance(dta, method = "altGower")

# make a data frame from the sample_data
sample_altGower <- data.frame(sample_data(dta))
```

```
#We use the vegan package to calculate distances and run PERMANOVA
library("vegan")
# Adonis test
test <- adonis2(dta_altGower ~ FMT + SampleType, data = sample_altGower, permutations
= 999)
test
## Permutation test for adonis under reduced model
## Terms added sequentially (first to last)
## Permutation: free
## Number of permutations: 999
##
## adonis2(formula = dta_altGower ~ FMT + SampleType, data = sample_altGower, permutat
ions = 999)
##              Df  SumOfSqs       R2       F Pr(>F)
## FMT           4  -1240398 -0.01087 -0.6640  1.000
## SampleType    1   3734040  0.03272  7.9959  0.001 ***
## Residual    239 111611238  0.97815
## Total       244 114104880  1.00000
## ---
## Signif. codes:  0 '***' 0.001 '**' 0.01 '*' 0.05 '.' 0.1 ' ' 1
#install.packages("RVAideMemoire")
library("RVAideMemoire")
test <- adonis(dta_altGower ~ FMT + SampleType, data = sample_altGower, permutations =
999)
test
##
## Call:
## adonis(formula = dta_altGower ~ FMT + SampleType, data = sample_altGower,      perm
utations = 999)
##
## Permutation: free
## Number of permutations: 999
##
## Terms added sequentially (first to last)
##
##              Df SumsOfSqs MeanSqs F.Model       R2 Pr(>F)
## FMT           4  -1240398 -310099 -0.6640 -0.01087  1.000
## SampleType    1   3734040 3734040  7.9959  0.03272  0.001 ***
```

```
## Residuals  239 111611238  466993          0.97815
## Total      244 114104880                  1.00000
## ---
## Signif. codes:  0 '***' 0.001 '**' 0.01 '*' 0.05 '.' 0.1 ' ' 1
pairwise.perm.manova(dta_altGower,sample_altGower$FMT,nperm=999)
##
##  Pairwise comparisons using permutation MANOVAs on a distance matrix
##
## data:  dta_altGower by sample_altGower$FMT
## 999 permutations
##
##              day0_Control day0_FMT day10_Control day10_FMT
## day0_FMT      1.00         -        -             -
## day10_Control 1.00         1.00     -             -
## day10_FMT     1.00         1.00     0.10          -
## Product       1.00         1.00     1.00          0.47
##
## P value adjustment method: fdr
library("devtools")
#install_github("pmartinezarbizu/pairwiseAdonis/pairwiseAdonis")
library("pairwiseAdonis")
pairwise_org <- pairwise.adonis(dta_altGower,sample_altGower$FMT, p.adjust.m = "bonfer
roni", perm = 999)
pairwise_org
##                            pairs Df   SumsOfSqs    F.Model            R2
## 1       day0_FMT vs day0_Control  1   524862.27  0.7572918  0.0063235546
## 2      day0_FMT vs day10_Control  1   -57582.61 -0.1147036 -0.0009648258
## 3          day0_FMT vs day10_FMT  1 -1410368.81 -3.1441150 -0.0278595577
## 4            day0_FMT vs Product  1  -349437.12 -0.5293007 -0.0089001929
## 5  day0_Control vs day10_Control  1  -370297.76 -0.7147598 -0.0058932135
## 6      day0_Control vs day10_FMT  1 -2103909.14 -4.5110800 -0.0394018915
## 7        day0_Control vs Product  1  -395154.11 -0.5779168 -0.0092582100
## 8     day10_Control vs day10_FMT  1   961684.29  3.4929534  0.0285155453
## 9       day10_Control vs Product  1   122382.16  0.3790634  0.0059808925
## 10          day10_FMT vs Product  1   475912.84  2.2599816  0.0362991047
##    p.value p.adjusted sig
## 1    0.586       1.00
```

```
## 2    0.971       1.00
## 3    1.000       1.00
## 4    1.000       1.00
## 5    0.997       1.00
## 6    1.000       1.00
## 7    0.998       1.00
## 8    0.006       0.06
## 9    0.807       1.00
## 10   0.103       1.00
# Homogeneity of dispersion test
beta <- betadisper(dta_altGower, sample_altGower$FMT)
permutest(beta)
##
## Permutation test for homogeneity of multivariate dispersions
## Permutation: free
## Number of permutations: 999
##
## Response: Distances
##             Df   Sum Sq Mean Sq      F N.Perm Pr(>F)
## Groups      4  6857403 1714351 27.238    999  0.001 ***
## Residuals 240 15105258   62939
## ---
## Signif. codes:  0 '***' 0.001 '**' 0.01 '*' 0.05 '.' 0.1 ' ' 1
#Rel Abund Heatmap
#install.packages("remotes")
#remotes::install_github("MadsAlbertsen/ampvis2")
library("ampvis2")

#Transforming phyloseq object to ampvis2
devtools::source_gist("8d0ca4206a66be7ff6d76fc4ab8e66c6")
data <- phyloseq_to_ampvis2(dta)

amp_heatmap(
  data = data,
  tax_aggregate = "Species",
  group_by = "SampleType",
  facet_by = "FMT",
```

```
  tax_show = 15)
## Warning: Transformation introduced infinite values in discrete y-axis
```

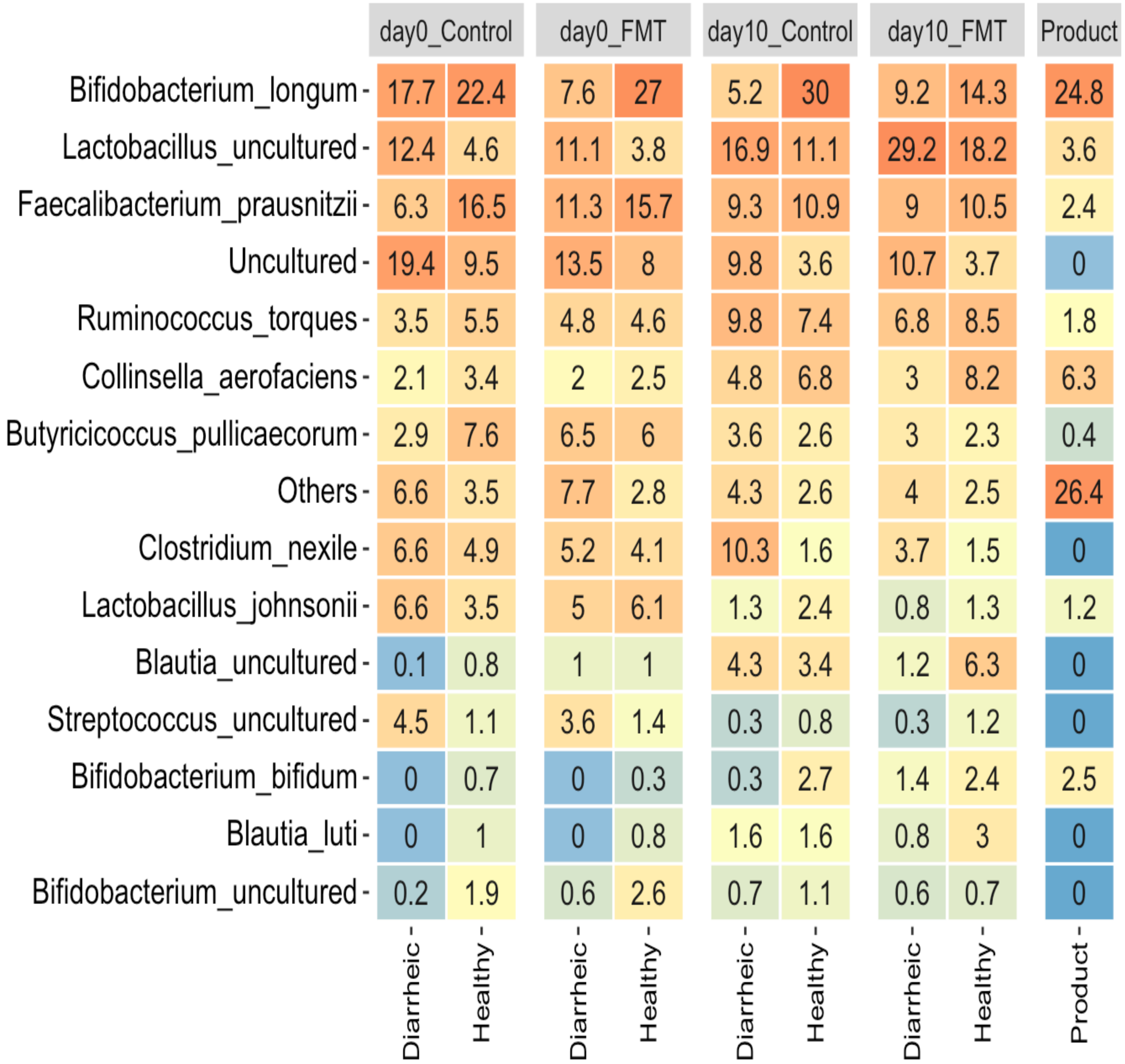


```
library(openxlsx)
fmt <- read.xlsx("modelData.xlsx")


fmt$Breed <- as.factor(fmt$Breed)
fmt$FMT <- factor(fmt$FMT, levels = c("Control","FMT"))
fmt$SampleType_day0 <- factor(fmt$SampleType_day0, levels = c("Healthy", "Diarrheic"))
```

```
fmt$SubGroup <- factor(fmt$SubGroup, levels = c("1", "2", "3", "4", "5", "6", "7", "8"
, "9"))


speciesNames <- unname(sapply(colnames(fmt[,(1:21)*2]), function(x) strsplit(x,"_day")
[[1]][[1]]))

Nreps <- 1000


allModels <- list()


for(s in speciesNames){


  betaVals <- data.frame()


  yVals0 <- fmt[,paste(s,"day0",sep="_")]

  yVals10 <- fmt[,paste(s,"day10",sep="_")]


  for(i in 1:Nreps){

    y0 <- yVals0 + runif(length(yVals0))

    y10 <- yVals10 + runif(length(yVals10))

    m <- glm(log(y10)  - log(y0) ~ FMT + SubGroup + Breed + FMT:SampleType_day0, data
= fmt, offset = log(TotalReads_day10/TotalReads_day0))

    modelCoeffs <- as.vector(t((summary(m))$coefficients[,1:2]))

    betaVals <- rbind(betaVals, data.frame(t(modelCoeffs)))

  }


  res <- data.frame(matrix(apply(betaVals,2,median), ncol = 2, byrow =  T))

  res$t <- apply(res, 1, function(x) x[1]/x[2])

  res$p_value <- apply(res, 1, function(x) 2*(1 - pt(abs(x[3]), m$df.residual)))

  rownames(res) <- rownames(summary(m)$coefficients)

  colnames(res) <- colnames(summary(m)$coefficients)


  allModels[[s]] <- res

  print(paste("Finished modeling species",s))


}

## [1] "Finished modeling species Bifidobacterium_longum"

## [1] "Finished modeling species Faecalibacterium_prausnitzii"

## [1] "Finished modeling species Lactobacillus_uncultured"
```

```
## [1] "Finished modeling species Ruminococcus_torques"
## [1] "Finished modeling species Collinsella_aerofaciens"
## [1] "Finished modeling species Lactobacillus_johnsonii"
## [1] "Finished modeling species Ruminococcus_gnavus"
## [1] "Finished modeling species Streptococcus_uncultured"
## [1] "Finished modeling species Bacteroides_fragilis"
## [1] "Finished modeling species Bacteroides_vulgatus"
## [1] "Finished modeling species Bifidobacterium_bifidum"
## [1] "Finished modeling species Clostridium_nexile"
## [1] "Finished modeling species Butyricicoccus_pullicaecorum"
## [1] "Finished modeling species Blautia_uncultured"
## [1] "Finished modeling species Blautia_luti"
## [1] "Finished modeling species Clostridium_uncultured"
## [1] "Finished modeling species Escherichia/Shigella_uncultured"
## [1] "Finished modeling species Lactobacillus_reuteri"
## [1] "Finished modeling species Lactobacillus_salivarius"
## [1] "Finished modeling species Bifidobacterium_pseudolongum"
## [1] "Finished modeling species Others"
allModels
## $Bifidobacterium_longum
##                                        Estimate Std. Error    t value
## (Intercept)                           3.8632156  0.8086196  4.7775436
## FMTFMT                               -0.4386509  0.5855428 -0.7491355
## SubGroup2                            -3.2594621  1.0041466 -3.2460021
## SubGroup3                            -4.4162976  1.0125793 -4.3614340
## SubGroup4                            -3.5927024  1.0020182 -3.5854661
## SubGroup5                            -4.7727865  0.9202042 -5.1866601
## SubGroup6                            -5.5472066  0.9635591 -5.7569968
## SubGroup7                            -5.0496304  1.1812214 -4.2749230
## SubGroup8                            -6.3825755  0.8785990 -7.2644923
## SubGroup9                            -3.9663987  1.1745492 -3.3769542
## BreedJersey                           0.3960453  0.5430566  0.7292892
## BreedJerseyCross                      0.4863516  0.6902706  0.7045812
## FMTControl:SampleType_day0Diarrheic  1.7416620  0.6844520  2.5446078
## FMTFMT:SampleType_day0Diarrheic      1.1123083  0.7731591  1.4386539
##                                          Pr(>|t|)
## (Intercept)                          5.676170e-06
```

```
## FMTFMT                              4.554193e-01
## SubGroup2                           1.562859e-03
## SubGroup3                           2.984382e-05
## SubGroup4                           5.085616e-04
## SubGroup5                           1.021240e-06
## SubGroup6                           8.274498e-08
## SubGroup7                           4.165655e-05
## SubGroup8                           6.362999e-11
## SubGroup9                           1.022690e-03
## BreedJersey                         4.674182e-01
## BreedJerseyCross                    4.826017e-01
## FMTControl:SampleType_day0Diarrheic 1.236679e-02
## FMTFMT:SampleType_day0Diarrheic     1.531673e-01
##
## $Faecalibacterium_prausnitzii
##                                       Estimate Std. Error    t value   Pr(>|t|)
## (Intercept)                         -1.5190947  1.0373935 -1.4643380 0.14603352
## FMTFMT                              -0.2782202  0.7512039 -0.3703657 0.71184214
## SubGroup2                           -1.0270914  1.2882388 -0.7972834 0.42705217
## SubGroup3                           -0.8059447  1.2990571 -0.6204074 0.53630876
## SubGroup4                            0.4069148  1.2855082  0.3165400 0.75220937
## SubGroup5                            0.7785633  1.1805474  0.6594934 0.51099592
## SubGroup6                            2.4136669  1.2361682  1.9525392 0.05348661
## SubGroup7                            0.9222856  1.5154113  0.6086042 0.54407671
## SubGroup8                            1.6934431  1.1271713  1.5023831 0.13594378
## SubGroup9                            1.3024857  1.5068514  0.8643757 0.38931520
## BreedJersey                          1.0429376  0.6966976  1.4969731 0.13734431
## BreedJerseyCross                     0.7887223  0.8855612  0.8906468 0.37511696
## FMTControl:SampleType_day0Diarrheic  1.1628480  0.8780965  1.3242827 0.18823098
## FMTFMT:SampleType_day0Diarrheic      1.5653531  0.9919005  1.5781353 0.11748646
##
## $Lactobacillus_uncultured
##                                        Estimate Std. Error     t value
## (Intercept)                          1.41404178  0.7219341  1.95868528
## FMTFMT                               1.86213924  0.5227715  3.56205176
## SubGroup2                            0.49418665  0.8965003  0.55123981
## SubGroup3                           -1.10869428  0.9040289 -1.22639248
```

```
## SubGroup4                           -1.79781829  0.8946001 -2.00963355
## SubGroup5                           -0.99256723  0.8215566 -1.20815434
## SubGroup6                           -1.04844247  0.8602638 -1.21874532
## SubGroup7                           -0.30601345  1.0545923 -0.29017228
## SubGroup8                           -0.66060317  0.7844116 -0.84216397
## SubGroup9                            1.21660574  1.0486354  1.16017997
## BreedJersey                          0.03418799  0.4848399  0.07051397
## BreedJerseyCross                    -0.29277930  0.6162723 -0.47508104
## FMTControl:SampleType_day0Diarrheic -0.75319505  0.6110775 -1.23256873
## FMTFMT:SampleType_day0Diarrheic     -1.18796098  0.6902750 -1.72099656
##                                         Pr(>|t|)
## (Intercept)                         0.0527522101
## FMTFMT                              0.0005508371
## SubGroup2                           0.5826185626
## SubGroup3                           0.2227430187
## SubGroup4                           0.0469870872
## SubGroup5                           0.2296513193
## SubGroup6                           0.2256210500
## SubGroup7                           0.7722460459
## SubGroup8                           0.4015745964
## SubGroup9                           0.2485576712
## BreedJersey                         0.9439162042
## BreedJerseyCross                    0.6356975303
## FMTControl:SampleType_day0Diarrheic 0.2204380952
## FMTFMT:SampleType_day0Diarrheic     0.0881417469
##
## $Ruminococcus_torques
##                                        Estimate Std. Error     t value
## (Intercept)                          2.03773236  1.1037105  1.84625616
## FMTFMT                              -0.20222354  0.7992259 -0.25302428
## SubGroup2                            0.17058478  1.3705915  0.12446070
## SubGroup3                            0.87396609  1.3821015  0.63234583
## SubGroup4                           -0.15604480  1.3676864 -0.11409399
## SubGroup5                           -0.43371845  1.2560158 -0.34531288
## SubGroup6                            0.04883009  1.3151923  0.03712772
## SubGroup7                           -2.47079171  1.6122864 -1.53247693
## SubGroup8                           -1.18367047  1.1992275 -0.98702742
```

```
## SubGroup9                           0.79641303  1.6031793  0.49677103
## BreedJersey                        -0.42063652  0.7412351 -0.56748057
## BreedJerseyCross                   -1.64125434  0.9421722 -1.74198986
## FMTControl:SampleType_day0Diarrheic  1.37325073  0.9342302  1.46992754
## FMTFMT:SampleType_day0Diarrheic      0.47791115  1.0553093  0.45286357
##                                       Pr(>|t|)
## (Intercept)                         0.06761906
## FMTFMT                              0.80073493
## SubGroup2                           0.90118403
## SubGroup3                           0.52850985
## SubGroup4                           0.90937710
## SubGroup5                           0.73053708
## SubGroup6                           0.97045235
## SubGroup7                           0.12835621
## SubGroup8                           0.32585590
## SubGroup9                           0.62036965
## BreedJersey                         0.57157646
## BreedJerseyCross                    0.08438378
## FMTControl:SampleType_day0Diarrheic 0.14451573
## FMTFMT:SampleType_day0Diarrheic     0.65156393
##
## $Collinsella_aerofaciens
##                                       Estimate Std. Error    t value
## (Intercept)                          6.4624389  1.0470539  6.1720212
## FMTFMT                              -0.3346251  0.7581993 -0.4413419
## SubGroup2                           -0.3682701  1.3002351 -0.2832334
## SubGroup3                           -3.0484257  1.3111542 -2.3249940
## SubGroup4                           -3.6762242  1.2974791 -2.8333590
## SubGroup5                           -3.4062573  1.1915410 -2.8586993
## SubGroup6                           -3.5826523  1.2476797 -2.8714519
## SubGroup7                           -2.9924511  1.5295231 -1.9564602
## SubGroup8                           -4.1675538  1.1376678 -3.6632433
## SubGroup9                           -4.2097157  1.5208835 -2.7679408
## BreedJersey                         -1.1360705  0.7031854 -1.6156060
## BreedJerseyCross                    -1.3212381  0.8938078 -1.4782128
## FMTControl:SampleType_day0Diarrheic  0.6209481  0.8862735  0.7006281
## FMTFMT:SampleType_day0Diarrheic      0.6849549  1.0011373  0.6841768
```

```
##                                                 Pr(>|t|)
## (Intercept)                              0.00000001229934
## FMTFMT                                   0.65985607207010
## SubGroup2                                0.77754514788122
## SubGroup3                                0.02195937967869
## SubGroup4                                0.00550631887346
## SubGroup5                                0.00511413393414
## SubGroup6                                0.00492660366448
## SubGroup7                                0.05301709551101
## SubGroup8                                0.00038909531565
## SubGroup9                                0.00664932013579
## BreedJersey                              0.10912441012117
## BreedJerseyCross                         0.14228853784528
## FMTControl:SampleType_day0Diarrheic 0.48505591133974
## FMTFMT:SampleType_day0Diarrheic     0.49534268100602
##
## $Lactobacillus_johnsonii
##                                        Estimate Std. Error     t value
## (Intercept)                          1.34959590  1.1553378  1.16813968
## FMTFMT                               0.07249159  0.8366105  0.08664915
## SubGroup2                            0.77169558  1.4347024  0.53787850
## SubGroup3                           -0.34346808  1.4467508 -0.23740653
## SubGroup4                           -2.86098362  1.4316614 -1.99836611
## SubGroup5                           -2.64777364  1.3147673 -2.01387239
## SubGroup6                           -3.71500697  1.3767118 -2.69846379
## SubGroup7                           -2.78754817  1.6877028 -1.65168186
## SubGroup8                           -2.79492313  1.2553227 -2.22645787
## SubGroup9                           -2.44826546  1.6781698 -1.45889023
## BreedJersey                          0.22990052  0.7759072  0.29629899
## BreedJerseyCross                     0.13559360  0.9862433  0.13748494
## FMTControl:SampleType_day0Diarrheic  0.55184307  0.9779299  0.56429720
## FMTFMT:SampleType_day0Diarrheic      0.94614367  1.1046726  0.85649241
##                                        Pr(>|t|)
## (Intercept)                          0.245346633
## FMTFMT                               0.931112342
## SubGroup2                            0.591777949
## SubGroup3                            0.812795396
```

```
## SubGroup4                            0.048213557
## SubGroup5                            0.046532626
## SubGroup6                            0.008096651
## SubGroup7                            0.101531290
## SubGroup8                            0.028079001
## SubGroup9                            0.147524690
## BreedJersey                          0.767576083
## BreedJerseyCross                     0.890905874
## FMTControl:SampleType_day0Diarrheic 0.573732869
## FMTFMT:SampleType_day0Diarrheic     0.393639561
##
## $Ruminococcus_gnavus
##                                        Estimate Std. Error     t value
## (Intercept)                         -0.08126956  1.2792224 -0.06353043
## FMTFMT                              -0.31169152  0.9263187 -0.33648412
## SubGroup2                           -0.31733533  1.5885428 -0.19976505
## SubGroup3                            0.15790237  1.6018831  0.09857297
## SubGroup4                            0.88576270  1.5851757  0.55877887
## SubGroup5                           -0.05136076  1.4557473 -0.03528137
## SubGroup6                           -0.28427344  1.5243340 -0.18649026
## SubGroup7                           -1.00098870  1.8686720 -0.53566849
## SubGroup8                            0.77816546  1.3899286  0.55986004
## SubGroup9                            1.86240374  1.8581167  1.00230720
## BreedJersey                         -0.60428913  0.8591062 -0.70339280
## BreedJerseyCross                    -1.19248369  1.0919963 -1.09202178
## FMTControl:SampleType_day0Diarrheic -0.87776533  1.0827914 -0.81065045
## FMTFMT:SampleType_day0Diarrheic     -0.38856327  1.2231245 -0.31768088
##                                      Pr(>|t|)
## (Intercept)                         0.9494627
## FMTFMT                              0.7371648
## SubGroup2                           0.8420435
## SubGroup3                           0.9216618
## SubGroup4                           0.5774802
## SubGroup5                           0.9719211
## SubGroup6                           0.8524134
## SubGroup7                           0.5932994
## SubGroup8                           0.5767451
```

```
## SubGroup9                           0.3184566
## BreedJersey                         0.4833387
## BreedJerseyCross                    0.2772763
## FMTControl:SampleType_day0Diarrheic 0.4193658
## FMTFMT:SampleType_day0Diarrheic     0.7513461
##
## $Streptococcus_uncultured
##                                        Estimate Std. Error     t value
## (Intercept)                          3.14930972  1.3483865  2.33561349
## FMTFMT                               0.04841592  0.9764022  0.04958604
## SubGroup2                           -0.72585106  1.6744310 -0.43349117
## SubGroup3                           -1.85360134  1.6884925 -1.09778474
## SubGroup4                           -2.17018145  1.6708818 -1.29882400
## SubGroup5                           -2.03532001  1.5344556 -1.32641178
## SubGroup6                           -3.13891112  1.6067506 -1.95357710
## SubGroup7                           -3.62441736  1.9697060 -1.84008038
## SubGroup8                           -3.25729992  1.4650782 -2.22329421
## SubGroup9                           -3.90281744  1.9585800 -1.99267706
## BreedJersey                          0.24571981  0.9055558  0.27134697
## BreedJerseyCross                     0.49573182  1.1510375  0.43068260
## FMTControl:SampleType_day0Diarrheic -0.39260875  1.1413350 -0.34399082
## FMTFMT:SampleType_day0Diarrheic     -1.44824124  1.2892555 -1.12331593
##                                       Pr(>|t|)
## (Intercept)                         0.02137586
## FMTFMT                              0.96054471
## SubGroup2                           0.66553066
## SubGroup3                           0.27476275
## SubGroup4                           0.19679636
## SubGroup5                           0.18752750
## SubGroup6                           0.05336199
## SubGroup7                           0.06852812
## SubGroup8                           0.02829804
## SubGroup9                           0.04884311
## BreedJersey                         0.78664700
## BreedJerseyCross                    0.66756549
## FMTControl:SampleType_day0Diarrheic 0.73152827
## FMTFMT:SampleType_day0Diarrheic     0.26381739
```

```
##
## $Bacteroides_fragilis
##                                    Estimate Std. Error     t value
## (Intercept)                      -2.5925827  1.2205439 -2.12412079
## FMTFMT                           -0.7791364  0.8838280 -0.88154757
## SubGroup2                         2.3108492  1.5156756  1.52463308
## SubGroup3                        -0.1206482  1.5284040 -0.07893737
## SubGroup4                         0.4606903  1.5124630  0.30459610
## SubGroup5                         0.6912090  1.3889715  0.49764085
## SubGroup6                         1.5653961  1.4544121  1.07630848
## SubGroup7                         0.8592428  1.7829551  0.48192058
## SubGroup8                         1.4043948  1.3261719  1.05898400
## SubGroup9                         0.7636984  1.7728840  0.43076615
## BreedJersey                       0.6427618  0.8196986  0.78414398
## BreedJerseyCross                  1.5067648  1.0419059  1.44616209
## FMTControl:SampleType_day0Diarrheic  0.7426889  1.0331233  0.71887730
## FMTFMT:SampleType_day0Diarrheic   1.3627490  1.1670192  1.16771773
##                                    Pr(>|t|)
## (Intercept)                      0.03596374
## FMTFMT                           0.37999750
## SubGroup2                        0.13030096
## SubGroup3                        0.93722988
## SubGroup4                        0.76126542
## SubGroup5                        0.61975837
## SubGroup6                        0.28421012
## SubGroup7                        0.63084664
## SubGroup8                        0.29199183
## SubGroup9                        0.66750492
## BreedJersey                      0.43468811
## BreedJerseyCross                 0.15105463
## FMTControl:SampleType_day0Diarrheic 0.47378352
## FMTFMT:SampleType_day0Diarrheic  0.24551611
##
## $Bacteroides_vulgatus
##                                    Estimate Std. Error    t value
## (Intercept)                       3.5923884  1.2104161  2.9678954
## FMTFMT                           -1.6811215  0.8764942 -1.9180065
```

```
## SubGroup2                              -1.6761381  1.5030989 -1.1151216
## SubGroup3                              -2.4624849  1.5157216 -1.6246288
## SubGroup4                              -0.9634913  1.4999129 -0.6423649
## SubGroup5                              -0.7199222  1.3774461 -0.5226499
## SubGroup6                              -1.4249947  1.4423437 -0.9879717
## SubGroup7                              -2.3660110  1.7681606 -1.3381200
## SubGroup8                              -1.8790893  1.3151676 -1.4287831
## SubGroup9                              -5.2589875  1.7581730 -2.9911661
## BreedJersey                             0.2664600  0.8128970  0.3277906
## BreedJerseyCross                        0.4350401  1.0332604  0.4210363
## FMTControl:SampleType_day0Diarrheic -1.2878956  1.0245506 -1.2570346
## FMTFMT:SampleType_day0Diarrheic     -0.4007486  1.1573355 -0.3462683
##                                        Pr(>|t|)
## (Intercept)                         0.003700179
## FMTFMT                              0.057775801
## SubGroup2                           0.267296595
## SubGroup3                           0.107183871
## SubGroup4                           0.522010281
## SubGroup5                           0.602298383
## SubGroup6                           0.325395370
## SubGroup7                           0.183694091
## SubGroup8                           0.155979360
## SubGroup9                           0.003449826
## BreedJersey                         0.743710485
## BreedJerseyCross                    0.674573099
## FMTControl:SampleType_day0Diarrheic 0.211478134
## FMTFMT:SampleType_day0Diarrheic     0.729821078
##
## $Bifidobacterium_bifidum
##                                        Estimate Std. Error    t value   Pr(>|t|)
## (Intercept)                             2.2013572  1.3233264  1.6635028 0.09913813
## FMTFMT                                  1.4357285  0.9582555  1.4982731 0.13700675
## SubGroup2                               1.2909918  1.6433113  0.7856039 0.43383575
## SubGroup3                              -0.5650352  1.6571115 -0.3409760 0.73379027
## SubGroup4                              -1.3168713  1.6398281 -0.8030545 0.42372351
## SubGroup5                               0.3901906  1.5059374  0.2591015 0.79605477
## SubGroup6                              -0.5421699  1.5768887 -0.3438225 0.73165448
```

```
## SubGroup7                         -0.1080171  1.9330985 -0.0558777 0.95554344
## SubGroup8                         -1.5781333  1.4378494 -1.0975651 0.27485825
## SubGroup9                         -0.5392388  1.9221793 -0.2805351 0.77960868
## BreedJersey                        1.8965502  0.8887258  2.1340105 0.03512607
## BreedJerseyCross                   0.7976444  1.1296452  0.7061017 0.48165953
## FMTControl:SampleType_day0Diarrheic  1.5790269  1.1201230  1.4096907 0.16153105
## FMTFMT:SampleType_day0Diarrheic     -0.9576414  1.2652944 -0.7568526 0.45080149
##
## $Clostridium_nexile
##                                        Estimate Std. Error     t value
## (Intercept)                         -1.37343757  1.2334115 -1.11352750
## FMTFMT                              -3.06405432  0.8931457 -3.43063203
## SubGroup2                           -0.94776963  1.5316546 -0.61878810
## SubGroup3                            0.45300120  1.5445171  0.29329633
## SubGroup4                            0.32726807  1.5284081  0.21412349
## SubGroup5                            0.90775578  1.4036147  0.64672718
## SubGroup6                            1.23411991  1.4697452  0.83968291
## SubGroup7                            0.42888738  1.8017519  0.23803909
## SubGroup8                            2.70711591  1.3401530  2.02000508
## SubGroup9                            2.73132097  1.7915746  1.52453656
## BreedJersey                          0.05713117  0.8283403  0.06897065
## BreedJerseyCross                    -0.36202540  1.0528901 -0.34383967
## FMTControl:SampleType_day0Diarrheic -1.83092651  1.0440149 -1.75373596
## FMTFMT:SampleType_day0Diarrheic      1.67009015  1.1793225  1.41614376
##                                         Pr(>|t|)
## (Intercept)                         0.2679771502
## FMTFMT                              0.0008566775
## SubGroup2                           0.5373711035
## SubGroup3                           0.7698637443
## SubGroup4                           0.8308582729
## SubGroup5                           0.5191934279
## SubGroup6                           0.4029584227
## SubGroup7                           0.8123060163
## SubGroup8                           0.0458817621
## SubGroup9                           0.1303250351
## BreedJersey                         0.9451417061
## BreedJerseyCross                    0.7316416187
```

```
## FMTControl:SampleType_day0Diarrheic 0.0823391540
## FMTFMT:SampleType_day0Diarrheic     0.1596379348
##
## $Butyricicoccus_pullicaecorum
##                                       Estimate Std. Error    t value
## (Intercept)                         -2.8471174  0.9697788 -2.9358422
## FMTFMT                              -0.9559055  0.7022423 -1.3612188
## SubGroup2                            0.7988438  1.2042746  0.6633402
## SubGroup3                            1.1541870  1.2143879  0.9504270
## SubGroup4                            1.6883490  1.2017220  1.4049414
## SubGroup5                            1.9433050  1.1036023  1.7608744
## SubGroup6                            2.4932537  1.1555979  2.1575444
## SubGroup7                            1.9312949  1.4166406  1.3632921
## SubGroup8                            3.6491365  1.0537051  3.4631478
## SubGroup9                            1.3710561  1.4086386  0.9733200
## BreedJersey                          0.2207104  0.6512886  0.3388827
## BreedJerseyCross                     0.1374830  0.8278426  0.1660738
## FMTControl:SampleType_day0Diarrheic  0.4337404  0.8208644  0.5283947
## FMTFMT:SampleType_day0Diarrheic      1.9841476  0.9272509  2.1398174
##                                         Pr(>|t|)
## (Intercept)                         0.0040724882
## FMTFMT                              0.1763043526
## SubGroup2                           0.5085393121
## SubGroup3                           0.3440372091
## SubGroup4                           0.1629352538
## SubGroup5                           0.0811165994
## SubGroup6                           0.0332003467
## SubGroup7                           0.1756522133
## SubGroup8                           0.0007688087
## SubGroup9                           0.3325895345
## BreedJersey                         0.7353622310
## BreedJerseyCross                    0.8684122762
## FMTControl:SampleType_day0Diarrheic 0.5983196621
## FMTFMT:SampleType_day0Diarrheic     0.0346421351
##
## $Blautia_uncultured
##                                        Estimate Std. Error     t value
```

```
## (Intercept)                       0.74667681  1.2155421  0.61427473
## FMTFMT                            0.99992510  0.8802061  1.13601252
## SubGroup2                        -1.86837713  1.5094644 -1.23777492
## SubGroup3                         0.02577344  1.5221405  0.01693236
## SubGroup4                         2.64544861  1.5062649  1.75629708
## SubGroup5                         1.13550031  1.3832795  0.82087554
## SubGroup6                         1.91681097  1.4484519  1.32335150
## SubGroup7                         0.43559168  1.7756486  0.24531413
## SubGroup8                        -0.25391365  1.3207372 -0.19225145
## SubGroup9                         1.80600592  1.7656187  1.02287424
## BreedJersey                       0.48523311  0.8163395  0.59440112
## BreedJerseyCross                 -0.28522544  1.0376361 -0.27488001
## FMTControl:SampleType_day0Diarrheic  0.79647958  1.0288895  0.77411575
## FMTFMT:SampleType_day0Diarrheic      0.51359526  1.1622367  0.44190244
##                                     Pr(>|t|)
## (Intercept)                       0.54033772
## FMTFMT                            0.25848935
## SubGroup2                         0.21850870
## SubGroup3                         0.98652211
## SubGroup4                         0.08189879
## SubGroup5                         0.41354228
## SubGroup6                         0.18853928
## SubGroup7                         0.80668310
## SubGroup8                         0.84790964
## SubGroup9                         0.30867435
## BreedJersey                       0.55349862
## BreedJerseyCross                  0.78393848
## FMTControl:SampleType_day0Diarrheic 0.44056944
## FMTFMT:SampleType_day0Diarrheic     0.65945164
##
## $Blautia_luti
##                                     Estimate Std. Error    t value   Pr(>|t|)
## (Intercept)                       -0.5117707  1.1985722 -0.4269836 0.67024922
## FMTFMT                             0.9910896  0.8679177  1.1419165 0.25603772
## SubGroup2                          0.4151932  1.4883911  0.2789544 0.78081827
## SubGroup3                          0.7374552  1.5008903  0.4913451 0.62418871
## SubGroup4                         -0.3519006  1.4852363 -0.2369324 0.81316223
```

```
## SubGroup5                           0.5882944  1.3639679  0.4313111 0.66710995
## SubGroup6                           1.7817005  1.4282304  1.2474881 0.21494198
## SubGroup7                           0.5540839  1.7508592  0.3164640 0.75226688
## SubGroup8                           1.0061742  1.3022988  0.7726140 0.44145417
## SubGroup9                           3.6003832  1.7409694  2.0680337 0.04104834
## BreedJersey                         1.3926673  0.8049428  1.7301445 0.08648770
## BreedJerseyCross                   -1.1534707  1.0231500 -1.1273721 0.26210695
## FMTControl:SampleType_day0Diarrheic  1.7375303  1.0145254  1.7126533 0.08967277
## FMTFMT:SampleType_day0Diarrheic     2.2354675  1.1460111  1.9506509 0.05371398
##
## $Clostridium_uncultured
##                                      Estimate Std. Error     t value
## (Intercept)                        -0.95048608  1.1750041 -0.80892152
## FMTFMT                              0.65388219  0.8508514  0.76850340
## SubGroup2                          -0.11515136  1.4591241 -0.07891814
## SubGroup3                           0.46997474  1.4713775  0.31941139
## SubGroup4                           1.04708617  1.4560313  0.71913712
## SubGroup5                          -0.41363139  1.3371475 -0.30933865
## SubGroup6                           0.06296588  1.4001464  0.04497093
## SubGroup7                          -2.21855783  1.7164311 -1.29254114
## SubGroup8                           0.40459074  1.2766910  0.31690577
## SubGroup9                          -1.35122066  1.7067358 -0.79169880
## BreedJersey                        -0.86539727  0.7891148 -1.09666844
## BreedJerseyCross                   -0.30200429  1.0030312 -0.30109162
## FMTControl:SampleType_day0Diarrheic -2.09925236  0.9945763 -2.11070018
## FMTFMT:SampleType_day0Diarrheic    -2.70851306  1.1234764 -2.41083211
##                                      Pr(>|t|)
## (Intercept)                        0.42035533
## FMTFMT                             0.44388105
## SubGroup2                          0.93724514
## SubGroup3                          0.75003734
## SubGroup4                          0.47362409
## SubGroup5                          0.75766551
## SubGroup6                          0.96421430
## SubGroup7                          0.19895405
## SubGroup8                          0.75193259
## SubGroup9                          0.43028791
```

```
## BreedJersey                         0.27524839
## BreedJerseyCross                    0.76392895
## FMTControl:SampleType_day0Diarrheic 0.03712807
## FMTFMT:SampleType_day0Diarrheic     0.01762059
##
## $`Escherichia/Shigella_uncultured`
##                                        Estimate Std. Error    t value
## (Intercept)                         -4.12573616  1.2964816 -3.1822558
## FMTFMT                              -0.35338602  0.9388165 -0.3764165
## SubGroup2                            2.59759740  1.6099753  1.6134393
## SubGroup3                            1.74379334  1.6234956  1.0740980
## SubGroup4                            1.66518382  1.6065628  1.0364885
## SubGroup5                            1.17827194  1.4753882  0.7986183
## SubGroup6                           -0.04774221  1.5449002 -0.0309031
## SubGroup7                            5.21922887  1.8938840  2.7558335
## SubGroup8                            3.87223571  1.4086814  2.7488371
## SubGroup9                            0.93029877  1.8831863  0.4940025
## BreedJersey                         -0.76636379  0.8706972 -0.8801725
## BreedJerseyCross                     1.86539595  1.1067294  1.6855032
## FMTControl:SampleType_day0Diarrheic -1.01002105  1.0974003 -0.9203761
## FMTFMT:SampleType_day0Diarrheic      0.84490935  1.2396268  0.6815836
##                                        Pr(>|t|)
## (Intercept)                         0.001913189
## FMTFMT                              0.707352649
## SubGroup2                           0.109594569
## SubGroup3                           0.285195011
## SubGroup4                           0.302311054
## SubGroup5                           0.426280857
## SubGroup6                           0.975404384
## SubGroup7                           0.006883210
## SubGroup8                           0.007021758
## SubGroup9                           0.622316994
## BreedJersey                         0.380738452
## BreedJerseyCross                    0.094805781
## FMTControl:SampleType_day0Diarrheic 0.359446624
## FMTFMT:SampleType_day0Diarrheic     0.496974863
##
```

```
## $Lactobacillus_reuteri
##                                       Estimate Std. Error    t value
## (Intercept)                          1.0815025  0.9275577  1.1659679
## FMTFMT                               3.4281885  0.6716689  5.1039855
## SubGroup2                           -2.0386049  1.1518443 -1.7698615
## SubGroup3                            0.3177506  1.1615173  0.2735652
## SubGroup4                           -0.8363639  1.1494028 -0.7276508
## SubGroup5                            0.9202177  1.0555550  0.8717857
## SubGroup6                           -0.1275096  1.1052868 -0.1153634
## SubGroup7                           -1.4323576  1.3549645 -1.0571182
## SubGroup8                           -0.1948784  1.0078301 -0.1933643
## SubGroup9                           -1.8972617  1.3473109 -1.4081840
## BreedJersey                         -1.1332117  0.6229336 -1.8191534
## BreedJerseyCross                     0.1639493  0.7918009  0.2070587
## FMTControl:SampleType_day0Diarrheic  0.1124042  0.7851265  0.1431669
## FMTFMT:SampleType_day0Diarrheic     -1.1886585  0.8868813 -1.3402678
##                                           Pr(>|t|)
## (Intercept)                         0.246219806818
## FMTFMT                              0.000001453376
## SubGroup2                           0.079598715050
## SubGroup3                           0.784946176125
## SubGroup4                           0.468416591607
## SubGroup5                           0.385277257883
## SubGroup6                           0.908373315672
## SubGroup7                           0.292838499742
## SubGroup8                           0.847040241685
## SubGroup9                           0.161975523968
## BreedJersey                         0.071684576031
## BreedJerseyCross                    0.836357514361
## FMTControl:SampleType_day0Diarrheic 0.886427605815
## FMTFMT:SampleType_day0Diarrheic     0.182997292972
##
## $Lactobacillus_salivarius
##                                        Estimate Std. Error     t value
## (Intercept)                         -0.23456830  1.1474695 -0.20442224
## FMTFMT                               0.34752074  0.8309129  0.41823966
## SubGroup2                            0.84064891  1.4249316  0.58995737
```

```
## SubGroup3                            0.71507304  1.4368979  0.49765056
## SubGroup4                            0.25326378  1.4219113  0.17811504
## SubGroup5                            0.16395310  1.3058133  0.12555630
## SubGroup6                           -0.01805403  1.3673360 -0.01320380
## SubGroup7                            0.25587184  1.6762090  0.15264913
## SubGroup8                           -0.71486324  1.2467736 -0.57337056
## SubGroup9                            2.34098254  1.6667409  1.40452699
## BreedJersey                          0.01617761  0.7706230  0.02099290
## BreedJerseyCross                    -0.01582375  0.9795266 -0.01615448
## FMTControl:SampleType_day0Diarrheic -0.51613161  0.9712698 -0.53139879
## FMTFMT:SampleType_day0Diarrheic     -0.14220178  1.0971494 -0.12961023
##                                      Pr(>|t|)
## (Intercept)                         0.8384119
## FMTFMT                              0.6766101
## SubGroup2                           0.5564630
## SubGroup3                           0.6197516
## SubGroup4                           0.8589694
## SubGroup5                           0.9003188
## SubGroup6                           0.9894898
## SubGroup7                           0.8789625
## SubGroup8                           0.5675969
## SubGroup9                           0.1630582
## BreedJersey                         0.9832904
## BreedJerseyCross                    0.9871412
## FMTControl:SampleType_day0Diarrheic 0.5962439
## FMTFMT:SampleType_day0Diarrheic     0.8971181
##
## $Bifidobacterium_pseudolongum
##                                        Estimate Std. Error     t value
## (Intercept)                          0.33095967  0.8391496  0.39439888
## FMTFMT                              -0.78479485  0.6076503 -1.29152376
## SubGroup2                           -0.21297494  1.0420589 -0.20437899
## SubGroup3                            0.46992103  1.0508099  0.44719891
## SubGroup4                            0.07982156  1.0398501  0.07676256
## SubGroup5                            1.08461892  0.9549471  1.13578948
## SubGroup6                            1.24729253  0.9999389  1.24736874
## SubGroup7                            1.12367651  1.2258192  0.91667396
```

```
## SubGroup8                            1.24002380  0.9117711  1.36001659
## SubGroup9                           -0.86294507  1.2188951 -0.70797323
## BreedJersey                          0.04017461  0.5635601  0.07128719
## BreedJerseyCross                     1.08784951  0.7163322  1.51863827
## FMTControl:SampleType_day0Diarrheic  0.13114128  0.7102940  0.18462958
## FMTFMT:SampleType_day0Diarrheic     -0.39173366  0.8023503 -0.48823274
##                                      Pr(>|t|)
## (Intercept)                         0.6940716
## FMTFMT                              0.1993051
## SubGroup2                           0.8384456
## SubGroup3                           0.6556354
## SubGroup4                           0.9389558
## SubGroup5                           0.2585823
## SubGroup6                           0.2149856
## SubGroup7                           0.3613750
## SubGroup8                           0.1766833
## SubGroup9                           0.4805012
## BreedJersey                         0.9433023
## BreedJerseyCross                    0.1318029
## FMTControl:SampleType_day0Diarrheic 0.8538691
## FMTFMT:SampleType_day0Diarrheic     0.6263840
##
## $Others
##                                        Estimate Std. Error     t value
## (Intercept)                         -0.98678270  0.7122665 -1.38541215
## FMTFMT                               0.09167518  0.5157710  0.17774398
## SubGroup2                            0.53051441  0.8844950  0.59979355
## SubGroup3                            1.08820067  0.8919228  1.22006146
## SubGroup4                            0.06607496  0.8826202  0.07486228
## SubGroup5                            1.06500528  0.8105550  1.31392111
## SubGroup6                            2.40904349  0.8487438  2.83836368
## SubGroup7                            1.31931748  1.0404699  1.26800152
## SubGroup8                            1.76913280  0.7739073  2.28597506
## SubGroup9                            0.72486960  1.0345928  0.70063276
## BreedJersey                         -0.52709725  0.4783473 -1.10191328
## BreedJerseyCross                    -0.50055761  0.6080196 -0.82325893
## FMTControl:SampleType_day0Diarrheic  0.06206888  0.6028944  0.10295149
```

```
## FMTFMT:SampleType_day0Diarrheic     -0.25708606  0.6810314 -0.37749517
##                                        Pr(>|t|)
## (Intercept)                         0.168807721
## FMTFMT                              0.859260098
## SubGroup2                           0.549911895
## SubGroup3                           0.225123807
## SubGroup4                           0.940464072
## SubGroup5                           0.191682787
## SubGroup6                           0.005426747
## SubGroup7                           0.207549584
## SubGroup8                           0.024225813
## SubGroup9                           0.485052997
## BreedJersey                         0.272971822
## BreedJerseyCross                    0.412191859
## FMTControl:SampleType_day0Diarrheic 0.918194174
## FMTFMT:SampleType_day0Diarrheic     0.706553385
##FMT and health
fmt$Breed <- as.factor(fmt$Breed)
fmt$FMT <- factor(fmt$FMT, levels = c("Control","FMT"))
fmt$SampleType_day0 <- factor(fmt$SampleType_day0, levels = c("Healthy", "Diarrheic"))
fmt$SampleType_day10 <- factor(fmt$SampleType_day10, levels = c("Healthy", "Diarrheic"
))


summary(glm(SampleType_day10 ~ (SampleType_day0 + FMT)^2, data = fmt, family="binomial
"))
##
## Call:
## glm(formula = SampleType_day10 ~ (SampleType_day0 + FMT)^2, family = "binomial",
##     data = fmt)
##
## Deviance Residuals:
##     Min       1Q   Median       3Q      Max
## -0.9005  -0.8067  -0.7325  -0.2801   2.5527
##
## Coefficients:
##                                   Estimate Std. Error z value Pr(>|z|)
## (Intercept)                        -0.9555     0.3721  -2.568   0.0102 *
```

```
## SampleType_day0Diarrheic         -2.2634     1.0852  -2.086   0.0370 *
## FMTFMT                            0.2624     0.4956   0.529   0.5965
## SampleType_day0Diarrheic:FMTFMT   1.7779     1.2696   1.400   0.1614
## ---
## Signif. codes:  0 '***' 0.001 '**' 0.01 '*' 0.05 '.' 0.1 ' ' 1
##
## (Dispersion parameter for binomial family taken to be 1)
##
##     Null deviance: 133.27  on 120  degrees of freedom
## Residual deviance: 123.04  on 117  degrees of freedom
## AIC: 131.04
##
## Number of Fisher Scoring iterations: 5
pred <- unique(model.frame(~ (SampleType_day0 + FMT)^2, data = fmt))
pred$y_hat <- unique(predict(glm(SampleType_day10 ~ (SampleType_day0 + FMT)^2, data =
fmt, family="binomial"), type="response"))
pred
##    SampleType_day0     FMT      y_hat
## 1          Healthy     FMT 0.33333333
## 2          Healthy Control 0.27777778
## 9        Diarrheic Control 0.03846155
## 13       Diarrheic     FMT 0.23529412
```

#Relative abundance plot - FMT product

```
library("readxl")
otu_mat_fmt <- read_excel("otu_mat_fmt.xlsx")
otu_mat_fmt <- as.data.frame(otu_mat_fmt)


tax_mat_fmt <- read_excel("tax_mat_fmt.xlsx")
tax_mat_fmt<- as.data.frame(tax_mat_fmt)


samplescalves_fmt <- read_excel("samples_fmt.xlsx")
samples_df_fmt <- as.data.frame(samplescalves_fmt)
```

#Define the row names from the otu column

```
row.names(otu_mat_fmt) <- otu_mat_fmt$otu
```

#Remove the column otu since it is now used as a row name

```
otu_mat_fmt$otu <- NULL
```

#Idem for the two other matrixes

```
  row.names(tax_mat_fmt) <- tax_mat_fmt$otu
  tax_mat_fmt$otu <- NULL
```

#Transform into matrixes otu and tax tables (sample table can be left as data frame)

```
  otu_mat_fmt <- as.matrix(otu_mat_fmt)
  colnames(otu_mat_fmt) <- as.character(otu_mat_fmt[1,]) # I added this to make zymo i
d as header
  class(otu_mat_fmt) <- "numeric" # I added this to change to numeric
  otu_mat_fmt = otu_mat_fmt[-1,] #Deleting 1st row that is NA
  tax_mat_fmt <- as.matrix(tax_mat_fmt)
```

#Transform to phyloseq objects

```
  OTU_fmt = otu_table(otu_mat_fmt, taxa_are_rows = TRUE)
  TAX_fmt = tax_table(tax_mat_fmt)

  row.names(samples_df_fmt) <- samples_df_fmt$sample
  samples_df_fmt$sample <- NULL
  meta.df_fmt <- samples_df_fmt
  samples_fmt = sample_data(meta.df_fmt)


dta_fmt <- phyloseq(OTU_fmt, TAX_fmt, samples_fmt)
dta_fmt
## phyloseq-class experiment-level object
## otu_table()   OTU Table:         [ 15 taxa and 3 samples ]
## sample_data() Sample Data:       [ 3 samples by 10 sample variables ]
## tax_table()   Taxonomy Table:    [ 15 taxa by 2 taxonomic ranks ]
#Making a barplot at the species level for the FMT product

rel_produc = transform_sample_counts(dta_fmt, function(x) x / sum(x) )
rel_produc
## phyloseq-class experiment-level object
```

```
## otu_table()   OTU Table:         [ 15 taxa and 3 samples ]
## sample_data() Sample Data:       [ 3 samples by 10 sample variables ]
## tax_table()   Taxonomy Table:    [ 15 taxa by 2 taxonomic ranks ]
glom <- tax_glom(rel_produc, taxrank = 'Species')
glom
## phyloseq-class experiment-level object
## otu_table()   OTU Table:         [ 15 taxa and 3 samples ]
## sample_data() Sample Data:       [ 3 samples by 10 sample variables ]
## tax_table()   Taxonomy Table:    [ 15 taxa by 2 taxonomic ranks ]
data_glom<- psmelt(glom) # create dataframe from phyloseq object
data_glom$Species <- as.character(data_glom$Species) #convert to character

#Count species to set color palette
Count = length(unique(data_glom$Species))
Count
## [1] 15
#Adding 15 colors (15 species)
library(RColorBrewer)
nb.cols <- 15
mycolors <- colorRampPalette(brewer.pal(8, "Set1"))(nb.cols)

#Changing the scale to 0-100 of Abundance
data_glom2 <- data_glom %>% mutate(Abundance = Abundance * 100)
g <- ggplot(data_glom2, aes(x = Sample, y = Abundance))
g + geom_bar(aes(fill = Species), stat = "identity") +
scale_fill_manual(values = mycolors) + xlab("Sample") + ylab("Relative Abundance (%)")
+ theme_bw()
```

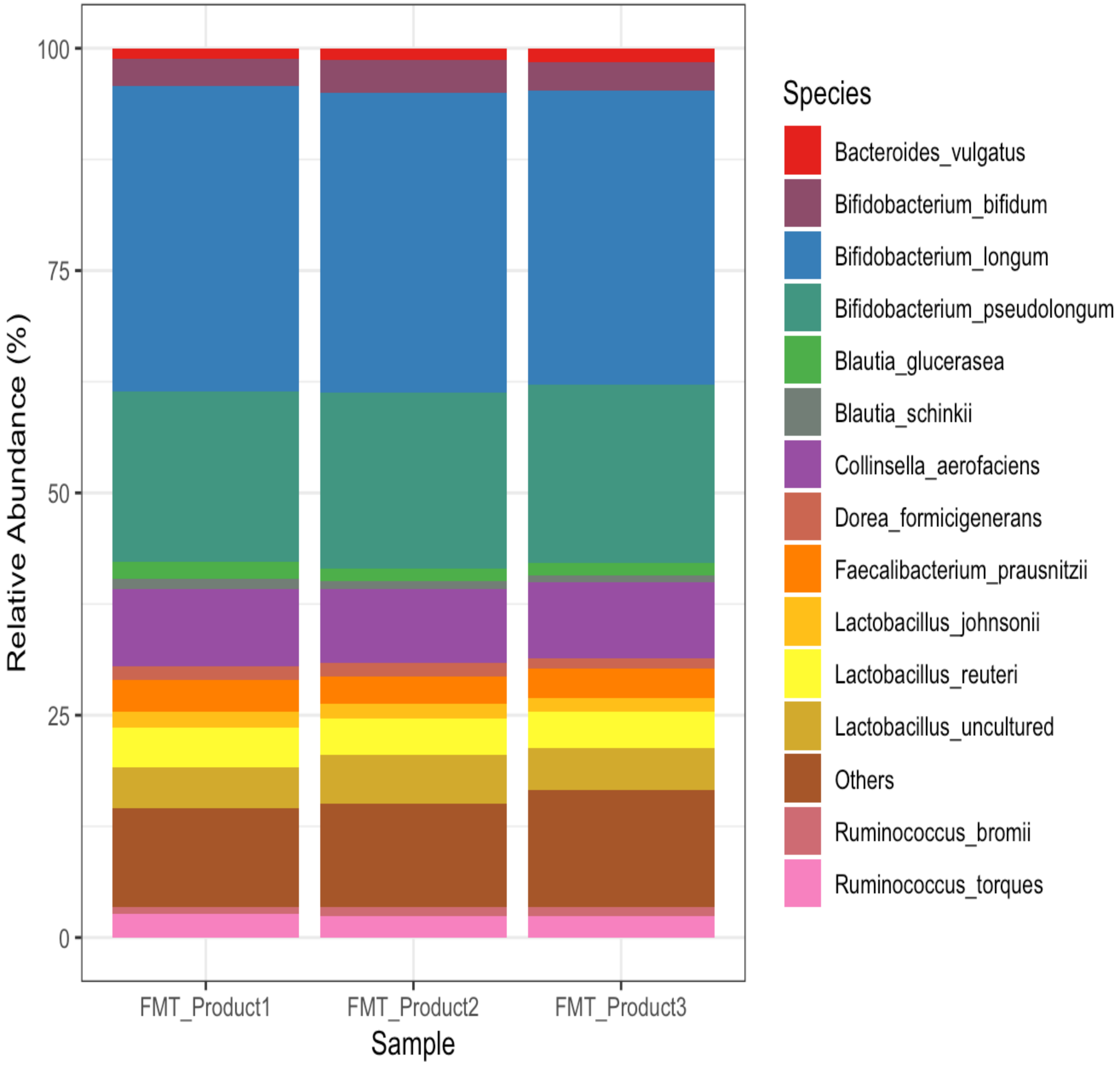


```
glom <- tax_glom(rel_produc, taxrank = 'Phylum')
glom
## phyloseq-class experiment-level object
## otu_table()   OTU Table:         [ 15 taxa and 3 samples ]
## sample_data() Sample Data:       [ 3 samples by 10 sample variables ]
## tax_table()   Taxonomy Table:    [ 15 taxa by 2 taxonomic ranks ]
data_glom<- psmelt(glom) # create dataframe from phyloseq object
data_glom$Species <- as.character(data_glom$Phylum) #convert to character
```

```
#Count species to set color palette
Count = length(unique(data_glom$Species))
Count
## [1] 6
#Adding 6 colors (6 species)
library(RColorBrewer)
nb.cols <- 6
mycolors <- colorRampPalette(brewer.pal(8, "Set1"))(nb.cols)

#Changing the scale to 0-100 of Abundance
data_glom2 <- data_glom %>% mutate(Abundance = Abundance * 100)
g <- ggplot(data_glom2, aes(x = Sample, y = Abundance))
g + geom_bar(aes(fill = Phylum), stat = "identity") +
scale_fill_manual(values = mycolors) + xlab("Sample") + ylab("Abundance") + theme_bw()
```

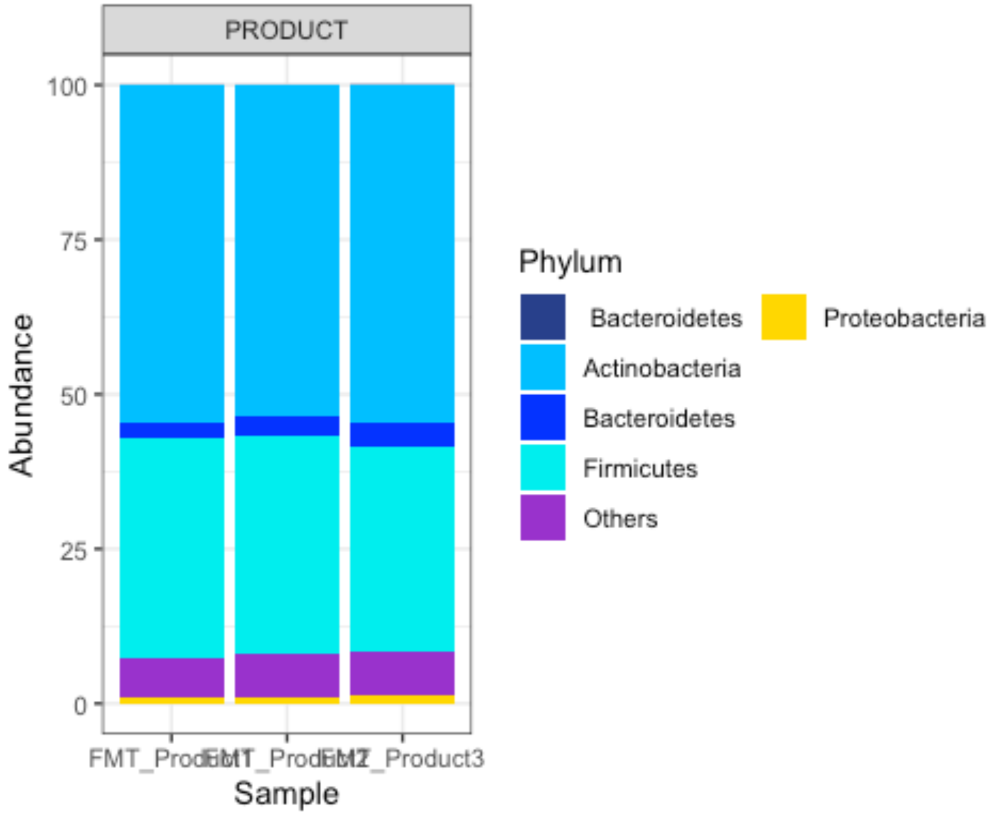

Supplement: S1 Appendix — (PDF) [file pone.0276638.s001.pdf]
